# Differential Regulation of Effector- and Central-Memory Responses to *Toxoplasma gondii* Infection by IL-12 Revealed by Tracking of Tgd057-Specific CD8+ T Cells

**DOI:** 10.1371/journal.ppat.1000815

**Published:** 2010-03-19

**Authors:** Douglas C. Wilson, Gijsbert M. Grotenbreg, Kenian Liu, Yanlin Zhao, Eva-Maria Frickel, Marc-Jan Gubbels, Hidde L. Ploegh, George S. Yap

**Affiliations:** 1 Center for Immunity and Inflammation, UMDNJ-New Jersey Medical School, Newark, New Jersey, United States of America; 2 Immunology Programme and Departments of Microbiology and Biological Sciences, National University of Singapore, Singapore; 3 Whitehead Institute for Biomedical Research, Cambridge, Massachusetts, United States of America; 4 Department of Biology, Boston College, Chestnut Hill, Massachusetts, United States of America; Cornell University, United States of America

## Abstract

Production of the pro-inflammatory cytokine IL-12 by innate phagocytes drives the differentiation of IFN-γ-producing effector T cells during *Toxoplasma gondii* infection. However, the role of IL-12 in the regulation of memory CD8+ T cell differentiation and function during murine toxoplasmosis is unclear. To track memory CTL development, we identified a novel H-2K^b^-restricted CTL population specific for the *Toxoplasma* antigen tgd057. Tgd057-specific CTLs were induced by both vaccination and natural peroral infection, and were representative of the polyclonal CTL population. Tgd057-specific primary effector cells required IL-12 for the differentiation of KLRG1+ effector subpopulations and IFN-γ production in response to restimulation with parasite-infected cells, but not to restimulation with cognate peptide. The effect of IL-12 deficiency during the primary response was profoundly imprinted on memory CTLs, which continued to show defects in cell numbers, KLRG1+ effector memory subpopulation differentiation, and IFN-γ recall responses. Importantly, isolated CD62L^hi^ KLRG1- CD8+ T cells differentiated in the absence of IL-12 were enhanced in their ability to generate IFN-γ-producing secondary tgd057-specific effector cells. Our data, for the first time, demonstrate the negative impact of IL-12 signaling on the quality of the central memory CTL compartment. Thus, despite the beneficial role of IL-12 in promoting effector differentiation, excessive exposure to IL-12 during CTL priming may limit the development of long-term protective immunity through the decreased fitness of central memory CTL responses.

## Author Summary

*Toxoplasma gondii* is a ubiquitous protozoan parasite that causes severe disease in people with compromised immune function. It is known that CD8+ T cells are essential for the establishment of protective immunity, primarily through the delivery of the effector cytokine interferon-γ (IFN-γ) to *Toxoplasma*-infected cells. However, it remains unclear how memory CD8+ T cells develop in response to *Toxoplasma* infection, and to what extent inflammatory cytokines like interleukin-12 (IL-12) play a role in memory development. Furthermore, the natural *T. gondii* antigens that induce CD8+ T cell activation have not yet been fully uncovered. Using new technology for the screening of antigen specificity, we discovered the first natural antigen-specific CD8+ T cell population induced by *T. gondii* infection in C57BL/6 mice. By tracking natural parasite-specific responses, we found that IL-12 plays a vital role in promoting the development of IFN-γ-producing effector memory CD8+ T cells but at a cost to the numbers and function of central memory CD8+ T cells.

## Introduction

Infection by the intracellular protozoan parasite *Toxoplasma gondii* causes severe disease in immunocompromised individuals including neonates, transplant recipients, and people with AIDS [1]. In healthy individuals however, resistance to toxoplasmosis is mediated by the ability of T lymphocytes to produce the essential effector cytokine IFN-γ [2],[3]. Although both innate and adaptive immunity synergize to combat parasitic infection [4],[5], CD8+ T cells are known to be the main mediator of protective immunity [6],[7],[8],[9]. The importance of CTL responses is exemplified by the differential susceptibility of inbred strains of mice to toxoplasmic encephalitis, where resistant strains are protected by CTLs responding to antigens presented by H-2L^d^ MHC class I products [10]. Unfortunately, the natural *Toxoplasma* epitopes that drive CTL activation remain incompletely defined and thus encumber our ability to track the development of Ag-specific CTLs.

The *Toxoplasma* antigens historically identified as CTL targets were defined by the vaccination of mice with purified antigens or DNA expression vectors. The *T. gondii* surface antigen SAG1 (p30) was the first isolated Ag shown to elicit CTL effector responses [11] and protective immunity in mice [12]. SAG1 DNA vaccination also protected mice from lethal parasite challenge [13]. DNA vaccination using the secreted *Toxoplasma* proteins GRA1, GRA7, and ROP2 also elicits partially protective immunity [14],[15]. Nonetheless, the precise CTL-specific peptide epitopes derived from these candidate antigens have yet to be identified. Recently, an unbiased epitope discovery screen identified a single H-2L^d^-restricted, GRA6 epitope-specific CTL population elicited by *T. gondii* infection in BALB/c mice [16]. Although these CTLs were argued to be an immunodominant population, another report has emerged describing two H-2L^d^-restricted CD8+ T cell populations specific for the GRA4 and ROP7 *T. gondii* antigens [17]. The latter study utilized caged MHC class I tetramers that allow the exchange of a conditional ligand occupying the MHC molecule for putative antigenic epitopes (pre-selected by a targeted epitope prediction program), and subsequently used large numbers of freshly generated tetramers to screen for Ag-specific CTLs [18]. Since both of these studies involved mice of the H-2^d^ haplotype, and most basic immunological research uses C57BL/6 mice of the H-2^b^ haplotype [19], we chose to screen for putative *Toxoplasma* CTL epitopes in C57BL/6 mice. Using caged MHC class I tetramers, we identified a single genuine H-2K^b^-restricted epitope derived from the protein tgd057.

Having identified a novel population of *Toxoplasma*-specific CD8+ T cells, we sought to extend our previous findings regarding the role of IL-12 for the differentiation of effector and memory CTLs. *T. gondii* infection induces a robust type 1 polarized immune response marked by the production of the pro-inflammatory cytokine IL-12 by macrophages and dendritic cells [20]. It is clear that in CD4+ T_H_1 cells, IL-12 signaling is required for the activation of IFN-γ production [21]. However in CD8+ T cells, the impact of IL-12 signaling on effector and memory responses to viral, bacterial, and parasitic infections has only recently come to light. We have previously shown that among the polyclonal CTL populations induced after *T. gondii* vaccination, IL-12 is strictly required for IFN-γ production and KLRG1+ effector subpopulation differentiation in vivo [22]. KLRG1 expression in CD8+ T cells is thought to mark replicative senescence in a subset of highly activated CTLs [23],[24],[25]. Furthermore, IL-12 can induce KLRG1 through the activation of T-bet, a master regulator of the type 1 immune program [26]. Therefore, it is thought that primary pathogen challenge induces KLRG1 in IL-12-sensitive CD8+ T cells that, while being highly activated effector cells, are short-lived and do not contribute to secondary memory responses.

As a consequence of the effector program initiated by IL-12 signaling during primary CTL activation, memory precursor CTL development may be negatively impacted. IL-12 deficiency was shown to increase memory CTL precursor frequency in the primary response to *L. monocytogenes* and LCMV [26],[27], and to impart superior immunity to bacterial re-infection [27]. Unlike bacterial and viral systems where multiple innate cytokines can promote CTL effector differentiation [28],[29], in *T. gondii* infection, IL-12 appears to singularly control CD8+ T cell differentiation and IFN-γ production [22],[30],[31]. Given the absolute requirement for IFN-γ in CTL protective immunity to *T. gondii*, it is difficult to assess whether memory responses will be enhanced under conditions of complete and permanent IL-12 deficiency. Therefore, to determine to what extent IL-12 negatively impacts memory response to the parasite, we examined the effects of IL-12 insufficiency during primary CTL activation on subsequent effector and memory responses recalled in the presence of IL-12. Interestingly, the requirement of IL-12 for IFN-γ production during the primary response was imprinted only on effector memory cells during an immediate recall response. However, central memory tgd057-specific CTLs, primed in the absence of IL-12 but recalled in an IL-12 competent environment, were able to repopulate the secondary immune response with IFN-γ producing effector memory cells. Our results indicate that IL-12 has divergent consequences for heterogeneous CD8+ subpopulations. Whereas IL-12 is required for effector memory differentiation of KLRG1+ subsets, it is not required for central memory differentiation, and may even negatively impact T_CM_ function.

## Results

### Identification of a novel H-2K^b^-restricted CD8+ T cell population specific for the *Toxoplasma* antigen tgd057

**Figure 1:**
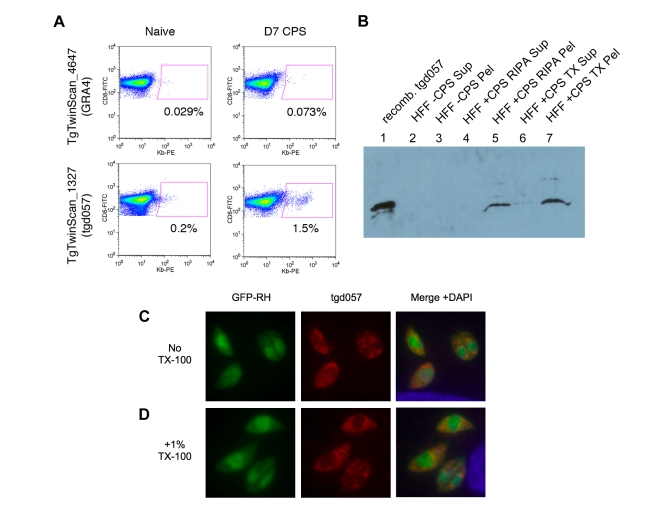
Tgd057 localizes to the parasite's cytoskeleton. *A*) Example output data from the screening of putative CTL epitopes derived from GRA4, *upper panels*, and tgd057, *lower panels*. The GRA4-epitope tetramer was scored as negative for binding to day 7 CPS-primed CD8α+ T cells, whereas the tgd057-epitope tetramer (K^b^/SVLAFRRL) was scored as positive. *B*) Western blot of CPS parasite cultures using purified anti-tgd057 Abs. Lane 1, 0.15 µg of recombinant tgd057. Lanes 2–3, supernatant and pellet, respectively, of an uninfected HFF culture extracted in mRIPA buffer. Lanes 4–5, supernatant and pellet, respectively, of a CPS-infected HFF culture extracted in mRIPA buffer. Lanes 6–7, supernatant and pellet, respectively, of a CPS-infected HFF culture extracted in 1% Triton X-100 buffer. *C* and *D*) Immunofluorescence images of HFFs infected with GFP-RH parasites for 4 hours. *C*, Cells were fixed and stained with anti-tgd057 Abs, and the nuclear stain DAPI. *D*, Cells were fixed, treated with 1% Triton X-100 for 20 min, and then stained as in *C*.

Given that the *T. gondii* secreted proteins GRA1, GRA7, ROP1, ROP2, and the parasite surface protein SAG1 have previously been shown to elicit CTL responses in C57BL/6 mice, we initially screened these proteins for putative CTL epitopes. Their predicted protein sequences were analyzed for peptide epitopes using a program that combines four different prediction routines to choose the octameric or nonameric peptide sequences with the highest potential for binding either H-2K^b^ or H-2D^b^ molecules, respectively [32]. These putative peptide epitopes (Table S1) were synthesized and individually loaded onto the corresponding MHC class I tetramers [33],[34] using caged MHC class I tetramer technology [18],[35]. Peptide-MHC tetramers were screened for binding to purified CD8+ T cells harvested from spleens of CPS-vaccinated mice. Purified naïve CD8α+ splenocytes were used as a negative control. From this first screen, we were unable to confirm the presence of a genuine CTL epitope (data not shown). Therefore, we screened for peptide epitopes from a second, more extensive set of *Toxoplasma* proteins using the same method (Tables S2 and S3). Included in the second screen was a large set of secreted proteins (both confirmed and uncharacterized but likely secreted based on sequence) as well as a few non-secreted proteins [17]. This second screen resulted in the discovery of a single genuine CTL epitope – SVLAFRRL – derived from the gene *tgd057* (Figure 1A), which encodes a putatively secreted protein of unknown function [36].

From its initial characterization, Wan et al reported that the *tgd057* gene sequence encoded a secretion signal peptide (residues 1–21), which would allow for trafficking to the three secretory organelles: micronemes, rhoptries, or dense granules. Since *tgd057* contains no other known targeting motif, we predicted that tgd057 would reside in the dense granules, the default pathway for the constitutive secretion of proteins into the parasitophorous vacuole [37]. To establish the localization of tgd057, we first generated polyclonal antisera against recombinant tgd057. Anti-tgd057 antibodies were further purified from the antisera by reactivity to recombinant protein bound to nitrocellulose and extraction from tgd057+ bands. To demonstrate the specificity of our purified, polyclonal anti-tgd057 Abs, we compared their reactivity to recombinant tgd057 protein, uninfected HFF lysates, and CPS-infected HFF lysates. To further characterize tgd057, we performed the protein extraction using two different buffers: modified RIPA and 1% Triton X-100. The anti-tgd057 Abs showed no reaction to either the supernatant or pellet of mRIPA-extracted, uninfected HFF lysates (Figure 1B, *lanes 2 and 3*). However, there is a clear band in the pellet but not supernatant of the mRIPA-extracted CPS-infected HFF lysate (Figure 1B, *lanes 4 and 5*), corresponding to a band in the recombinant tgd057 lane (Figure 1B, *lane 1*). Upon Triton X-100 extraction of the CPS-infected HFF lysate, the majority of tgd057 partitioned to the insoluble fraction. (Figure 1B, *lanes 6 and 7*). Moreover, multiple minor tgd057 bands with slower mobility were also visible in the mRIPA- and Triton X-100-insoluble CPS-infected HFF lysates, suggesting presence of tgd057 isoforms. Overall, the insolubility of tgd057 in mRIPA and Triton X-100 buffers suggests that tgd057 is associated with membrane insoluble structures such as the parasite's cytoskeleton, although some may be trafficked to membrane soluble structures such as the secretory organelles.

To further characterize the localization of tgd057, immunofluorescence staining was performed on HFFs infected with GFP-expressing RH parasites. As depicted in Figure 1C, the staining pattern is unlike that of the secretory organelles and appears to stain structural elements near the apical complex and also at the posterior end of the parasite. To confirm that tgd057 is resistant to detergent extraction as suggested by Figure 1B, infected HFFs were treated with 1% Triton X-100 to remove membrane-associated proteins prior to staining. In Triton X-100-treated samples, tgd057 staining is clearly retained (Figure 1D).

### Tgd057-specific CD8+ T cells are effector cells phenotypically representative of the polyclonal CTL population

**Figure 2:**
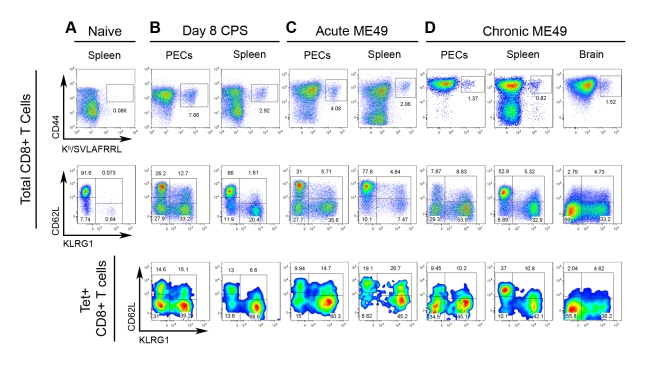
Tgd057-specific CTLs are induced by CPS vaccination and natural ME49 infection. Samples from *A*) naïve mice and *B–D*) *T. gondii* infected mice were stained for CD44 and K^b^/SVLAFRRL positivity and the expression of CD62L and KLRG1 in CD8+ T cells ex vivo. *Upper row*, frequency of CD44^hi^ K^b^/SVLAFRRL+ cells of total CD8α+ TCRβ+ cells. *Middle row*, total CD8α+ cells were analyzed for CD62L and KLRG1 expression. *Lower row*, K^b^/SVLAFRRL+ CD8α+ cells were analyzed for CD62L and KLRG1 expression. *A*) Spleens were harvested from naïve mice. *B*) Mice were vaccinated with CPS parasites and PECs and spleens were harvested on day 8 post-vaccination. *C*) PECs and spleens were harvested on day 7 post-infection of mice perorally infected with 20 ME49 cysts. *D*) PECs, spleens, and the brain mononuclear cells were analyzed in day 48 cyst-infected mice (as in *C*). One representative mouse was shown of at least three samples. For the PEC and spleen analysis in *A–D*, one of at least three independent experiments is shown. For the brain mononuclear cell analysis in *D*, one of two experiments is shown.

Although the precise localization and function of tgd057 remains to be elucidated, it is clear from our screen that tgd057 is available for processing and presentation to CD8+ T cells in vivo. To ascertain the relationship between epitope-specific CD8+ T cells and the polyclonal response to *T. gondii* infection, the numbers and activation phenotype of tgd057-specific cells were compared to that of the total CD8+ T cell population after vaccination or infection with avirulent parasites. Mice were vaccinated with a single dose of attenuated, CPS-mutant parasites known to elicit protective immunity [38],[39]. CPS vaccination primed CD44^hi^ tgd057-specific CTLs, which constituted 7.66% of the total PEC CD8+ T cells and 2.92% of the total splenic CD8+ T cells on day 8 post-vaccination (Figure 2A and 2B). As we have previously published [22], the polyclonal CTL response is composed of four subpopulations based on the expression of the cell surface activation markers–CD62L and KLRG1. The four subpopulations are termed Fraction (F) I (CD62L^hi^ KLRG1-), FII (CD62L^lo^ KLRG1-), FIII (CD62L^lo^ KLRG1+), and FIV (CD62L^hi^ KLRG1+). FI was thought to contain undifferentiated CD8+ T cells due to the lack of granzyme B and IFN-γ expression during the primary effector response. Furthermore, the FI phenotype is shared by the majority of CD8+ T cells in the spleens of naïve mice (Figure 2A). The other three subpopulations induced by *Toxoplasma* infection contained varying levels of granzyme B+ and IFN-γ+ effector cells, with FIII representing the most effector cell-rich subpopulation [22]. When the PEC tgd057-specific cells were analyzed for CD62L and KLRG1 expression after CPS vaccination, all four subpopulations were represented, including the seemingly undifferentiated FI subset (Figure 2B). In the spleens of these mice, all four subpopulations of tgd057-specific CTLs were also present, although mature FIII phenotype cells were over-represented. To view the immune response to natural *Toxoplasma* infection, mice were perorally infected with cysts of the avirulent ME49 strain, which not only acutely infects the host, but also establishes chronic infection in the brain and muscle tissue. During the acute phase of ME49 infection, CD44^hi^ tgd057-specific CD8+ T cells were present both in the PECs and in the spleen at a comparable frequency to CPS vaccination (Figure 2C), however ME49 infection induced fewer numbers of tgd057-specific CTLs in these tissues (Figure S1). Heterogeneous effector CTL subpopulations were similarly induced by ME49 infection, but the tgd057-specific CTLs were particularly enriched in FIII phenotype cells, both in the PECs and the spleen. During the chronic phase of ME49 infection, the frequencies and numbers of PEC and splenic CD44^hi^ tgd057-specific CTLs were reduced but still detectable (Figure 2D and S1). In all three tissues sampled, the tgd057-specific CTLs were representative of the total CD8+ T cell population. Interestingly, FIII CTLs remained a major subpopulation both in the spleen and in the periphery, suggesting that KLRG1 positivity can indeed be a stable, long-lasting phenotype. A significant pool of tgd057-specific CTLs was found in the brains of chronically infected mice, a site of spontaneous *T. gondii* recrudescence, where FII and FIII were the predominant subpopulations (Figure 2D and S1). In general, tgd057-specific CTLs were phenotypically similar to the polyclonal CD8+ T cell population, except during acute infection, where mature effector-phenotype cells were enriched and the naïve-like subpopulation was diminished in the spleen.

**Figure 3:**
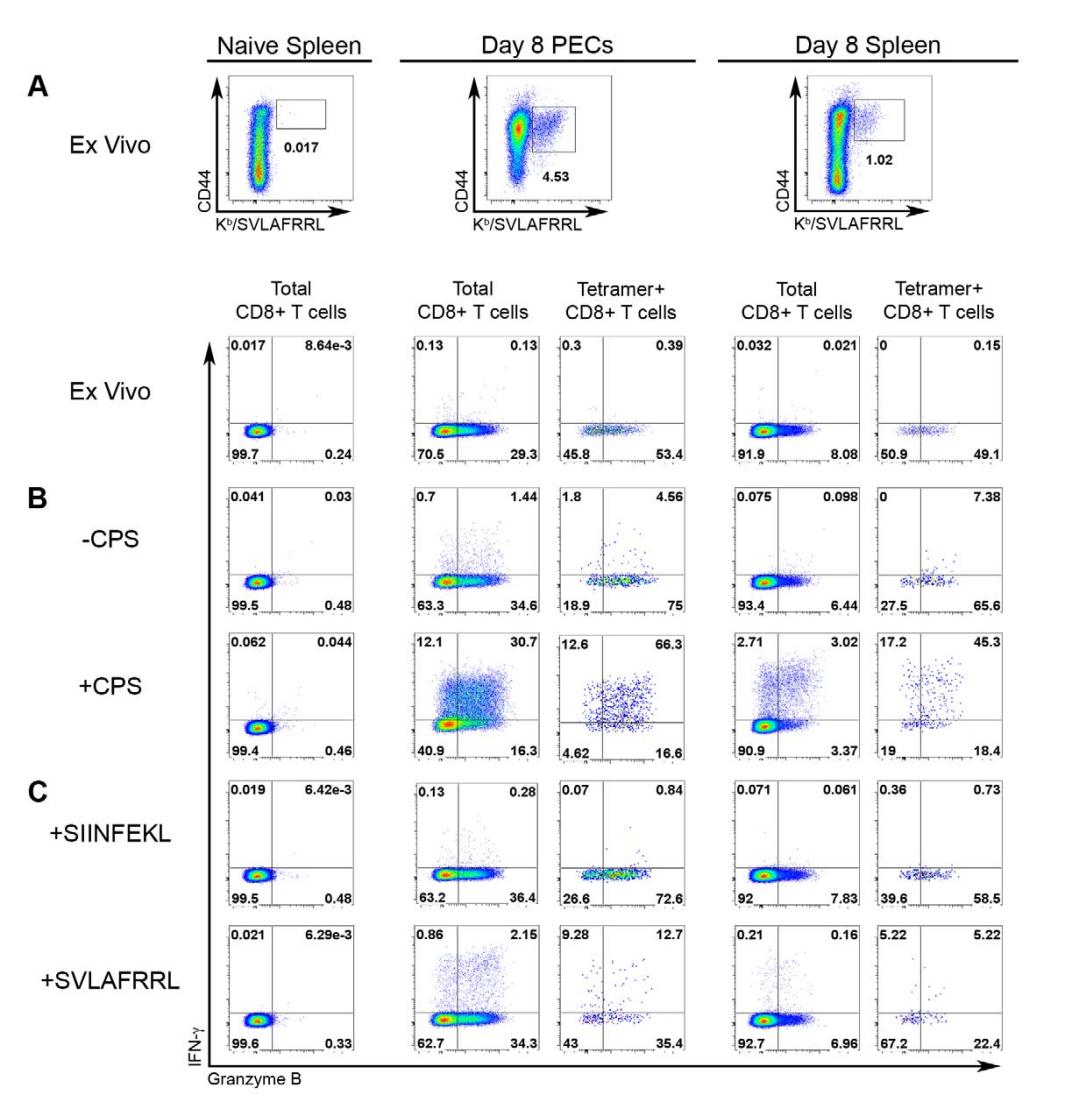
Effector molecule expression among tgd057-specific CTLs. Expression of IFN-γ and granzyme B was analyzed in CD8+ T cells from naïve and day 8 CPS-primed mice. *A*) The frequencies of tgd057-specific CTLs (CD44^hi^, K^b^/SVLAFRRL+, CD8α+, and TCRβ+ cells) was compared between naïve and primed samples ex vivo, *upper row*. IFN-γ and granzyme B expression was evaluated between CD8+ T cells from the naïve spleen, and total CD8+ T cells and tgd057-specific (Tetramer+) CD8+ T cells from day 8 CPS-primed PECs and splenocytes, *lower row*. For each of these populations, IFN-γ and granzyme B expression was analyzed after CPS restimulation (*B*) and peptide restimulation (*C*). *B*) Samples were either unstimulated (*upper row*) or restimulated (*lower row*) with CPS parasites. *C*) Samples were directly incubated with either non-cognate (SIINFEKL, *upper row*) or cognate (SVLAFRRL, *lower row*) peptide. FACS plots represent one of three mice. One of three independent experiments is shown.

Analysis of effector molecule expression between the epitope-specific and total CD8+ T cell populations would provide a direct assessment their relative effector capability. Therefore, granzyme B and IFN-γ production were measured in both tgd057-specific CTLs and the total CD8+ T cell population. Effector cells from day 8 CPS-primed mice were compared to naïve splenocytes and evaluated for granzyme B and IFN-γ co-expression ex vivo and in response to live parasite infection or peptide restimulation (Figure 3). Tgd057-specific CTLs were highly enriched in granzyme B+ effector cells ex vivo; 54% and 49% of the PEC and splenic tgd057-specific CTLs were granzyme B+, respectively, whereas in the total PEC and splenic CD8+ T cells, 29% and 8% were granzyme B+, respectively (Figure 3A). After restimulation with CPS parasites both IFN-γ and granzyme B were upregulated in total PEC and splenic CD8+ T cells, of which the tgd057-specific CTLs remained enriched in frequency of effector molecule-positive cells (Figure 3B). Whole PECs and spleen cultures were also subjected to peptide restimulation to demonstrate the specificity of tgd057-specific CTLs. As shown in Figure 3C, IFN-γ was induced by both PEC and splenic CD8+ T cells in the presence of cognate peptide (SVLAFRRL) but not non-cognate peptide (SIINFEKL). The frequency of tgd057-specific cells and thus K^b^/SVLAFRRL+ IFN-γ+ cells was reduced after cognate peptide restimulation, a response we attributed to TCR downregulation, a well-characterized response to strong TCR ligation [40]. Taken together, tgd057-specific CTLs are highly enriched in granzyme B+ effector cells and produce the protective cytokine IFN-γ in response to live parasites and cognate peptide.

### IL-12 is required for effector differentiation in tgd057-specific CTLs

**Figure 4:**
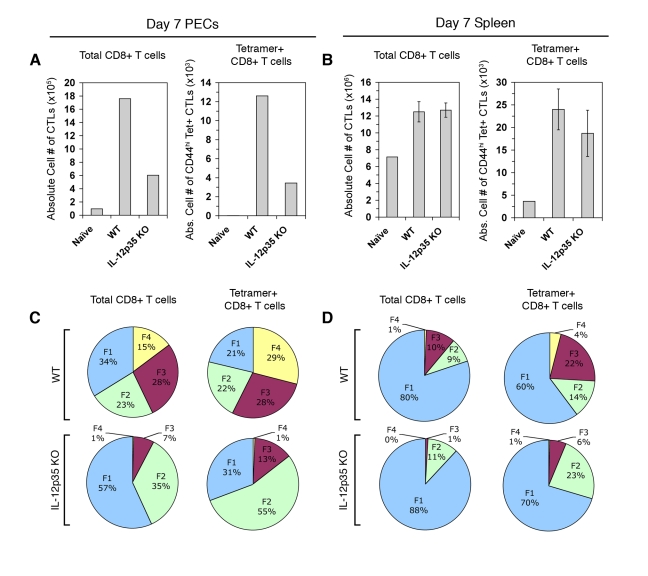
Primary CTL activation and differentiation in *Il12a* ^−/−^ mice. PECs and spleens were analyzed for CTL activation on day 7 post-vaccination in WT and *Il12a* ^−/−^ mice. *A* and *B*) Total CD8α+ TCRβ+ cells and CD44^hi^ K^b^/SVLAFRRL+ CD8+ T cells were enumerated in PECs (*A*) and splenocytes (*B*) of WT naïve, WT primed, and *Il12a* ^−/−^ primed mice. *C* and *D*) Subpopulation distribution is shown in total CD8α+ cells and K^b^/SVLAFRRL+ CD8α+ cells in PECs (*C*) and splenocytes (*D*) of WT and *Il12a* ^−/−^ primed mice. PEC samples were pools of three mice. Bar graphs are shown as mean ± SEM of three mice. Pie charts are shown as mean values for the spleen samples. Data are representative of three independent experiments.

We have previously demonstrated that the innate cytokine IL-12 produced during *Toxoplasma* vaccination induces CTL IFN-γ production and suggested that IL-12 promotes terminal maturation at the expense of memory precursor subpopulation differentiation [22]. A limitation of our earlier study was that putative parasite-reactive CD8+ T cells were phenotypically defined by activation markers (CD62L downregulation and KLRG1 upregulation) and effector molecule expression (granzyme B and IFN-γ), rather than by parasite-antigen specificity. By definition, we were not able to detect parasite-antigen specific CTLs or CTL precursors that have yet to express activation markers or effector molecules. Utilizing tetramer staining we are now able to resolve the totality of the natural tgd057-specific CTL response, which includes a significant proportion of undifferentiated FI-phenotype cells (Figure 2B–2D). This allows us to address the question of whether IL-12 truly negatively impacts the KLRG1- putative memory precursor compartment, which encompasses T_CM_-like FI (CD62L^hi^ KLRG1-) and T_EM_-like FII (CD62L^lo^ KLRG1-) precursor cells. To firmly address the requirements of IL-12 during the primary activation of parasite-specific CTLs, tgd057-specific cells were enumerated and phenotyped in WT and *Il12a*(IL-12p35)^−/−^ CPS-primed mice. On day 7 post-vaccination, the numbers of total and tgd057-specific CTLs were 3-fold lower in PECs of *Il12a* ^−/−^ mice (Figure 4A). In the spleen, the total numbers of CD8+ T cells were unchanged in *Il12a* ^−/−^ mice, although the numbers of tgd057-specific CTLs were slightly lower on average (Figure 4B). Consistent with our previously published results, the distribution of total PEC effector CD8+ subpopulations shifts in IL-12-deficient mice such that the KLRG1- fractions FI and FII increase in frequency, while the KLRG1+ fractions FIII and FIV become severely diminished in frequency (Figure 4C, *left column*). Notably, the vast majority of *Il12a* ^−/−^ tgd057-specific CTLs were FI and FII (Figure 4C, *right column*), mirroring the shift among total PEC CTL populations in the absence of IL-12. This effect was also observed in splenic tgd057-specific CD8+ T cells (Figure 4D). Therefore among both total and tgd057-specific CTLs, pro-inflammatory IL-12 signaling clearly induces the differentiation of the KLRG1+ effector subpopulations FIII and FIV at the expense of the KLRG1- memory precursor subsets FI and FII.

**Figure 5:**
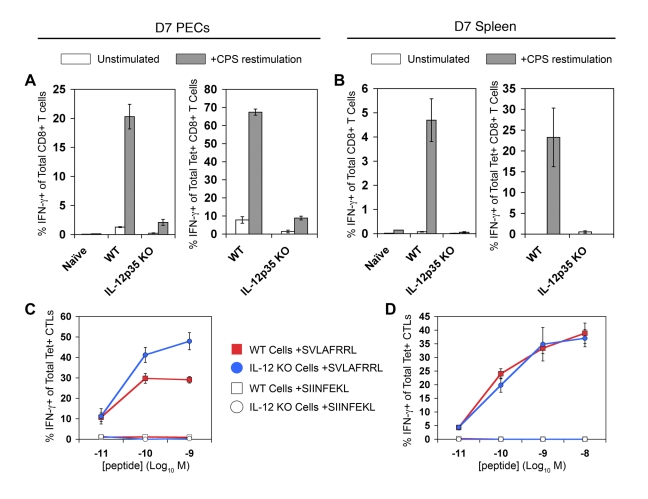
Tgd057-specific CTLs primed in *Il12a* ^−/−^ mice do not produce IFN-γ in response to parasite restimulation. Day 7 CPS-primed PECs and spleens were restimulated ex vivo to assay IFN-γ production. *A* and *B*) PECs (*A*) and splenocytes (*B*) were restimulated with CPS parasites ex vivo. The frequency of IFN-γ+ cells was determined among total CD8α+ CD3ε+ cells of WT naïve, WT primed, and *Il12a* ^−/−^ primed PECs and spleens, *left panels*. The frequency of IFN-γ+ cells was determined among total K^b^/SVLAFRRL+ CD8α+ CD3ε+ cells of WT naïve, WT primed, and *Il12a* ^−/−^ primed PECs and spleens, *right panels*. *C* and *D*) PECs (*C*) and splenocytes (*D*) were directly restimulated with a range of cognate and non-cognate peptide concentrations ex vivo. The frequency of IFN-γ+ cells was determined among total K^b^/SVLAFRRL+ CD8α+ CD3ε+ cells of WT primed, and *Il12a* ^−/−^ primed cells. All values are mean ± SEM of three mice. Data are representative of three independent experiments.

As predicted by our previously published results, IFN-γ-producing effector CD8+ T cells fail to differentiate in *Il12a* ^−/−^ PECs and splenocytes, when assayed by ex vivo restimulation with live parasites (Figure 5A and 5B, *left panel* *s*). When gated on tetramer+ CTLs, it is also clear that Ag-specific CTLs primed in the absence of IL-12 do not produce IFN-γ in response to parasite restimulation (Figure 5A and 5B, *right panels*). Although the functional defect in CD8+ T cell IFN-γ production due to in vivo IL-12 deficiency is striking, it is still not clear whether this defect is a result of aberrant in vivo differentiation or the lack of IL-12 signaling during ex vivo restimulation. Therefore, day 8 CPS-primed WT and *Il12b*(IL-12p40)*^−/−^* cells were restimulated under different culture conditions to elucidate the individual contributions of parasite antigens and IL-12 signaling for CD8+ T cell IFN-γ production ex vivo (Figure S2). Neutralizing IL-12 signaling during restimulation with anti-IL-12p40 Abs reduced IFN-γ production in WT CTLs but had no affect on *Il12b^−/−^* CTLs (*green vs. yellow bars*). The addition of rIL-12p70 alone stimulated a low percentage of WT CTLs to produce IFN-γ, however, *Il12b^−/−^* CTLs were insensitive to exogenous IL-12 signaling (*blue vs. purple bars*). The addition of CPS parasites plus rIL-12p70 into the restimulation culture not only induced WT CTLs to make more IFN-γ, but also allowed for *Il12b^−/−^* CTLs to produce IFN-γ (*red bars*). However, the frequency of IFN-γ+ cells among *Il12b^−/−^* CTLs was much lower relative to the WT CTLs in both the PECs and the spleen. Therefore, exogenous IL-12 signaling can synergize with antigenic stimulation to induce IFN-γ production in *Il12b^−/−^* CTLs, however, it cannot fully recover to WT levels, due to the prominent defect in differentiation that occurs with IL-12 deficiency.

Because parasite restimulation induces both Ag-dependent T cell activation and cytokine-induced bystander activation, WT and *Il12a* ^−/−^ cells were restimulated with varying concentrations of cognate peptide to determine the ability of tgd057-specific CTLs to respond to TCR ligation only. Surprisingly, both WT and *Il12a* ^−/−^ tgd057-specific CTLs mounted IFN-γ responses to increasing concentrations of cognate peptides (Figure 5C and 5D). The normal IFN-γ responses of *Il12a* ^−/−^ CTLs during peptide restimulation indicate that strong TCR signaling can also overcome the functional defect observed during CPS restimulation. In cultures stimulated with live parasites, it may be that only very low concentrations of SVLAFRRL-peptide are available on antigen presenting cells, thus explaining the strict requirement for pro-inflammatory IL-12 signaling. The difference between microbial and peptide restimulation is an important one and we believe that the IFN-γ responses to whole parasite restimulation better reflects the physiological effector function of CTLs in vivo, as it is well documented that IL-12-deficient mice rapidly succumb to toxoplasmosis due to the lack of T cell IFN-γ production [20],[31].

### Effector memory CTL differentiation in IL-12-deficient immune mice

**Figure 6:**
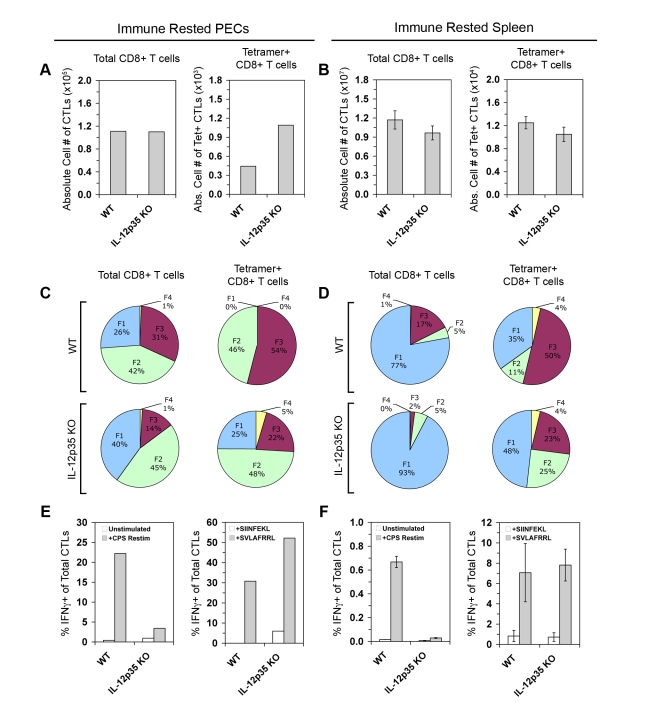
IL-12 is required to differentiate IFN-γ-producing effector memory CTLs. PECs and spleens of immune rested (day 28 post-CPS vaccination) WT and *Il12a* ^−/−^ mice were analyzed for numbers, phenotype, and effector function in memory CTLs. *A* and *B*) Total CD8α+ TCRβ+ cells and CD44^hi^ K^b^/SVLAFRRL+ CD8+ T cells were enumerated in PECs (*A*) and spleens (*B*) of WT and *Il12a* ^−/−^ immune mice. *C* and *D*) Subpopulation distributions were shown in total CD8α+ cells and K^b^/SVLAFRRL+ CD8α+ cells in PECs (*C*) and spleens (*D*) of WT and *Il12a* ^−/−^ immune mice. *E* and *F, left panels*) WT and *Il12a* ^−/−^ immune CD8α+ T cells among PECs (*E*) and from the spleen (*F*) were analyzed for IFN-γ positivity after restimulation with CPS parasites in vitro. *E* and *F, right panels*) WT and *Il12a* ^−/−^ immune K^b^/SVLAFRRL+ CD8α+ cells among PECs (*E*) and from the spleen (*F*) were analyzed for IFN-γ positivity after restimulation with either non-cognate (SIINFEKL) or cognate peptides (SVLAFRRL) in vitro. PEC samples were pools of four mice. Bar graphs are shown as mean ± SEM of four mice. Pie charts are shown as mean values for the spleen samples. One of two representative experiments is shown.

In the murine model of listeriosis, Pearce et al demonstrated that IL-12 deficiency enhances bacterial immunity through the increased generation of memory precursor CTLs (specific for the model antigen OVA) [27]. However, it is not known whether IL-12 has a similar role in memory CTL responses to parasites. Given the differential requirements of IL-12 for effector CTL subset differentiation in the primary response (Figure 4C and 4D), it may be that the enhancement of CD8 memory in IL-12-deficient conditions will be evident as an increase in the frequency of one or more subpopulation(s). By tracking the development and effector function of tgd057-specific CTLs in WT and IL-12-deficient immune mice, we can for the first time elucidate the role of IL-12 in effector/memory differentiation of a natural CTL population induced by parasite vaccination. In agreement with studies in bacterial and viral systems [27],[41], the numbers of PEC tgd057-specific CTLs were greater in *Il12a* ^−/−^ immune-rested mice (day 30 post-vaccination) compared to WT immune-rested mice, despite similar numbers of total CD8+ T cells (Figure 6A). In the spleens of *Il12a* ^−/−^ immune mice, however, the numbers of total CD8+ T cells and tgd057-specific CTLs were reduced on average, but not significantly different (Figure 6B). Tgd057-specific CD8+ T cells of WT immune-rested mice remained heterogeneous, with FI, FII, and FIII subsets present in PECs and the spleen (Figure 6C and 6D, *upper row*). The KLRG1+ FIII subset represented a major subpopulation of tgd057-specific memory CTLs. This is surprising since KLRG1 positivity is a marker of replicative senescence [24]. Despite the apparent longevity of some KLRG1+ cells, the effect of IL-12 deficiency persisted in memory tgd057-specific CTLs, where the balance of KLRG1 negative and positive subpopulations continued to favor the KLRG1- subsets in the absence of IL-12 (Figure 6C and 6D). Strikingly, the FI subpopulation remained a major constituent of the PEC tgd057-specific CTLs of *Il12a* ^−/−^ immune mice, whereas this subset was virtually absent from the PEC tgd057-specific CTLs of WT immune mice (Figure 6C), an effect that was not reflected in the total WT and *Il12a* ^−/−^ PEC populations. Hence, IL-12 deficiency during the priming of tgd057-specific CTLs may indeed result in a greater proportion of KLRG1- memory CTLs.

The persistent changes in subpopulation distributions observed in *Il12a* ^−/−^ immune rested mice suggested that there may also be long-lasting functional defects in T_EM_ IFN-γ responses. After ex vivo parasite restimulation, *Il12a* ^−/−^ memory CTLs mounted a weak recall response, whereas WT memory CTLs were efficient IFN-γ producers, especially in peripheral tissue (Figure 6E and 6F, *left panel* *s*). As previously observed in the primary response, tetramer+ CD8+ T cells from *Il12a* ^−/−^ immune mice were competent IFN-γ producers after cognate peptide restimulation (Figure 6E and 6F, *right panel* *s*). Therefore the enrichment of KLRG1- memory CTL precursors in IL-12 deficient conditions may be carried over from the primary response into the memory phase, where the KLRG1- subpopulations FI and FII account for nearly 75% of all tgd057-specific memory CTLs compared to 46% in WT immune mice. However, IL-12 remains a critical signal for IFN-γ production to parasite restimulation in primary-effector and effector-memory CD8+ T cells.

### IL-12 signaling during primary CTL activation is required for immediate recall responses but not for secondary CTL activation in immune mice

**Figure 7:**
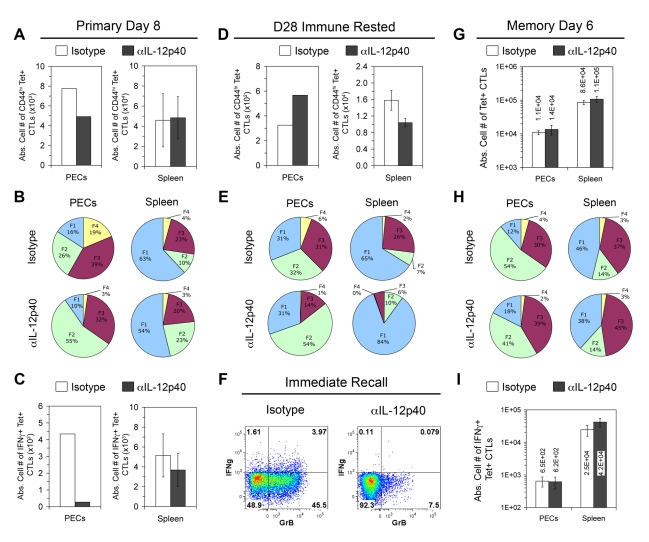
In vivo IL-12 neutralization during vaccination generates defective effector memory but not central memory CTLs. All mice were treated with Abs against IL-12p40 or an isotype control during CPS vaccination. *A–C*) To confirm the effect of IL-12 neutralization, PECs and spleens were analyzed for the activation of tgd057-specific CTLs on day 8 post-vaccination in IL-12p40 treated (Rx) or isotype Rx mice. CD44^hi^ K^b^/SVLAFRRL+ CD8α+ TCRβ+ cells were enumerated in *A*, and the subpopulation distributions of K^b^/SVLAFRRL+ CD8α+ cells were given in *B*. Samples were restimulated with CPS parasites and the frequency of IFN-γ+ cells in total K^b^/SVLAFRRL+ CD8α+ cells is shown in *C*. *D* and *E*) Resting numbers and subpopulation distributions of tgd057-specific CTLs were analyzed in IL-12p40 Rx or isotype Rx immune rested mice as in *A* and *B*. *F*) Immune rested mice were in vivo rechallenged with 2×10^6^ CPS parasites for 12 hrs. PECs from IL-12p40 Rx or isotype Rx rechallenged immune mice were harvested and ex vivo stained for IFN-γ and granzyme B expression in total CD8α+ TCRβ+ cells. FACS plots are from one of three mice per group. *G–I*) Immune rested mice were in vivo rechallenged with 10^7^ CPS parasites. On day 6 post-rechallenge, PECs and spleens were analyzed for the secondary activation of tgd057-specific CTLs. *G* and *H*) Resting numbers and subpopulation distributions of tgd057-specific CTLs were analyzed in IL-12p40 Rx or isotype Rx immune rested mice as in *A* and *B*. *I*) IFN-γ production was assessed in K^b^/SVLAFRRL+ CD8α+ cells after CPS restimulation. PEC samples were pools of 3–4 mice. For unpooled samples, bar graphs are shown as mean ± SEM of 3–4 mice and pie charts are mean values of 3–4 mice. One of two representative experiments is shown.

Although IL-12 is required for primary-effector and effector-memory differentiation, it is not yet clear how it may affect central memory CD8+ T cells. In primary anti-*Toxoplasma* effector CTLs, we have previously shown that the FII, FIII, and FIV subpopulations were equally responsive to ex vivo IL-12 signaling via pSTAT4 activation [22]. However, the FI subset was unresponsive to IL-12 signaling, suggesting that the detrimental effects of IL-12 in short-lived effector cell differentiation might spare the FI subset during the primary response. Since these T_CM_-phenotype FI cells are enriched in *Il12a* ^−/−^ immune mice, T_CM_ function might be normal or even enhanced in memory CTLs differentiated in an IL-12 deficient environment. To test this hypothesis, we transiently neutralized IL-12 in vivo during primary vaccination and queried the ability of the subsequent IL-12-sufficient immune mice to mount an immediate recall response in vivo and to generate effector memory cells 6 days after secondary challenge. Similar to the effects of genetic deletion of IL-12, mice that received anti-IL-12p40 Abs generated reduced numbers of PEC tgd057-specific CTLs on day 8 post-vaccination, which were diminished in KLRG1+ subsets and refractory to CPS restimulation (Figure 7A–7C). The effect of IL-12 neutralization was less severe in the spleen, which may be due to the i.p. route of Ab administration. On day 28 after the initial vaccination, the numbers of tgd057-specific CTLs were increased in PECs but decreased in the spleen of anti-IL-12 treated mice (Figure 7D), similar to *Il12a* ^−/−^ immune rested mice. Furthermore, KLRG1+ subsets, especially FIII, were under-represented in PEC and splenic tgd057-specific CTLs compared to control mice (Figure 7E). To test if effector memory CTLs from IL-12-neutralized immune mice could mount an immediate recall response in IL-12 replete conditions, mice were rechallenged with CPS parasites in vivo. PECs were harvested 12 hours later and directly stained for effector molecule expression. As shown in Figure 7F, recalled control effector memory CTLs expressed granzyme B and IFN-γ whereas those from IL-12 neutralized mice did not. Therefore, the effect of IL-12 deficiency during priming is imprinted on T_EM_ and cannot be ameliorated by the IL-12 sufficient conditions prevailing during the immediate recall response. As a measure of T_CM_ function, which is distinguished by high proliferative potential, IL-12-neutralized immune mice were rechallenged with a high dose of CPS parasites and assayed for secondary CTL activation 6 days later. Upon secondary challenge, the numbers of tgd057-specific CTLs, effector subset distribution, and effector function normalized between control and IL-12-neutralized groups (Figure 7G–7I). Taken together, these data suggest that central memory CD8+ T cells develop normally in the absence of IL-12 during the initial priming events.

### IL-12 negatively regulates memory marker-expression among tgd057-specific CTLs

**Figure 8:**
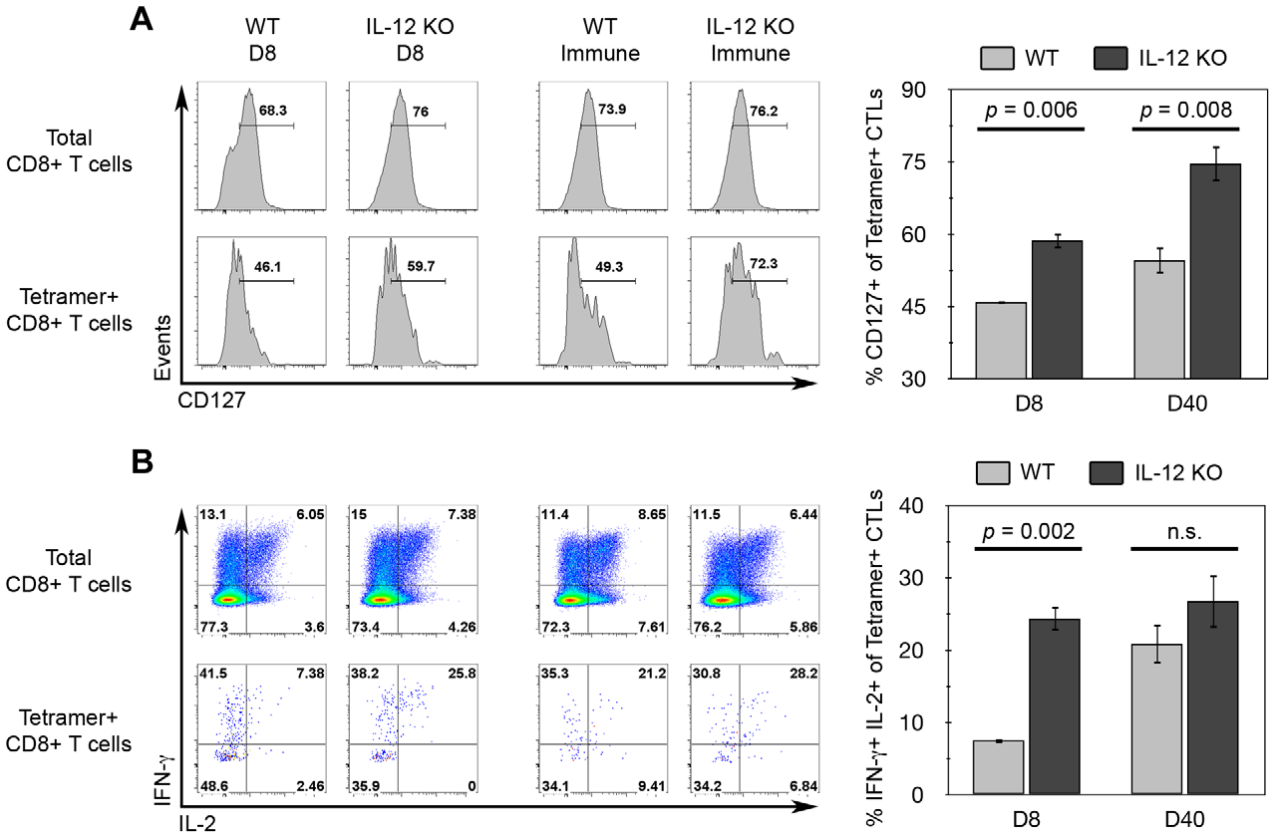
IL-12 inhibits CD127 and IFN-γ/IL-2 expression among tgd057-specific CTLs. WT and *Il12b^−/−^* mice were vaccinated with CPS parasites and spleens were harvested on day 8 (D8) and day 40 (D40, Immune) post-vaccination and analyzed by flow cytometry for memory markers expression among tgd057-specific CTLs. *A*) Ex vivo analysis of CD127 expression. *Left panel*, histograms of CD127 expression among either total CD8α+ TCRβ+ cells (total CD8+ T cells) or CD44^hi^ K^b^/SVLAFRRL+ CD8α+ TCRβ+ cells (Tetramer+ CD8+ T cells) from each group. *Right panel*, mean values of the frequency of CD127+ cells gated on Tetramer+ CD8+ T cells. *B*) IFN-γ and IL-2 expression after ex vivo restimulation with PMA and ionomycin for 4 hours. *Left panel*, IFN-γ and IL-2 co-expression profiles among either total CD8+ T cells or Tetramer+ CD8+ T cells from each group. *Right panel*, mean values of the frequency of IFN-γ and IL-2 double-positivity gated on Tetramer+ CD8+ T cells. Bar graphs are shown as mean ± SEM of four mice. n.s., not significant.

It has become clear that IL-12 deficiency results in the loss of KLRG1+ effector CTLs and hinders the ability of primary and memory cells to respond to parasite rechallenge. While CD8+ T cell numbers and function rely on IL-12, it is not yet clear if there is a qualitative change among memory precursor cells differentiated in the absence of IL-12 during *T. gondii* infection. Therefore, we probed for qualitative changes in memory phenotype between WT and *Il12b^−/−^* CD8+ T cells during primary (day 8) and memory (day 40) phases. CD127 (IL-7Rα) is a memory marker known to be selectively expressed on memory precursor cells [42], which is necessary for their survival [43]. As shown in Figure 8A, there was a greater frequency of splenic tgd057-specific memory precursor cells expressing CD127 from IL-12-deficient mice on day 8 post-vaccination. Moreover, the enhanced CD127 positivity persisted among splenic memory tgd057-specific CTLs on day 40 post-vaccination. As a second measure of memory quality, IL-2 expression, a characteristic of memory-lineage CD8+ T cells [42], was also analyzed. IL-2 positivity has been used in conjunction with TNFα and IFN-γ positivity to define “multifunctional” T_H_1 cells that are protective against *Leishmania major* [44], and similarly with CD8+ T cells, the presence of multifunctional CTLs correlates with vaccine efficacy [45]. To measure IL-2 expression via intracellular staining we restimulated splenocytes with the polyclonal T cell activators PMA and ionomycin, and therefore, memory-lineage cells will be both IFN-γ+ and IL-2+. Like CD127, the frequency of IFN-γ+ IL-2+ cells among splenic tgd057-specific CTLs was significantly increased in IL-12-deficient mice on day 8 (Figure 8B). However, there was no significant difference IFN-γ/IL-2-positivity between WT and *Il12b^−/−^* memory tgd057-specific CTLs, which is probably due to the ability of all memory CD8+ T cells to produce IL-2. Therefore, there is a qualitative shift among primary tgd057-specific CTLs whereby this population appears more memory-like in the absence of IL-12. Furthermore, the enhanced CD127 positivity among IL-12-deficient CTLs is retained from the primary phase to the memory phase, showing that the effects of IL-12 deficiency can fixed as memory CTLs progressively differentiate over time.

### Central memory CTLs differentiated in the absence of IL-12 are superior generators of secondary effector cells

**Figure 9:**
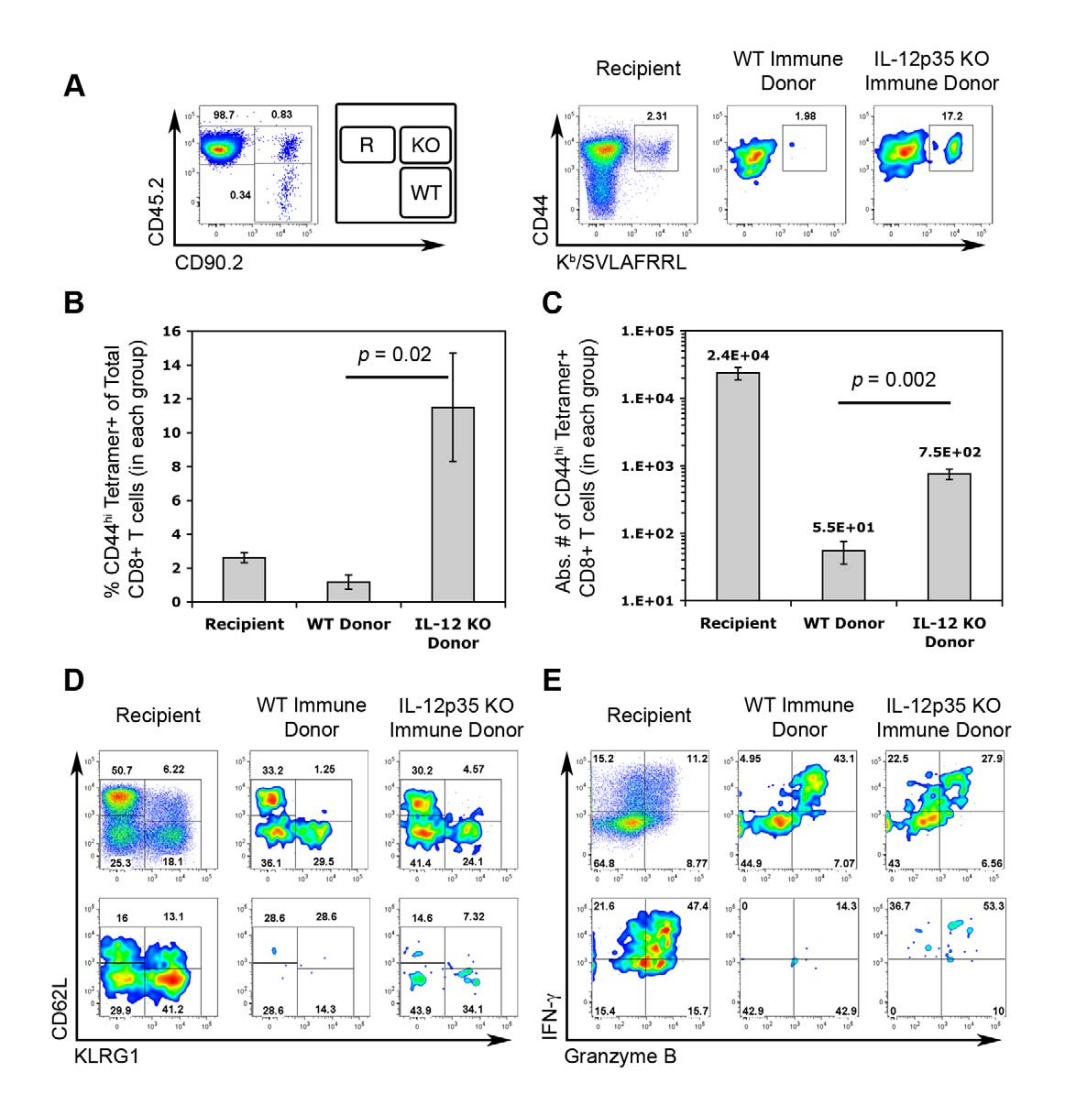
Central memory CTLs from *Il12a* ^−/−^ mice are functionally competent. CD44^hi^ FI central memory CD8α+ cells from the spleens of WT and *Il12a* ^−/−^ immune mice were FACS-sorted and then co-transferred into WT naïve mice. We calculated to have transferred ∼1800 FI tgd057-specific memory precursors of each genotype into each recipient mouse. One day post-transfer, chimeric mice were challenged with 3×10^6^ CPS. On day 5.5 post-challenge, PECs were analyzed for the generation tgd057-specific CTLs, subpopulation distributions, and effector function. *A, left panel*) Donor and recipient cells were distinguished based on the expression of congenic markers. Recipient mice were CD45.2+ and CD90.2-, cells of WT FI T_CM_ origin were CD45.2- and CD90.2+, and cells of *Il12a* ^−/−^ FI T_CM_ origin were CD45.2+ and CD90.2+. The FACS plot shows the relative frequencies of recipient and donor-derived CD8α+ TCRβ+ cells among PECs on day 5.5 post-challenge. *A, right panel*) The frequency of CD44^hi^ K^b^/SVLAFRRL+ CD8α+ T cells among total CD8+ T cells is shown for each gated population in the left panel. *B* and *C*) The frequency (*B*) and absolute cell numbers (*C*) of CD44^hi^ K^b^/SVLAFRRL+ CD8α+ T cells among recipient and donor groups. Bar graphs represent mean values ± SEM of 4 chimeric mice. *D*) Subpopulation distributions of total CD8+ T cells (*upper row*) and K^b^/SVLAFRRL+ CD8α+ T cells (*lower row*) among recipient and donor groups. *E*) Granzyme B and IFN-γ expression in total CD8+ T cells (*upper row*) and K^b^/SVLAFRRL+ CD8α+ cells (*lower row*) among recipient and donor groups after in vitro CPS restimulation. All FACS plots are from one representative chimeric mouse. One of two independent experiments is shown.

Transient IL-12-neutralization during *T. gondii* infection suggested that T_CM_ development does not require IL-12. However, due to the limitations of our experimental approach, it is not yet certain whether the complete absence of IL-12 will alter the functional quality of T_CM_. To more precisely assay the functional capabilities of central memory CTLs differentiated under IL-12-deficient conditions, CD44^hi^ FI CD8+ splenocytes from WT and *Il12a* ^−/−^ immune mice were sorted and co-transferred into naïve recipient mice. Given the increased precursor frequency of FI+ tgd057-specific memory CTLs in *Il12a* ^−/−^ immune mice (Figure 5C), donor FI+ CTLs were normalized such that the same number of predicted tgd057-specific cells of each genotype were transferred. Recipient, WT immune donor, and *Il12a* ^−/−^ immune donor CTLs were distinguished based on the expression of differential congenic markers (Figure 9A). Chimeric mice were then challenged with CPS parasites and assayed for CTL activation and differentiation 5.5 days later. Despite having transferred the same number of tgd057-specific F1 cells (but 58% fewer total FI CD8+ T cells) from immune *Il12a* ^−/−^ mice, secondary tgd057-specific CTLs derived from *Il12a* ^−/−^ T_CM_ were nearly 10-fold more frequent than those derived from WT T_CM_ among PECs on day 5.5 post-challenge (Figure 9A and 9B). Although PEC tgd057-specific CTLs of donor-origin did not accumulate in great numbers compared to the recipient's response, secondary tgd057-specific CTLs derived from *Il12a* ^−/−^T_CM_ were present in significantly greater cell numbers compared to those derived from WT T_CM_ (Figure 9C). Ex vivo phenotypic analysis of donor CTL populations revealed that FI T_CM_ generated phenotypically heterogeneous secondary effector CTLs regardless of their origin (Figure 9D). Upon restimulation of PECs from chimeric mice, granzyme B+ and IFN-γ-producing effector memory cells were evident in CTL populations originating from both WT and *Il12a* ^−/−^ T_CM_ donor cells (Figure 9E). Consistent with our data shown in Figure 7, it is clear that the development of central memory FI CTLs does not require IL-12. Remarkably, the resultant central memory CTLs differentiated under IL-12-deficient conditions and recalled in an IL-12 sufficient environment have a qualitative advantage in generating a functionally diverse population of secondary effector CTLs. Taken together with the increased representation of F1 memory CTLs observed after priming in the absence of IL-12 (Figure 6), the remarkable enhancement in the apparent fitness of T_CM_ formed in IL-12 deficient hosts indicates that IL-12 negatively impacts the central memory compartment both quantitatively and qualitatively.

## Discussion

Using caged MHC class I tetramer screening, we have discovered a new H-2K^b^-restricted CTL epitope SVLAFRRL, derived from the *T. gondii* protein tgd057. Tgd057 joins a growing list of newly identified CTL-targeted *Toxoplasma* antigens, including GRA6, GRA4, and ROP7. As the CTL epitopes defined for the previously reported three antigens are all H-2L^d^-restricted, this study is the first to characterize the CTL response to a natural *Toxoplasma* antigen in C57BL/6 mice. Because the C57BL/6 mouse strain is the most commonly used genetic background for infectious disease and basic immunology research [19], our discovery contributes an important tool for future research on CD8+ T cell responses during toxoplasmosis. Tgd057 was originally characterized as a highly abundant expressed sequence tag (EST) in *T. gondii* tachyzoites that is completely unique to this parasite and homologues are not found in other Apicomplexan organisms [36]. Notably, the nucleotide sequence of *tgd057* is completely identical amongst Type I, II and III strains of *T. gondii* (www.toxodb.org/toxo/). Using purified polyclonal anti-tgd057 Abs, we analyzed the localization of tgd057 within the parasite and found that it does not localize to the vacuolar lumen nor the parasitophorous vacuolar membrane, as expected of **-secreted proteins. Instead, tgd057 remained largely detergent insoluble, and appears to be associated with the parasite's cytoskeleton. Unlike GRA6 and GRA4, which localize to the intravacuolar network, and ROP7, which traffics to the parasitophorous vacuole membrane, tgd057 staining remained largely within the parasite. It is currently still unclear if tgd057 escapes the parasitophorous vacuole and how processing and presentation of tgd057 occurs. Nevertheless, our results indicate that *T. gondii* antigens which serve as CTL targets do not necessarily have to be secreted parasite proteins.

Tgd057-specific CTLs were activated during acute infection initiated with either *Toxoplasma* tachyzoites or bradyzoites, and after vaccination with live, irradiated parasites. Unlike ROP7- and GRA6-specific CTLs, whose numbers peak at 4–6 weeks post-infection with bradyzoites and correlate with the establishment of chronic infection [16],[17], tgd057-specific CTLs are induced at high frequency on day 7 during acute infection (Figure 2), suggesting that the tgd057 antigen is immediately available for processing in antigen presenting cells. Phenotypically, tgd057-specific effector CTLs were generally representative of the total CD8+ T cell population and contained higher frequencies of cells expressing granzyme B and IFN-γ compared to the polyclonal population. Similar to the immunodominant GRA6-specific CTLs mediating L^d^-restricted protective immunity in BALB/c mice which represent 20% of the total CTL population and a presumed majority of the IFN-γ producers [16], tgd057-specific CTLs probably also represent an immunodominant population representing approximately 10% of the total granzyme B+ effector CTL population on day 8 post-vaccination (as shown in Figure 3, where 2.4% (i.e. 53% of 4.53% tetramer+ CTLs are granzyme B+) of CD8+ T cells are both positive for tetramer-binding and granzyme B, whereas 29% of total CD8+ T cells have intracellular granzyme B). Tgd057-specific CTLs (obtained from ES-cloned mice following somatic cell nuclear transfer of individual nuclei from tgd057-tetramer+ CD8+ T cells into ES cells) can mediate significant protective immunity to lethal parasite challenge in adoptive transfer recipients (O. Kirak et al. *manuscript submitted*), suggesting that tgd057 is a protective CTL target antigen.

In light of previous reports demonstrating enhanced CTL memory responses in the absence of IL-12 during viral and bacterial infection [26],[27], we asked to what extent IL-12 controls memory CTL responses during parasite infection, given that IL-12 drives effector CTL differentiation in the primary response [22]. Using the memory CTL subpopulation distribution to gauge the relative frequencies of T_EM_ (CD62L^lo^ FII and FIII) and T_CM_ (CD62L^hi^ FI), we observed that IL-12 was required for the KLRG1+ mature FIII T_EM_ subset, but not for the KLRG1- FI (T_CM_) and FII (T_EM_) subsets. Furthermore, in mice transiently depleted of IL-12 during the primary response and in mice genetically deleted of IL-12, the resultant T_EM_ were incapable of mounting IFN-γ recall responses to parasite rechallenge. This indicates that IL-12 signaling during the primary CTL activation initiates a long-lasting effector program, including both IFN-γ production and KLRG1+ FIII subset differentiation. This program is imprinted on T_EM_ and cannot be recovered in the presence of IL-12 during the secondary response. The imprinting effect of IL-12, produced during primary CTL activation, on the effector competence of T_EM_ is consistent with previous in vitro experiments indicating that only a brief window of Ag/costimulation exposure is needed to initiate CTL clonal expansion [46] and that IL-12 stimulation for 30–60 hours in vitro is enough to program optimal effector function in Ag/B7-activated CTLs [47]. To our knowledge, our study is the first to clearly demonstrate effector imprinting by IL-12 in CD8+ effector memory T cells formed in response to microbial infection, an effect that may have been masked in viral and bacterial infection systems, due to the production of multiple and redundant “signal 3” cytokines and to the common practice of using peptide-pulsed rather than naturally-infected antigen presenting cells for restimulation.

The difference in the IFN-γ responses of IL-12-deficient CTLs when restimulated with infected cells versus cognate peptide remains somewhat puzzling. Because the tgd057-peptide dose responsiveness appears to be equivalent in CD8+ T cells developing in the presence or absence of IL-12, it seems unlikely that this difference is simply due to a lower level of antigenic peptide display on infected cells. Whereas the short-term peptide restimulation assay only queried the immediate capability of Ag-specific CTLs to make IFN-γ in response to TCR ligation, the precise contribution of TCR- and cytokine-driven IFN-γ production during the longer parasite restimulation assay is still unclear. Perhaps during the longer period required for the parasite restimulation, a second IL-12-dependent phase of stable IFN-γ production dominates.

It will be interesting to find out whether IL-27 receptor signaling functions along the same pathway as IL-12, given the striking defect in CD8+ T cell responses to *T. gondii* observed in IL-27R-deficient mice [48]. The role of IL-12 in driving CD8+ T cell effector and effector-memory differentiation which we describe here and elsewhere [22], is strikingly analogous to the established function of IL-12 in enhancing T_H_1 effector maturation [30],[49] and the recently delineated effect of IL-23 in T_H_17-mediated inflammation [50]. In all cases, innate signaling does not determine the cytokine phenotype of the T cell response *per se*. Rather, IL-12/-23's role is to ensure optimal expansion and to drive and maintain the differentiated state of effector cells, such that in their absence, effector cell development is stalled at an earlier differentiation stage.

Although IL-12 plays a positive and protective role in driving effector differentiation of primary-effector and effector-memory T cells responding to *T. gondii*, our results revealed a negative effect of IL-12 on the T_CM_ compartment. The results presented in Figures 6, 7, 8, and 9 clearly illustrated that IL-12 negative impacts quantitative and qualitative aspects of the central memory response. In *Il12a* ^−/−^ immune rested mice, the reduced frequency of the KLRG1+ FIII subset was accompanied by the emergence of FI as a prominent subset of peripheral memory CTLs (Figure 6B). The prominence of FI in *Il12a* ^−/−^ immune PECs represents a major qualitative shift in the resting pool of peripheral T_EM_ and T_CM_ and suggests that IL-12 drives effector memory differentiation at the cost of T_CM_ development. In addition to the increased incidence of FI cells, our adoptive transfer experiments (Figure 9) revealed a greater proliferative potential of FI-type cells formed in the absence of IL-12, manifesting as increased representation of their descendants during a secondary adaptive immune response. When responding under conditions with sufficient ambient IL-12, FI cells derived from IL-12-deficient mice showed no defect in their ability to generate a heterogenous population of competent effector cells.

Precisely how IL-12 favors the differentiation of effector CD8+ T cells at the expense of central memory T cell or their precursors remains unclear. The mechanism by which IL-12 regulates T_CM_ differentiation may be direct, assuming that at some point during their early development, T_CM_ precursors express IL-12 receptors. Low level or transient IL-12 signaling in T_CM_ precursors might shift the balance of transcription factors that regulate effector versus memory differentiation. Takemoto et al. demonstrated that IL-12 induces T-bet while repressing Eomes in WT effector CTLs [41]. Consequently, IL-12 deficiency during the initial priming may result in greater Eomes expression among central memory precursor CTLs. Eomes expression has been correlated with the enhanced responsiveness of memory CTLs to γ_c_-chain cytokines, as the IL-2/IL-15Rβ chain is a direct target of Eomes [51]. Hence, T_CM_ from IL-12-deficient mice may have enhanced homeostatic proliferation due to IL-15-induced self-renewal. In addition, the recently reported ability of IL-12 to enhance the stability of the immunological synapse [52] may result in a greater frequency of memory precursor T cells differentiating towards a terminally differentiated KLRG1+ effector fate.

Two recent reports highlight a new role for the transcriptional repressor Blimp-1 in the regulation of effector CD8+ T cell terminal differentiation during viral infection. It was demonstrated that CTL differentiation skewed toward T_CM_ development in Blimp-1-deficient mice [53],[54]. As a consequence, these CD8+ T cells lacked KLRG1 expression, effector molecule expression, and cytolytic function - an effect reminiscent of IL-12-deficient CTLs. Interestingly, primary Blimp-1^−/−^ anti-influenza CTLs expressed lower levels of T-bet transcripts and increased levels of Eomes and Bcl6 transcripts [54]. Given that IL-12 signaling also induces T-bet while repressing Eomes expression [41], it seems likely that IL-12 governs the expression of multiple transcription factors, including Blimp-1, for the coordinate induction of the T_C_1 effector program. However, what the current literature has lacked and what we now show is IL-12's influence extends beyond that of the terminal effector cells, reaching into the memory precursor subpopulation and negatively affecting memory function.

In summary, our results demonstrate that IL-12 signaling has an “antagonistically pleiotropic” effect on the development of effector memory and central memory compartments of the adaptive CD8+ T cell response to an intracellular pathogen. By efficiently promoting effector competence of CD8+ T cells to secrete cytokines and express cytolytic molecules, IL-12 protects the immunologically naive host against acute mortality caused by uncontrolled parasite growth. Nevertheless, this beneficial effect comes at a cost of limiting the reserve pool of memory T cells that can respond to subsequent re-infections. This delicate balance must be considered in the design and implementation of vaccination strategies involving the use of IL-12 and other innate cytokines as immunological adjuvants.

## Materials and Methods

### Mice and infection

Wild-type (WT) C57BL/6J, *Il12a* ^−/−^ (B6.129S1-*Il12a^tm1Jm^*/J), Thy1.1+ (B6.PL-*Thy1^a^*/CyJ), and CD45.1+ (B6.SJL-*Ptprc^a^ Pep^c^*/BoyJ) mice were purchased from The Jackson Laboratory (Bar Harbor, ME). *Il12b^−/−^* mice (C57BL/6NTac-[KO]IL12p40), housed at Taconic Farms (Germantown, NY), were a gift from Dragana Jankovic at the NIAID. All mice were housed under specific pathogen-free conditions at the University of Medicine and Dentistry of NJ (Newark, NJ) and were handled according to UMDNJ Institutional Animal Care and Use Committee guidelines. Sex- and age-matched mice were used in all experiments.

Fresh *T. gondii* tachyzoites were isolated from infected monolayers of human foreskin fibroblasts (HFFs) in culture. To vaccinate against *Toxoplasma*, 1×10^6^ live, mutant tachyzoites were i.p. injected into each mouse. These parasites (*cps1-1* or CPS) contain a disrupted carbamoyl phosphate synthetase (CPS) II gene, and are avirulent due to the inability to synthesize uracil [38]. In vitro, CPS cultures were grown in DMEM (Invitrogen, Carlsbad, CA) supplemented with 1% FBS, 1% penicillin/streptomycin (P/S), 1% L-glutamine, and 300 µM uracil. To ensure that the CPS parasites do not revert to the virulent parental strain (RH) in vivo, CPS parasites were irradiated (150 Gy) using a ^137^Cs source before injection. Natural *Toxoplasma* infection was mimicked by peroral administration of brain cysts containing *T. gondii* bradyzoites. On the day of infection, cysts were harvested from the brains of C57BL/6 mice chronically infected with the ME49 strain. Then, each mouse was gavaged with brain matter containing 20 cysts. GFP-RH parasites were obtained from American Type Culture Collection.

### Epitope prediction and peptide synthesis

Primary amino acid sequence data from selected ORFs was collected from the National Center for Biotechnology Information (www.ncbi.nlm.nih.gov/) and the *Toxoplasma gondii* Genome resource (www.toxodb.org/toxo/). The first and second screen consisted of 6 and 73 ORFs, respectively, from which 96 and 384 H-2K^b^-restricted 8-mer and H-2D^b^-restricted 9-mer peptides were predicted (Tables S1, S2 and S3) using a consensus epitope prediction program [32],[34]. The peptides identified for screening were synthesized by the biopolymers facility at MIT CCR (Cambridge, MA), whereas the conditional ligands were obtained by manual solid-phase peptide synthesis using commercially available Fmoc-protected amino acids as described earlier [34]. Quality assurance of the synthesis was provided by MALDI-TOF mass spectrometry, after which the crude peptides were lyophilized, taken up in DMSO (10 mg/mL), and kept at -20°C.

### Generation of MHC class I tetramers for epitope screening

Refolding of recombinantly expressed H-2K^b^ and H-2D^b^ complexes holding the SV9-P7* conditional ligand, biotinylation of the soluble MHC class I products and ensuing tetramerization was performed according to published protocols. Peptide exchange was performed in a 96-well plate on ice using longwave UV irradiation to replace the conditional ligand with cognate peptide as described [34].

### Generation of anti-tgd057 Abs

*Tgd057* cDNA (accession AY313956) was subcloned into the BamH1 site of pQE-60 bacterial expression vector containing a C-terminal 6xHis tag (QIAGEN). The tgd057 expression vector was transduced into *E. coli* XL1 blue supercompetent cells and tgd057 protein was induced by the addition of 1 mM Isopropyl-b-D-thiogalactoside. After 4 hours of induction recombinant tgd057 protein was harvested from the bacteria, purified by Ni-NTA affinity chromatography, and subsequently dialyzed to remove contaminating molecules, all according to the manufacturer's instructions (QIAGEN). The expression of tgd057 was detected by western blotting with anti-His mAbs and coomassie blue staining. Tgd057 antiserum was commercially generated by immunizing rabbits with 2 doses of 0.5 mg recombinant tgd057 over 2 months (Cocalico Biologicals, Reamstown, PA).

About 180 µg of purified recombinant tgd057 protein was further fractionated by SDS-PAGE. Protein was transferred to PVSF membrane, and then stained with Porcean S for 5 min followed by 2 washes in distilled water with gentle shaking. The tgd057 protein band, dominantly displayed at a MW of about 23 kDa, was cut from the membrane. The membrane strip was blotted with 200 µL anti-tgd057 serum in 20 mL PBS with 3% BSA and 0.05% Tween-20 overnight at 4 degrees C. Anti-tgd057 Abs were stripped by incubating the membrane strip with 5 mL of 100 mM glycine, pH 2.6 for 2 min. The specificity of the purified antibodies was confirmed by comparison with anti-His mAbs in detecting recombinant tgd057 using a western blot.

### Western blot

Two flasks of HFFs were inoculated with CPS parasites. Two days later, parasites were harvested just before HFF lysis. Infected HFFs were scraped and lysed by needle passage. After centrifugation, the resultant pellet from one flask (HFF+CPS) was resuspended in 100 µL lysis buffer (Modified RIPA Buffer: 50 mM Tris-HCl, pH 7.4 + 1% NP-40 + 0.25% Na-deoxycholate + 150 mM NaCl + 1 mM EDTA). The pellet from the other flask was vigorously resuspended and incubated in 100 µL 1% Triton X-100 in PBS on ice for 15 min. For the negative control, one flask of uninfected HFFs (HFF-CPS) was harvested and resuspended in 100 µL mRIPA buffer. All lysates were then centrifuged at 10,000 rcf for 15 min. The resultant supernatants were removed and transferred to a new tube. All pellets were resuspended in 100 µL modified RIPA buffer. 20 µL of each sample was separated on 4–12% Ready Gel precast gels (BIO-RAD, Hercules, CA) followed by transfer onto PVSF membrane. The membrane was blotted with anti-tgd057 rabbit Abs at a dilution of 1∶100. Following washing, the blot was incubated with goat anti-rabbit secondary Abs (Invitrogen) for 45 minutes at room temperature. Bands were visualized using LumiGOLD ECL detection kit (SignaGen Laboratories, Ijamsville, MD), followed by exposure to X-ray film.

### Immunofluorescence staining and microscopy

HFFs attached to microscope coverslips were infected with parasites in vitro for 30 min at 37°C. Extracellular parasites were subsequently washed out and the infected cells were incubated in DMEM culture medium + 1% FBS, 1% P/S at 37°C. After incubation, the HFFs were fixed (4% formaldehyde in PBS) for 15 min at RT. HFFs were then incubated in blocking buffer (PBS + 3% BSA, 0.2% saponin) for 60 min at RT while shaking. Primary Abs were diluted in blocking buffer and incubated with HFFs for 60 min at RT followed by extensive washing. HFFs were similarly stained with goat anti-rabbit IgG-AlexaFluor-568 secondary Abs (Invitrogen) for 45 min at RT. Coverslips were then mounted on microscope slides with ProLong Gold antifade reagent with DAPI (Invitrogen). Images were acquired on a Zeiss Axiovert 200 M fluorescent microscope and analyzed using AxioVision Rel 4.6 software.

### Tissue preparation and in vitro restimulation

Peritoneal exudates cells (PECs) were isolated by peritoneal lavage with RPMI 1640 (Invitrogen) supplemented with 2% FBS, 1% P/S, and 50 µM β-mercaptoethanol. Splenocytes were harvested by physical disruption of the spleen and passage through a 70 µM nylon mesh. The protocol for brain mononuclear cell isolation was kindly provided by Yasuhiro Suzuki. Briefly, brains were perfused with PBS, excised from the mouse and physically disrupted to free the mononuclear cells. Mononuclear cells were isolated by centrifugation in 35% Percoll (GE Healthcare, Piscataway, NJ) solution overlayed on pure FBS. Live cells were counted in a hemacytometer by trypan blue exclusion.

To restimulate the cells, samples were plated in 48-well TC-treated plates in RPMI Complete medium (RPMI 1640 + 10% FBS, 1% P/S, 50 µM β-mercaptoethanol) and inoculated with live CPS parasites (MOI = 0.1). CPS restimulated samples were incubated for 10 hours at 37°C with the addition of GolgiStop (BD Biosciences, San Jose, CA) for the last 4 hours. SVLAFRRL and SIINFEKL peptides were synthesized in house at the UMDNJ Molecular Resource Facility. For peptide restimulations, samples were plated in 96-well TC-treated plates in RPMI Complete medium. Unless otherwise noted, freshly thawed peptides were added at a final concentration of 10^−10^ M. Peptide restimulated samples were incubated for 6 hours at 37°C with the addition of GolgiStop for the last 4 hours. To induce detectable IL-2 expression by flow cytometry, samples were restimulated with PMA (50 ng/mL) and ionomycin (500 ng/mL) for 6 hours at 37°C with the addition of GolgiStop for the last 4 hours.

### Abs and tetramer staining

Cell surface Ab and tetramer staining was performed simultaneously in FACS Buffer (1XPBS + 1% BSA, 0.05% NaN_3_) for 1 hour on ice. The cells were then fixed in 100 µL BD Cytofix for 20 min on ice. For intracellular staining, Abs were diluted in BD Perm/Wash buffer and samples were stained for 30 min on ice. Fixed samples were resuspended in FACS Buffer and analyzed on either a BD FACS Calibur or BD LSRII flow cytometer. Flow data was analyzed using FlowJo (Tree Star, Ashland, OR). Mouse-specific Abs obtained from BD Biosciences: CD62L (MEL-14), CD45.2 (104). Mouse-specific Abs obtained from eBioscience (San Diego, CA): CD44 (IM7), IFN-γ (XMG1.2), CD3ε (145.2C11), CD8α (53-6.7), CD90.2 (53.2.1), CD127 (A7R34), TCRβ (H57-597), KLRG1 (2F1), IL-2 (JES6-5H4). Anti-human Granzyme B (GB11) was obtained from CALTAG Laboratories (Invitrogen, Carlsbad, CA).

### In vivo IL-12p40 neutralization

On day 0, each mouse was i.p. injected with 1 mg of either anti-IL-12p40 mAb (C17.8) or an isotype control (2A3) (BioXCell, West Lebanon, NH). After ∼4 hours mice were vaccinated with irradiated, CPS parasites. On day 3 post-vaccination mice were given another 1 mg dose of mAb.

### Sorting and adoptive transfer

CD8+ T cells were negatively selected from immune WT CD45.1 congenic and immune *Il12a* ^−/−^ spleens using magnetic microbeads (Miltenyi Biotec, Auburn, CA). WT and *Il12a* ^−/−^ CD8+ T cells were surface stained for CD44, CD8α, CD62L, and KLRG1. Stained samples were then sorted for CD44^hi^ FI+ (CD62L^hi^ KLRG1-) cells using a BD FACS Vantage cell sorter. In a separate pre-sorted sample, WT and *Il12a* ^−/−^ immune CD8+ T cells were stained for tetramer positivity within fractions in ex vivo immune splenocytes. The frequency of tetramer+ cells in FI immune CD8+ T cells was used to normalize the numbers of predicted tetramer+ cells in sorted samples. WT and *Il12a* ^−/−^ FI CD8+ T cells were mixed and i.v. injected (retro-orbital) into naïve WT CD90.1 congenic recipient mice.

## Supporting Information

Figure S1 Absolute cell numbers of tgd057-specific CTLs generated by either vaccination or natural infection. The absolute cell numbers of CD44^hi^ K^b^/SVLAFRRL+ CD8α+ TCRβ+ cells was calculated for samples corresponding to the groups in Figure 2. Values are mean ± SEM of three mice except for the Acute ME49 PECs, which is a pooled sample from three mice.
(0.15 MB TIF)

Figure S2 The effect of IL-12 addback on WT and IL-12-deficient CTLs during ex vivo CPS restimulation. PECs and spleens were harvested from day 8 (D8) CPS-primed WT and *Il12b* ^−/−^ mice. Spleens were also taken from naive WT mice to serve as negative controls. WT and *Il12b* ^−/−^ PECs and splenocytes were then restimulated under five different conditions for 10 hours. *Blue*, unstimulated cultures were left unadulterated. *Green*, “+CPS +Isotype” cultures contained CPS parasites at MOI of 0.1 and 20 µg of rat IgG2a isotype Ab. *Yellow*, “+CPS +anti-IL-12” cultures contained CPS parasites at MOI of 0.1 and 20 µg of anti-IL-12p40 mAb. *Red*, “+CPS +rIL-12” cultures contained CPS parasites at MOI of 0.1 and at three hours post-addition of parasites 1 ng rIL-12p70 was added. *Purple*, “rIL-12” cultures contained 1 ng rIL-12p70 added at three hours post-incubation. Values represent mean ± SEM of three mice.
(0.54 MB TIF)

Table S1 Manually curated list of sequences obtained from National Center for Biotechnology Information (www.ncbi.nlm.nih.gov/) and the *Toxoplasma gondii* Genome resource (www.toxodb.org/toxo/) for putative secreted proteins derived from *Toxoplasma gondii*. The predicted H-2K^b^-restricted octameric and H-2D^b^-restricted nonameric epitopes are highlighted in gray. The N-terminal residue of the individual epitopes is highlighted in black to discriminate overlapping sequences. Highlighted in red is the H-2K^b^-restricted epitope identified to give a CD8^+^ T cells response in C57BL/6 mice.
(0.16 MB DOC)

Table S2 The 48 and 192 highest scoring H-2K^b^-restricted octameric epitopes as assayed in screens 01 and 02, respectively. The epitopes were derived from putative secreted proteins of *Toxoplasma gondii*. Predictions were performed using a consensus epitope prediction algorithm. The program, including documentation for its execution and sample data, is freely available at http://jura.wi.mit.edu/bioc/grotenbreg. The epitope identified to give a CD8^+^ T cells response in B6 mice is highlighted in red.
(0.62 MB DOC)

Table S3 The 48 and 192 highest scoring H-2D^b^-restricted nonameric epitopes as assayed in screens 01 and 02, respectively. The epitopes were derived from putative secreted proteins of *Toxoplasma gondii*. Predictions were performed using a consensus epitope prediction algorithm. The program, including documentation for its execution and sample data, is freely available at http://jura.wi.mit.edu/bioc/grotenbreg.
(0.63 MB DOC)
