# Supplementary figures and images for: Differential Regulation of Effector- and Central-Memory Responses to Toxoplasma gondii Infection by IL-12 Revealed by Tracking of Tgd057-Specific CD8+ T Cells
Source: PLoS Pathog. 2010 Mar 19;6(3):e1000815. doi: 10.1371/journal.ppat.1000815 (PMC2841619; doi:10.1371/journal.ppat.1000815)

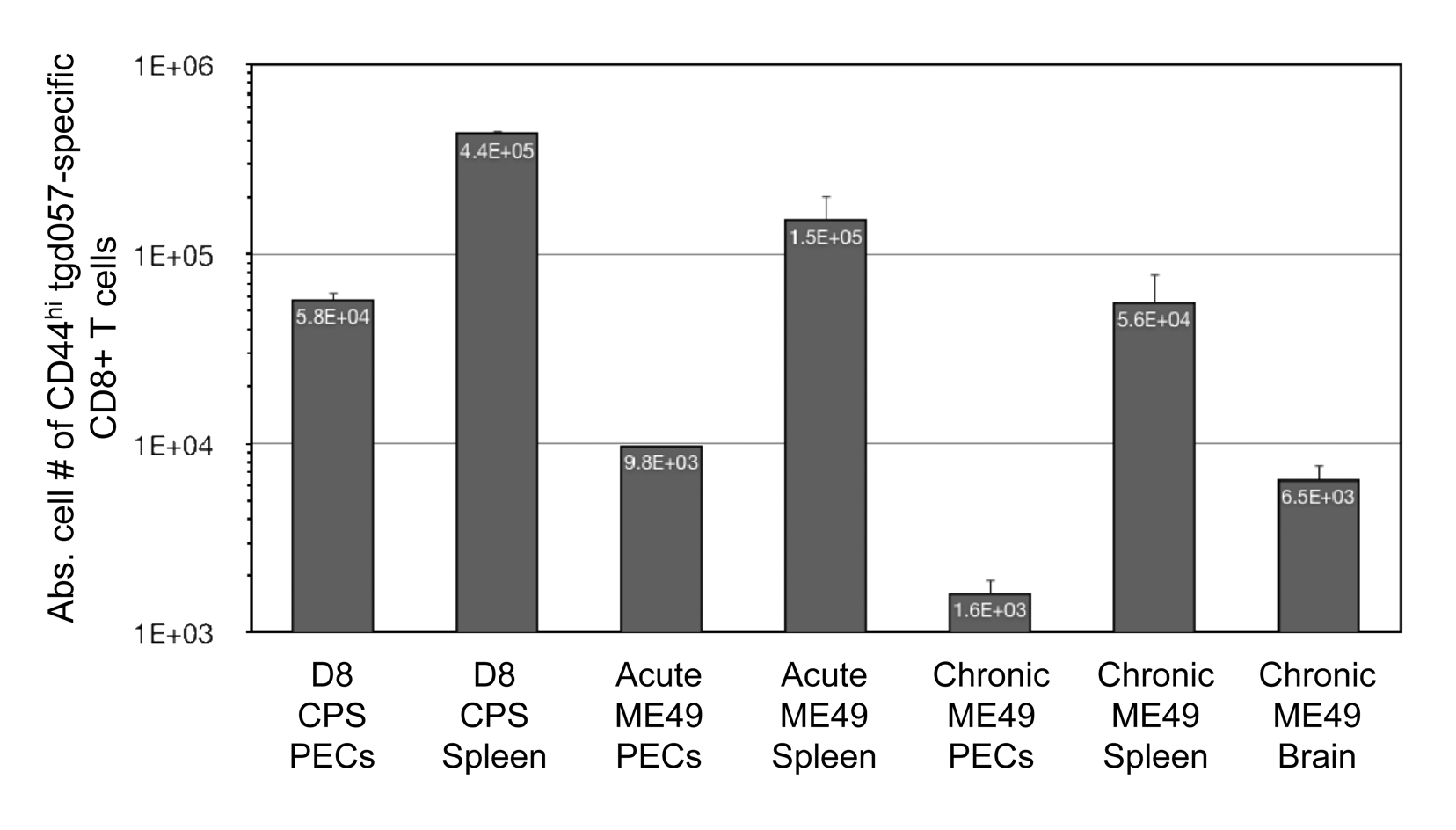

Supplement: Figure S1 — Absolute cell numbers of tgd057-specific CTLs generated by either vaccination or natural infection. The absolute cell numbers of CD44hi Kb/SVLAFRRL+ CD8α+ TCRβ+ cells was calculated for samples corresponding to the groups in Figure 2. Values are mean ± SEM of three mice except for the Acute ME49 PECs, which is a pooled sample from three mice. (0.15 MB TIF) [file ppat.1000815.s001.tif]

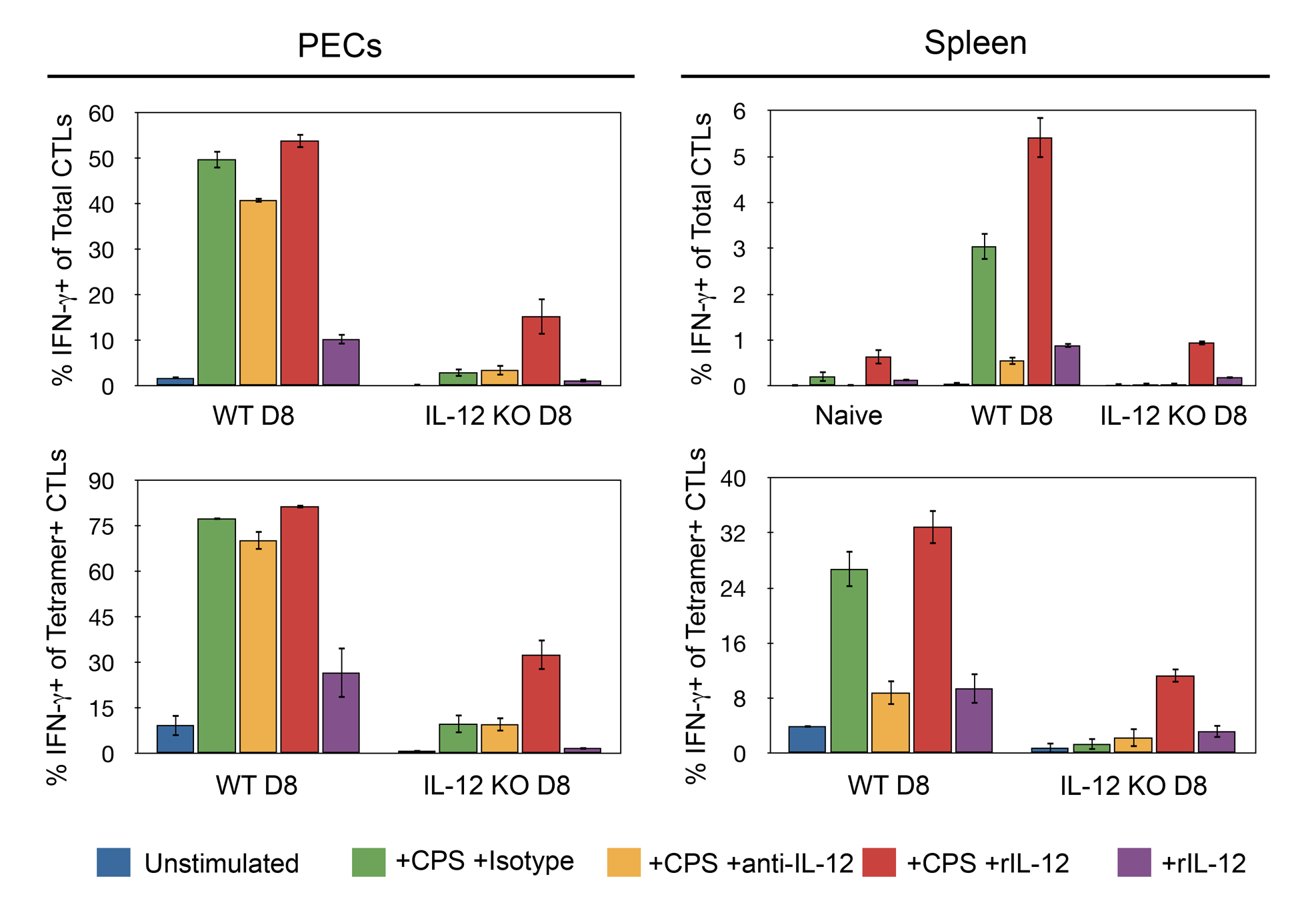

Supplement: Figure S2 — The effect of IL-12 addback on WT and IL-12-deficient CTLs during ex vivo CPS restimulation. PECs and spleens were harvested from day 8 (D8) CPS-primed WT and Il12b −/− mice. Spleens were also taken from naive WT mice to serve as negative controls. WT and Il12b −/− PECs and splenocytes were then restimulated under five different conditions for 10 hours. Blue, unstimulated cultures were left unadulterated. Green, “+CPS +Isotype” cultures contained CPS parasites at MOI of 0.1 and 20 µg of rat IgG2a isotype Ab. Yellow, “+CPS +anti-IL-12” cultures contained CPS parasites at MOI of 0.1 and 20 µg of anti-IL-12p40 mAb. Red, “+CPS +rIL-12” cultures contained CPS parasites at MOI of 0.1 and at three hours post-addition of parasites 1 ng rIL-12p70 was added. Purple, “rIL-12” cultures contained 1 ng rIL-12p70 added at three hours post-incubation. Values represent mean ± SEM of three mice. (0.54 MB TIF) [file ppat.1000815.s002.tif]
