# Supplementary material for: Differential Regulation of Effector- and Central-Memory Responses to Toxoplasma gondii Infection by IL-12 Revealed by Tracking of Tgd057-Specific CD8+ T Cells
Source: PLoS Pathog. 2010 Mar 19;6(3):e1000815. doi: 10.1371/journal.ppat.1000815 (PMC2841619; doi:10.1371/journal.ppat.1000815)
Supplement: Table S1 — Manually curated list of sequences obtained from National Center for Biotechnology Information (www.ncbi.nlm.nih.gov/) and the Toxoplasma gondii Genome resource (www.toxodb.org/toxo/) for putative secreted proteins derived from Toxoplasma gondii. The predicted H-2Kb-restricted octameric and H-2Db-restricted nonameric epitopes are highlighted in gray. The N-terminal residue of the individual epitopes is highlighted in black to discriminate overlapping sequences. Highlighted in red is the H-2Kb-restricted epitope identified to give a CD8+ T cells response in C57BL/6 mice. (0.16 MB DOC) [file ppat.1000815.s003.doc]

**Table I.** Manually curated list of sequences obtained from National Center for Biotechnology Information ([www.ncbi.nlm.nih.gov/](http://www.ncbi.nlm.nih.gov/)) and the *Toxoplasma gondii* Genome resource ([www.toxodb.org/toxo/](http://www.toxodb.org/toxo/)) for putative secreted proteins derived from *Toxoplasma gondii*. The predicted H-2K^b^–restricted octameric and H-2D^b^–restricted nonameric epitopes are highlighted in gray. The N-terminal residue of the individual epitopes is highlighted in black to discriminate overlapping sequences. Highlighted in red is the H-2K^b^–restricted epitope identified to give a CD8^+^ T cells response in C57BL/6 mice.

| **Screen 01** | | | |
| --- | --- | --- | --- |
| **Protein**  **number** | **Accession number** | **Common designation** | **Sequence and predicted epitopes** |
| **1** | P13403 | GRA1  (NCBI) | MVRVSAIVGAAASVFVCLSAGAYAAEGGDNQSSAVSDRASLFGLLSGGTGQGLGIGESVDLEMMGNTYRVERPTGNPDLLKIAIKASDGSYSEVGNVNVEEVIDTMKSMQRDEDIFLRALNKGETVEEAIEDVAQAEGLNSEQTLQLEDAVSAVASVVQDEMKVIDDVQQLEKDKQQLKDDIGFLTGERE |
| **2** | O00933 | GRA7  (NCBI) | MARHAIFSALCVLGLVAAALPQFATAATASDDELMSRIRNSDFFDGQAPVDSLRPTNAGVDSKGTDDHLTTSMDKASVESQLPRREPLETEPDEQEEVHFRKRGVRSDAEVTDDNIYEEHTDRKVVPRKSEGKRSFKDLLKKLALPAVGMGASYFAADRLVPELTEEQQRGDEPLTTGQNVGTVLGFAALAAAAAFLGMGLTRTYRHFSPRKNRSRQPALEQEVPESGEDGEDARQ |
| **3** | TgTwinScan_1315 | ROP2 | MENCASVRSSSCLIWLAAAFFVSALGHVQQGAGVVRPRHWQNSEAAVSVRPPGGASPRPFHSPIEPVAFIDGEHDEDKHEGSWLEQEAAEDVTPLPDSHTEAQTQSPSAFRRLTRRLRFWRRGVTGGSDAGEEAPQTPRPSLRTRILQYLRRVGTFFRRDIPAAALRFFRRFRRVRQPVFPPDEFPEDVDTNPIYFRGTDPGDVVIEELFNRIPQANVRTTSEYMQSAADSLVSTSLWNTGQPFRVESELGERPRTLVRGTVLGQEDPYAYLEATDQETGESFEVHVPYFTERPPSNAIKQMKEEVLRLRLLRGIKNQKQAKVHLRFIFPFDLVKDPQKKKMIRVRLDERDMWVLSRFFLYPRMQSNLQTFGEVLLSHSSTHKSLVHHARLQLTLQVIRLLASLHHYGLVHTYLRPVDIVLDQRGGVFLTGFEHLVRDGASAVSPIGRGFAPPETTAERMLPFGQHHPTLMTFAFDTWTLGLAIYWIWCADLPNTDDAALGGSEWIFRSCKNIPQPVRALLEGFLRY |
| **4** | CAA85377 | ROP2  (NCBI) | MENCASVRSSSCLIWLAAAFFVSALGHVQQGAGVVRPRHWQNSEAAVSVRPPGGASPRHFHSPIEPVAFIDGEHVEDKHGGSWLEQEAAEEVTPLLNSHTETPTQSPSAFRRLLRRLRFWRRGRTGGSDGGGEPPQTPRPSLPTRLFQHLRRAAAAIPAAASRFFRRFRRVQEPVFPPDEFPEDVDTNPMYFRGTDPGDVVIEELFNRIPETSVWNENERVLSNANHLVSTALWRNEQSFRVESELGERPRTLVRGPVLRDDGSYICLEATDQETGEPLEVHVPYFTERPPSNAIKQLSEQVLRLRLLRGIKNQRQAKAYLRFIFPIDLVKDPKKRKMIRVRLDERDMWVLSRFFLYPRMQSNLHILGDVLLSHSSTHKSLVHHARLQLTLQLIRLAASLQHYGLVHADFQVRNILLDQRGGVFLTGFEHLVRDGASAVSPIGRGFAPPETTAERMLPYRQHHPTLMTFPFDTWTLGLAIYWIWCADLPNTEDAELGGIEWIYRRCKNIPQPVRALLEGFLRYSKEDRLLPLQAMETSEYEQLRTELSAVLPLYQTDGEPA |
| **5** | CAA96467 | ROP4 | MGHPTSFGQPSCLVWLAAAFLVLGLCLVQQGAGRQRPHQWKSSEAALSVSPAGDIVDKYSRDSTEGENTVSEGEAEGSRGGSWLEQEGVELRSPSQDSQTGTSTASPTGFRRLLRRLRFWRRGSTRGSDDAAEVSRRTRVPLHTRLLQHLRRVARIIRHGVSAPLADCSGEFGKLRRNVHNPFSLKVSPDLETNSLYYRDKVPGQGIIQEILRQKPGIAHHPESFSVVAADERVSRTLWAEGGVVRVASELGQPGRVLVRGRRIGLFRPGMQFEATDQATGEPMTALVGHTVLEATARDVDSMRNEGLAVGLFQKVKNPYLANRYLRFLAPFDLVTIPGKPLVQKAKSRNEVGWVKNLLFLLPPTHVDMETFVDEIGRFPQEDRPLADAARLYLTVQAVRLVAHLQDEGVVHGKIMPDSFCLKREGGLYLRDFGSLVRAGAKVVVPAEYDEYTPPEGRAAARSRFGSGATTMTYAFDAWTLGSVIFLIWCSRAPDTKSGYEYSVEFFFSRCRRVPENVKLLVYKLINPSVEARLLALQAIETPEYREMEEQLSARSRLYSGDGTLTGGDDDMPPLET |
| **6** | AAO72426 | SAG1 | MSVSLHHFIISSGFLTSMFPKAVRRAVTAGVFAAPTLMSFLRCGAMASDPPLVANQVVTCPDKKSTAAVILTPTENHFTLKCPKTALTEPPTLAYSPNRQICPAGTTSSCTSKAVTLSSLIPEAEDSWWTGDSASLDTAGIKLTVPIEKFPVTTQTFVVGCIKGDDAQSCMVTVTVQARASSVVNNVARCSYGANSTLGPVKLSAEGPTTMTLVCGKDGVKVPQDNNQYCSGTTLTGCNEKSFKDILPKLSENPWQGNASSDNGATLTINKEAFPAESKSVIIGCTGGSPEKHHCTVQLEFAGAAGSAKSSAGTASHVSIFAMVTGLIGSIAACVA |
|  | | | |
| **Screen 02** | | | |
| **Protein**  **number** | **Accession number** | **Common designation** | **Sequence and predicted epitopes** |
| **1** | TgTwinScan_6462 | AMA1 | MGLVGVASLLVLVADCTIFASGLSSSTRSRESQTLSASTSGNPFQANVEMKTFMERFNLTHHHQSGIYVDLGQDKEVDGTLYREPAGLCPIWGKHIELQQPDRPPYRNNFLEDVPTEKEYKQSGNPLPGGFNLNFVTPSGQRISPFPMELLEKNSNIKASTDLGRCAEFAFKTVAMDKNNKATKYRYPFVYDSKKRLCHILYVSMQLMEGKKYCSVKGEPPDLTWYCFKPRKSVTENHHLIYGSAYVGENPDAFISKCPNQALRGYRFGVWKKGRCLDYTELTDTVIERVESKAQCWVKTFENDGVASDQPHTYPLTSQASWNDWWPLHQSDQPHSGGVGRNYGFYYVDTTGEGKCALSDQVPDCLVSDSAAVSYTAAGSLSEETPNFIIPSNPSVTPPTPETALQCTADKFPDSFGACDVQACGSNTALIAGLAVGGVLLLALLGGGCYFAKRLDRNKGVQAAHHEHEFQSDRGARKKRPSDLMQEAEPSFWDEAEENIEQDGETHVMVEGDY |
| **2** | TgTwinScan_7296 | GRA1 | MVRVSAIVGAAASVFVCLSAGAYAAEGGDNQSSAVSDRASLLGLLSGGTGQGLGIGESVELEMMGNTYRVERPTGNPDLLKIAIKTSDGSYSEVGNVNMEEVIDTMKSMQRDEEIFFRALNKGETVEEAIEDVAQAEGLNSEQTLQLEDAVSAVASVVQDEMNVIDDVQQLEKDKQQLKDDIGFLTGERE |
| **3** | TgTwinScan_5534 | GRA2 | MFAVKHCLLVVAVGALVNVSVRAAEFSGVVNQGPVDVPFSGKPLDERAVGGKGEHTPPLPDERQQEPEEPVSQRASRVAEQLFRKFLKFAENVGQHSEKAFKKAKVVAEKGFTAAKTHTVRGFKVAKEAAGRGMVTVGKKLANVESDRSTTTTQAPDSPNGLAETQAPVEPQQRAAHVPVPDFSQ |
| **4** | TgTwinScan_5513 | GRA3 | MSTALKRLIPFLVPFVVFLVAAALGGLAADQPGNHQALAEPVTGVGEAGVSPVNEAGESYSSATSGVQEATAPGAVLLEAIDAESDKVDNQAEGGERMKKVEEELSLLRRELYDRTDRPGLKRAVILSLGTSALIAGRMFSSTLRAAVPWYAVAFNAIVAAYYIRKVLTYRRRVMTKRQPFMSSVKNFFRRRPKDGGAGVDKASKK |
| **5** | TgTwinScan_4647 | GRA4 | MQGTWFSLFVVVMVSHLAFGGECSFGSYLADMAGLTGREDKEHHAAKKRYYHSMYGNKTPYPYADGQQGSPPPQGQLLIIQNPDGSFMIVDQQGVPQAPQAAGGPGSPMNGGYYMPAGVYTAQVVPGTPGHPVQAIPQQPLRTQATATYYHPAAAPPPGPSVFVFTPSSVQPGAEVTPGYSGLQLRQQSQYDYSYPGTTSTPTPPRPASYGYPAFPAFPRLPAFSDSVSVSTEDSGLTVVRDSSSSESTVTPADEAASESEEGDKTSRKSKVKKGVLTGLGVAATLAAAAAAAKAVKGFGGTRTSTAPAEAGKTELDDGYRPPPFNPRPSPYAELLKDLERMRKE |
| **6** | TgTwinScan_3556 | GRA5 | MASVKRVVVAVMIVNVLALIFVGVAGSTRDTGSGGDDSEGAWGGEQQQVQQHGQSEDRSLFERGRAAVTGHPVRTAVGLAAAVVAVVSLLRLLRRRRRRAIQEESKESATAEEEEVAEEE |
| **7** | Q27003 (NCBI) | GRA6 | MAHGGIHLRQKRNFCPVTVSTVAVVFVVFMGVLVNSLGGVAVAADSGGVKQTPSETGSSGGQQEAVGTTEDYVNSSAMGGGQGDSLAEDDTTSEAAEGDVDPFPVLANEGKSEARGPSLEERIEEQGTRRRYSSVQEPQAKVPSKRTQKRHRLIGAVVLAVSVAMLTAFFLRRTGRRSPQEPSGDGGGNDAGNNAGNGGNEGRGYGGRGEGGAEDDRRPLHPERVNVFDY |
| **8** | TgTwinScan_3779 | GRA7 | MARHAIFFALCVLGLVAAALPQFATAATASDDELMSRIRNSDFFDGQAPVDSLRPTNAGVDSKGTDDHLTTSMDKASVESQLPRREPLETEPDEQEEVHFRKRGVRSDAEVTDDNIYEEHTDRKVVPRKSEGKRSFKDLLKKLALPAVGMGASYFAADRILPELTEQQQTGEEPLTTGQNVSTVLGFAALAAAAAFLGMGLTRTYRHFSPRKNRSRQPALEQEVPESGKDGEDARQ |
| **9** | TgTwinScan_3039 | GRA8 | MALPLRVSATVFVVFAVFGVARAMNGPLSYHPSSYGASYPNPSNPLHGMPKPENPVRPPPPGFHPSVIPNPPYPLGTPAGMPQPEVPPLQHPPPTGSPPAAAPQPPYPVGTPGMPQPEIPPVHRPPPPGFRPEVAPVPPYPVGTPTGMPQPEIPAVHHPFPYVTTTTTAAPRVLVYKIPYGGAAPPRAPPVPPRMGPSDISTHVRGAIRRQPATATTTTTTRNVLLRTAILAAAAATLIALFRQRPLFTEGVRMFPDFQYRFTVQTTQ |
| **10** | TgTwinScan_1247 | M2AP | MKLAAVSSAAFAAAFFPAVVNARKVGNPAAQPSVLVNEPVALAQLSTFLELVEVPCNSVHVQGVMTPNQMVKVTGAGWDNGVLEFYVTRPTKTGGDTSRSHLASIMCYSKDIDGVPSDKAGKCFLKNFSGEDSSEIDEKEVSLPIKSHNDAFMFVCSSNDGSALQCDVFALDNTNSSDGWKVNTVDLGVSVSPDLAFGLTADGVKVKKLYASSGLTAINDDPSLGCKAPPHSPPAGEEPSLPSPENSGSATPAEESPSESESVPSLPLSQIPSEVPLSQESPVPPQDGENPDSPQNEDNSMQEVENSASQSDIEAQRQAENGRLPSDDEA |
| **11** | TgTwinScan_0297 | MIC1 | MGQALFLTVLLPVLFGVGPEAYGEASHSHSPASGRYIQQMLDQRCQEIAAELCQGGLRKMCVPSSRIVARNAVGITHQNTLEWRCFDTASLLESNQENNGVNCVDDCGHTIPCPGGVHRQNSNHATRHEILSKLVEEGVQRFCSPYQASANKYCNDKFPGTIARRSKGFGNNVEVAWRCYEKASLLYSVYAECASNCGTTWYCPGGRRGTSTELDKRHYTEEEGIRQAIGSVDSPCSEVEVCLPKDENPPVCLDESGQISRTGGGPPSQPPEMQQPADRSDERGGGKEQSPGGEAQPDHPTKGGNIDLPEKSTSPEKTPKTEIHGDSTKATLEEGQQLTLTFISTKLDVAVGSCHSLVANFLDGFLKFQTGSNSAFDVVEVEEPAGPAVLTIGLGHKGRLAVVLDYTRLNAALGSAAYVVEDSGCSSSEEVSFQGVGSGATLVVTTLGESPTAVSA |
| **12** | TgTwinScan_4394 | MIC10 | MALSSLNNIRPFSGLLGCGLLFGALVVVVACVFSVPVEAGVLRKVAGAGSLQASIGEHDFFNDYDQDEEYRKRQQELQNQSPEEVEEAKRKYHEELRRKAEEDAETKRKQEAVIQELKEVAKKRGLREAAEREEKRIDEQQANYEQRQQELRDMDSAMEERLMQQRKKDQEERELARKNSDKVMEELKEKLARRRKSM |
| **13** | TgTwinScan_3888 | MIC11 | MQLKKLSVVSITLLGLFKFVNGVSEGVVVPVRFGSETARADLLNQVSETVFSLVETTEDDKSAASIVRGAFRSALKEILVAVKGEVQQTCEELADLAARKIHEAEERGEGRLDKEEAVDDESDFSFLDSPKTSEASIRPHGFLQERKFVDTLKTLAKGALKTFVEPMKAALVDGIRPMLPKIKEVAIDVFRQACEHAEEKLQDA |
| **14** | TgTwinScan_3636 | MIC2 | MRLQREAVFGLMFACGMWMWPSEVAGGGWSIVDALRKRYDTSRGGDANGVDTSGVEDVIQSESAIGAAEGCTNQLDICFLIDSSGSIGIQNFRLVKQFLHTFLMVLPIGPEEVNNAVVTYSTDVHLQWDLQSPNAVDKQLAAHAVLDMPYKKGSTNTSDGLKACKQILFTGSRPGREHVPKLVIGMTDGESDSDFRTVRAAKEIRELGGIVTVLAVGHYVKHSECRSMCGCSGTSDDDSPCPLYLRADWGQLATAIKPMLKEVCKTLPQDAICSDWSAWSPCSVSCGDGSQIRTRTEVSAPQPGTPTCPDCPAPMGRTCVEQGGLEEIRECSAGVCAVDAGCGVWGEWSAWSASCGNATRKRERTRYNDPPPQGAGRRCENQDPPVLQEQTEEATLAPCITIPPTPPEWAAWSDCTVTCGGGNRHRVRNALPPGLGSQNGESDESLVSKLWPGTDLRQEEACNTSPCPINATCGQFEEWSTCSVSCGGGLKTRSRNPWNEDQQHGGLSCEQQHPGGRTETITCNPQACPVDERPGEWAEWGECSVTCGDGVRERRRGKSLVEAKFGGRTIDQQNEALPEDLKIKNVEYEPCSYPACGASCTYVWSDWNKCVCPMGYQARHAAVKFDYRNKPCDLPTFETKACSCGETNPVPSEGTTPGAPGATGPEQPNQRPLPEGSDENETPTNEEGEQSKKESGSGIAGAIAGGVIGGLILLGAAGGASYHYYLSSSVGSPSAEIEYEADDGATKVVMEEEKETLVPVDDDSDMWME |
| **15** | TgTwinScan_0905 | MIC4 | MSVMRTSFVQRVGVSAVLLFFNMEMRKLFHTGYQNALRKTVFREVAHHRKVIHSWTARHVPVLRFRPVCAFVVSSPLYATVQPSRKLSSSSSLAFRSPVFKAFPPAAHPSPAKTSPGRRSLAIFSLGVLCGAAVSWLATRQLPDGWAPTQSFLLGSHAKTLPGDQEGGDEEGVTSERPLSAAQLAHAAAPNREEKATAGDAVVHYPSALLPSAERLLLSTGFASLVSTAHRQPLYVAERLGAALASDDGQALQKGDRGPATDAGDETGEEERHSEADGETDADADGEPETRTEEAPYGTGRRGCSSDDSGSHECSSLGRRRSKAILRRKGERATGGVDRKALLFRQDPRVDKLWSPDRTDYIHSGYSKGHLAAVALHKDSLEELESTFSIGANIIPQEQSVNAGQWFKLEQLTRFLAQVYEDVYIVSGPLWIPHDLSTRTPLVTLVRSPTSASASGPSRSASAPASTKSGEGGESNAAAVQTGRDLWPRRVFFRDFQARDSPAFERKAQGSAPAAARDGDPRTESGWTPYDGAPEFFPVPIEALTGSAFPSSSPSHSEFSFSSPSPAFPSLPASLPSPDGVDTFAWSFPACRQALAEHHRDPPNPQTPSSSSSSSSSSSSSSSSSSSSSAAARRGEKATPLHMSYEVIGPRLVPVPTHLFKVVLAVGPRRTKHGMRQGSGDAVGVPAPASAEEERVRLDFVVMPNEKVDTALDLRAFRVPLEFVEWISGLEFKVEAIVEYARLPLFVDETAASLIAPLRFRITVFLGVYTRSSTLMEIFVSWIHARLCFSLPYTASSPPPPPRWSAQDNYSFLGSWYPMTVRFFHIASVLGD |
| **16** | TgTwinScan_2357 | MIC5 | MDALKTLLNKGITLAVVVAAVLLQIRAVALQYPGLRNQVVNAHSFAEVETTGYSCLEKDKEYVGFSLTEFTEVGDAGLCQQRCNQHPHCSFFTFYSNGNRCVLQSRKPSQEKNNADAVSGPKRCPLCLVDSFDFRGEANLHHKGAPGLNTLLACQQGCAAEPRCKGFLFEKRPRTCHFKTNDNYLKALDPDTSYIAGPKTCTDEHWCIMKDIGYRGTDSKETRANSAAECQQMCLNDERCDFFTWQQAGKYCWFKAGASTASTKYNRAGDYSAPKHCGLPTTCVKERTKYAGETVATFPKSEVGTFESCQMKCWKTSKCVFMHFNNDGCTLSGINATAQTDANSKAGDITC |
| **17** | TgTwinScan_2361 | MIC5 | MDALKTVFNRRIALPVVVAAALLHNSAEALQSPGLRRQLVNAHSFAEVESTGYSCLEKGKEYVGFSLTEFTEVGDAALCQQRCNQHPQCGFFTFYSNGNRCVLQSRKPSQEKNNANAVSGPKRCPLCLVDSFDFRGEKNMHDNGVPGIKTVVECQMGCAAEPKCKGFLFQHKTKQCHFKTSDNYLKSLHPDNEYVAGPKTCTGEHWCIMKDIGYEAMYTRRSQTNSAEECQNKCLNDEQCQYFTWQVSNKHCWLKHGPTIANSKYNRQGDHSAPKHCGLPTTCVKERTKYAGETVATFPKSEVGTFESCQMKCWKTSKCVFMHFNNDGCTLSGINATAQTDANSKAGDITC |
| **18** | TgTwinScan_2359 | MIC5 | MDALKTVFNRRIALPVVVAAALLHNSAEALQSPGLRRQLVNSHSFAEVETTGYSCFEKGKEYVGFSLTEFAKVGDAALCQQRCNQHPQCGFFTFYSNENRCVLQSRKPSQENNNANAVSGPKRCPLCLVDNYDFRGETNMHKSGAPGLNTLLACQQGCAAEPKCKAFLFEKRPRTCHFKTSDNYLKSFHPDTSYIAGPKTCTDEHWCIMKDIGYKGTDSKATKANSAAECQQMCLNDERCDFFTWQQAGKHCWFKAGASTASTKYNRAGDYSAPKHCGLPTTCVKERTKYAGETVATFPKSEVGTFESCQMKCWKTSKCVFMHFNNDGCTLSGINATAQTDANSKAGDITC |
| **19** | TgTwinScan_2358 | MIC5 | METVTTLFNRKIALSFVVAAALLHNSAEALQSPGLRRQLVNVHSFAEVETTGYSCLEKDKEYVGFSLTEFTEVGDAGLCQQRCNQHPHCSFFTFYSNGNRCVLQSRKPSQEKNNADAVSGPKRCPLCLVDSFDFRGEANLHHKGAPGLNTLLACQQGCAAEPRCKGFLFEKRPRTCHFKTNDNYLKALDPDTSYIAGPKTCTDEHWCIMKDIGYRGTDSKETRANSAAECQQMCLNDERCDFFTWQQAGKYCWFKAGASTASTKYNRAGDYSAPKHCGLPTTCVKERTKYAGETVATFPKSEVGTFESCQMKCWKTSKCVFMHFNNDGCTLSGINATAQTDANSKAGDITC |
| **20** | TgTwinScan_1757 | MIC5 | MLRPTVRSSLLSLGLTVILYLALTGSADALASHLRSRHMEAGRRTMDTQNDVESAGRQSEPMEAADRQAEHPGAPTQSEMKEFQEEIKEGVEETKHEGDPEMTRLMVTEKQESKNFSKMAKSQSFSTRIEELGGSISFLTETGVTMIELPKTVSEHDMDQLLHDILAAGGVVGLDSEVKLA |
| **21** | TgTwinScan_1521 | MIC6 | MRLFRCCAAAVVAAESLLWLKNGSPFFAFLPGNGEIADNCSGNPCGGTAAGTCINTPSGYDCRCEPGYVLGVENDQVTCMMPSGVPMANFVQLSEKPAACSSNPCGPEAAGTCNETNSGYICRCNQGYRISLDGTGNVTCIVRQESGCEENGCGPPDAVQSCRRLTGTAGRLCVCKENFIATIDASAHITCKRVPPHYRKPPFEFGKGGHPVDSEPSKRQREDEGESREPESDSTEPGRDQERRTPLEESQEPEGSTPDSQQSRGGSGSDSTESEEQGKEREEGSGHAGAIAGGVIGGLLLLSAAGAGVAYMRKSGSGGGEEIEYERGIEAAEASEVEVLVDLDSKTWD |
| **22** | TgTwinScan_4027 | MIC8 | MKANRIWCFFAWRMVVRASFLKEMDSIFVSAIRQNVQHTHSALLAKLKEPPDPDDENSWLCRISKKYDACGSREYSDKGLKWTFCPEDFCCSKTACFYGSCGSWCHDNWALCSSSIIYHDEYSYGKCNCKRFQENCDVNAICVHANREDGGAYCQCKEGYWGDGKSCKIDFCQLQPCGAGTCTRTDEGYKCDCPETHKLIVVEDKETCKAKPDFCAEEPCGPPSMVENCVNTDDSYECVCKQGYEVRNGRCEEIDLCADKPCGPDEGVHECVTERQPKLRYRCTCKAGFDLTTLPDGVSQKCLKNFCYEEPCGTRDLVESCKSKAYGYSCLCAAGAMVQVINGKEKCIKADLCRNDPCGPETAVIQCYSHGTSYRCLCKAGYTEVFVNGKSSCQKGDPCTLNMCGGNEAVQECTTDGTAYGCTCKPGYSIAIKHGQKFCNPEEECASHCGSAAAVKSCEILDSGGYQCTCNPGYVMRYSDYVKGCVEGNQCSLNPCGEQEAVQRCIPEGDTYDCECNPGFVKRVLPDGNFICADPASCVGNPCGSSDAVDACIAGTSTYTCRCKDGYTPQSIGSKLQCLPESTDQTDFDSKHKPEDNKGRYSKGTIALVVVGCVALLGIIAGGISYARNRGGERDDEDLAPPPRSTRERRLSSMGEGFENASWASSVSMIPSAPAPPPSGGIWS |
| **23** | TgTwinScan_1705 | MIC9 | MKEIAFFLLPLGGILSFLLCLPIQCVQRACISFASLQLSLGRVSLRLSATCFLYSLLRFVHVCTRVARDASPQSAGLGASGASHPNAGAGLALDVRRQAQVLRHQRNFWITLSAVFVWLFVWWLAKLLRWYWRSLAAKQKEVETLRAQVALFTRRNLSAPLAENGSETAKKSEETAKKSEETAKKSEETAKKGKEKRGDAERDGETLEKNSKVQEVELTALERQKADGCREEKNEAKERAGSNETRQRLLYRENADARTLGVID |
| **24** | TgTwinScan_3016 | MIC-like | MKLECFGLAALSAALFAGKAEAVAANCFKADTDYFGYDVDKIFVGAATESAEKCQEACRNREDCFYFTYIPERKQCYLKDNQAPNGTVTNPLAISGPKFCYPTGTCFEYMTDYVGYDVQKIEDQSVSQADDCQRLCASNDQCFFWTFIPSKHNCYLKHNGATVGRTPHSEAISGPKSCTGGSPNDQGVPCYEPDTDYFGYDIKIFDNNLVGTAAECYGLCLSFAECKFWTWVPALRNCYLKSEYAPLGRVHAPGTISGPRDCNGSNLPPFVPPPPIYPPTHPHPPPSPYPPYPGHTSTTTPATPPAESCYKAHVDFPTGTLKQIVTDDAYACQKLCQAEAACFFFTYKADILECALKGSFAANAPIPRRHALSGSKYCADKMPPEFQPLHNHVPTPPLPPHHAIPPAYPGPNPMPVPPTPGFEPSCAELNVEYVSDILSVLGADSPKECQTLCKLTASCNFFTYRSDLYRCYLSKSKGTPSSRLNAVAGPKECPGEGSAVPPTASTCFLQNKMFVGTPLSSIEAKSAEQCQAKCAGDPQCYYFSFQVMSSNCHLFRQVNGAKEEGTCVSGPKTCADKPTDPCFKEGFYFGQVLKRHNAVASAKECQTLCATFPHSCVGFTYESASQKCFLMSQTDGLELSDGFVSGLRQCPATP |
| **25** | TgTwinScan_1327 | No homology | MKMGRHVVILACFAALASTVAHGLYRSPSYWNDPWPETGEDWAEVVAQVKTTSERLYESVLAFRRLTTSATVRYSAYKKEKQLRPEALQLPHMWHVRNEIWRITDDLRNAKDQLRLLIRKQKALISILEGMRGKGDRKHWNDTTSICRKASGELREAEELVEREEEEYNIEVE |
| **26** | TgTwinScan_1114 | No homology | MRTSRLLCAFGAVLLCWVPSWTAHAEEHFAVFSTCRTSFGRKRILVHSGDSVTIQCPGAIASNPQDVSKYVCPGEEPNCTDATKATYQTMFPIAPPDFQFWSGGDSKSNYGILKIPEVKDPVTISYSWQAAHSTFTGHAANSHLILDVNPKEEEVTRTVTSTVVVVSGCETQSLFTFLLVVASGIAMQQVL |
| **27** | TgTwinScan_5459 | No homology | MAYSRRGAHSVATKAGALLSMLVLTGFCVVFVRAGNNEFPATQVADITCSAESMEHAVTLSANETFSLTCATQDWEPVPSKFHEVMCPGQGCNCTDAQAAPYVAYFPLTKYWKWVSPENNSDKTHQWTTPVGKWLNMQEAATFSVGCRHKNNNTLCFVDVTVKAGGHVLVPPVAASFLALLAASAVQIL |
| **28** | TgTwinScan_3776 | No homology | MVWRVSIGCAVLATVLVAGHEPVASAPYKAYGIPAESTDTTSGDDVEEAFRKRSRGNVLKDAAKEEKKLPLFFIDVEDNKGQGRNSEKGATDDDVAVDKTKRSSPSAPCTRLAGKEKKYPCDRYVVKGKMSFLEAIGAFFLASVIHAAVSEQLRARSTGGDIWRPRLAGSVMQAALAATTLWMALRRRLPTFYLLQRRESSEEKKKAKENSTRAASAV |
| **29** | TgTwinScan_0200 | No homology | MAFTSPSSSRAAFLLLVSLSTFASVSNFAVRFEAAAAPADNSGTVTSTTTQAPLEVACSAPVHQQACTLDLTTGATGKFQCSNALPDSKFSKAYTSDGSVVNIPDIVPGAIVTKTGTSGSIQIPDSFYGRACFALTCSEIDSTPIQQSSGNPDSATAVKLIRYTLMVRVNGGTLPGTAFEVFFHLYAVVRLELAALSTQKIVGRREVGLHHEYLSAGAFPVLLLDSRQGFPAADPRHAMEDPPPFPPSGVLSSRLFAPDEAGEEVGPSLLLPSEVEGGERAQASKPLRPGRIYNLHTLRERMKEFTSAEEGGKASRGRKKTPNIVPLSHEALADQAKSQGYERFLEGARKEEWGEGGRGSSCSFAQDVGLTNRSTLGSESRRMHPTKSGPSGASQAFPLADATRFLRRQWCPQYDSSSDHEAAICQRTAHWLLRPSGDTALYPLHGNRPDWAELDEAEKKILGPKNRNYHLYARKEKAGGFGIRSAMDSLEEGERMSSTDRGLGSTTSTLVLNMRTHRLERRSRTKASVEKAAKGRVRWQDEGEESDGGVETGEGGGLLSDEKMLQRVAVLMEAFERSGPNAAGWLFLRDFKWTLHRDPRVQEIFGLEKKQYSLEKKSKRKEGHAEEESTSQGPLKAVVSWIADTVFRNRDEPVTFTKKSQVKVKAMLDQLRRIESDLISNERTVEEAKIAFDTFAKAFWPFVGKSLTPDTWRQGLGVYRTVATQKDTPYALTATQMMVRDSILGVNIASDELRLPDFVELPRLGLMEKPDLSLAHEVGTDRKLHEKAQSLERLRFTEASSPFQQSFADVIHLEKDLAEYQVLKQQLDRVSREAFLSAPSESLARFLAVIRDRAGHMDSVEELAREAGIFRAPPTFKMPSAEHEELREHQTRMREKFGEETTSSQLVGVSAVPTALVSDDEDDEGSPERPVRAAPVIPKNLPPLKSAPKIEIRAKVVPRRSQKLDDTIESDEAVECVRDEEDDVSSLSEDE |
| **30** | TgTwinScan_2661 | No homology | MKPQVLFGLLAGVACLQEATAFSHKRSIRSISSHSTFDDVTAETDEAPVLAEVEKMPAVVKKVKHGMRKVLESLGILDSKCEQKKKHNKIPSAAAGDSPIKTFGYPFYDTSLGRSLLRPQVGLSFIEGFMQPIKVGAFDWMVQGTVDDVLSFLVCFDDYEAIAEKLGEDGAWHSEGNIGPLYLQLQNTSQYQGDASSILMAYSVEQILGRIDTWGVIDQTIGHWWKPAASLSEKAKSVYDTYQSLKERVPDLTTEKFQEVWNSASTKSGVGKLKELAKLFTLNPETRDLRDSWENLDRVDRGRKISLYVTSYPKRSRTMATFAFGENRVKLGDMAAAVAAATKDSTLDNLLKKYV |
| **31** | TgTwinScan_0203 | No homology | MGLSPAFAATAGCRLASPVANSSRFLSLLRLSRPRLNAAAPAAEAAKTLERNVPMKEILQPLWVVEPPNFLRQPVWKQFWEAQFANRSFFFFGNAWTSAAAFAFFIWWSRVFDPPPKERLDRYWLNSPKFRILSAFHNPGKRPGLKISLMTYEARYCYRGLDHPFTLNEMKDFLFKLREQYLVNKYEGIQFPFVFRQFNRVSTPGTLEVHTSPALQQQPHFHEEAAGHH |
| **32** | TgTwinScan_6350 | No homology | MRQFLPLLVLLPLLQRQDSAAPAVQSRFPGSGSLLPSAVVAEATRARHRASTLAADDEETPDDEDTVLSEQEQEAEDECEAQAVVQKMLQAELDGSSTSLMAASPQWKHLKASLEKHMDNKEMCAPLSGSQPVCRQVQKADDTEVVGSATTELKCGGAEYEKLIEKYVHEKNCLAAVVFSHVLAQTWLGHTDPSKASGGGSEVARKGIIDVLVSQYKKAMDSGFLDDTLWYIYQDTATLLENFKSAATQGLNAFNSDAVRKALLRRRETQTAIFCSAGNGGDAHLGANVIVNDGALIFRVNLDGEDGVAVDAFTEAIRTFATTFVQAVLEEGGLLDRAIELHKRFYKFRFFKIQGLMQNAEPSADVREQNKLMNALPMEKHRKTKHAKTPTESASFLQVTSGVKVDPQQLQPVAPISVPTQLAPQMPTPVSPLPLAVVPPTPAVISTTQAVGAPDPYLSSVPMATAVTDMMQPVAAIAPAAPVAPQQLSEVPQSLPLPQMPQPQAPSVQGLAAPTQLTPLPEANTAAVAQPAVTFPTAATTGQTLFTPSEQTPTMPGLQEPMPGLQRPMSGVQGPMPAIAPSTHMEPPLSPLEVYEQGAQHQPLEQHPEVSEPVQSNPEKPPTQEEVPAKQIESRPQTTEQKRKYSGALSALGALDARGHLPEMTYNAAKISLQPVKAVPIKQKVQAIFSRLRMFKMNNETVLYEPDTEIIEKTVKAAYLDTTDRVFDVWGALLPQAATTTTAQLLTLLLPKPDVDLAEFYNKTMNSEGVISDGLQSQLPVNHTRLVERFALFLEEVYRDCWRNFFNVNDNFLSSSSSSETGEKATLSAASIPTVSAVQLSDAKVVDLADGVVRRGLEKAASMEAVVKGHSFVSLKSSTTEKGIDIAIVDSSDGVGVNELAKVFTDEKLIQELTEKALSSGASTLWASAGTLAVAGLVAVTAL |
| **33** | TgTwinScan_2489 | No homology | MWRIWRCRLSFLFATGCLLGALTAGLGSQMSDSVGRNVQAPAGVADASQEAGDVVEERTERTEEHIFALGPPRRHSSESLFPRNPSVTARRRRNRRIALMATAVGVAVILAAVYVLRRRWAQPPQEPEPPAPPPVEDPEVLPEEDEASSSLPPPPPPSPPPPPPPSPPPPPPVEVPLSPESQTVDLSCLSGTTVRFFGPSHHSGGFTPLYDPAPDKRVATVDAGANALFIGGGGLNGQFAKTLLEEAEKNGIRLTPVALSEHSQRIQQSLLRRAVKSPGKLVELDTGVASPVFARSFGFVPVVPGLMWKESKVGANVGVTFIHILKPEVTPYGNLNNNVMMYTVAPCGAAPDTTYSLACESE |
| **34** | TgTwinScan_4636 | No homology | MSELKGKNIFLTPDGRTYHLGVKKGDLASLIVTVGCEQRARVLAAKFLTDVTEVSSSRQFYTFTGKYIKGGQRMSIVCIGMGAPMADFFIREASFVLEGEPLAVVRIGSCGIVDYATRPGTLMLSDESMYCYRNYCHFDGEAALGNKRECGRDPRPYLLTAPVKADKRLTDLIEANLNAQGAAVKRGLNCSAETFFACQGRELPLWNDNNANLLKDMTDLGVVSLEMETHQIFHLMHQRQHSIPNGPKSYAASLMIGLVNRSNPNLTAHVTAEDQDKATLAAGEAAFDALVTLVSEGIIKPAA |
| **35** | TgTwinScan_3416 | No homology | MTDGAKLYRRSMTTATTTGFFLLACWFLPSSCIQGKLLPVTLLPPRKDHVRIQAYQNRRTDVNAGEHVRGAIPTLIRSAMQEFCFADRGGLPYRSVQDFVFMPAGSEALSTTATTQPISNVTRYPEQHTVARSAADVAKALAEKVAREAMKNAYNDVFRNWTGIAHPDFHNGDAETCKIVLSFDVAKKKNAMGSVTKILSFREDELKSLGSQALPQGCTWDGPSAVGVVTRHDRSADANLWDVLLFMADAPSHVQFEYTLPKEADVERNSDLSSGFASGPGEQDDNI |
| **36** | TgTwinScan_1781 | NTPase -dg | MEQAPVRPPEPRDSGGGRRVLEELMLSEVSCRQLTQAMIVVDGGSSKTLPSLFTAVTESCPQEGRRVLEGTLKFVGEGTKVAGVRDLLEDWLDAHAGKDWESRELDAQTLMRSFPSMQEQANKLVARLIQDVVALLNSRLSETEKEEVKALGVPVFFHTTAGVRGFPDWYRDGMFVALRRAINASPPVEGYLFFTNKDWTRAISGEVEGIYAFLTANLLTRNFERLLEDAPGVSKESELHAHRKLAGIVEVGGASMQIVFPVRPFVPFPPFAKVSNLQKEGYLPHSYPPVDLVAVSFMQLGASSASGVFLKMLCGKEEYLRDGICRNPCLFRGFEQACSAGEVTIGPTGEIHVSSNLRKNRLKPAATFCGGGNAEIMYKLSNRIECMATRINPLLPLEQRMQIPNCEKIVGTGDFAACAKEVEDILINPNLPLPANQEAVSLGFETPAQIFKFISSSAPLFVTGASLVTPVKLLQAVNLLPKDFDGTARAQLVEAATKFCATPVSRESDGSLSLRVEASAQRPDAPPAKPVKLTTLNYETCHRLAMAASVLQQIDSGARKPNSVRFETKVTDDGREVGPFGWQAGTILHHVANSRQWSTAAYELGVGHTFHDRKIALADRKVDESLEGTNAASAEAVVAAEPSV |
| **37** | TgTwinScan_1762 | NTPase -dg, NTP2 | MWLPVYVPLLLVFGVSLSLPQGSLGTDSSSLRGVDADTEKRINVGKKHLQTLRNLETRCHDSLQALVVIDAGSSSTRTNVFLAKTRSCPNKGRSIDPDSIQLIGAGKRFAGLRVVLEEWLDTYAGKDWESRPVDARLLFQYVPQMHEGAKKLMQLLEEDTVAILDSQLNEKQKVQVKALGIPVMLCSTAGVRDFHEWYRDALFVLLRHLINNPSPAHGYKFFTNPFWTRPITGAEEGLFAFITLNHLSRRLGEDPARCMIDEYGVKHCRNDLAGVVEVGGASAQIVFPLQEGTVLPSSVRAVNLQRERLLPERYPSADVVSVSFMQLGMASSAGLFLKELCSNDEFLQGGICSNPCLFKGFQQSCSAGEVEVRPDGSASVNEDVRKNRLKPLATYCSVNNPEISFKVTNEMQCRENSIDPTKPLAERMKIENCSIIEGTGNFDKCVSQVESILVAPKLPLPANIEAASSGFESVDQVFRFASSTAPMIVTGGGMLAAINTLKDHRLLRSDFSGDVEELAEAAREFCSSEVIIRTDGPVIQLPNARGEQKLNSLNFDLCKTMALTVSLLRHMAAGENQPSFIKWEKSIAGPDGKPLADLGWQVGVILHHVLFTEEWGRTAYEAGYSHNL |
| **38** | TgTwinScan_1066 | PDI | MRAGFSFALLAVGLLATAVVYSAAEEEAVTVLTASNFDDTLKNNEIVLVKFYAPWCGHCKRMAPEYEKAAKTLKEKGSKIVLAKVDATSETDIADKQGVREYPTLTLFRKEKPEKYTGGRTAEAIVEWIEKMTGPAVTEVEGSAEDKVTKEAPIAFVAELASKDSDMAKLFEEVANESRQLGRFLAKYGASDEKIYSLRYEEGTEAFTGKTKDELKKFVDTESFPLLGPINAENFRKYIDRDLDLVWLCGTEKDFDEAKTAVREAAKKLRDTRSFVWLDTDQFKGHAENALGITEFPGLVFQSKKGRFVLPEATTSLKDAAKISQFFDDVEAGKIDRSLKSEPVPEKQDEAVKVVVGKNFEEMVIQKDKDVMLEIYAPWCGYCKSFEPIYKEFAEKYKDVDHLVVAKMDGTANETPLEEFSWSSFPSIFFVKAGEKTPMKFEGSRTVEGLTEFVNKHGSKPLKKDDKGEEL |
| **39** | TgTwinScan_1683 | putative amine oxidoreductase | MLFMCHLMLLLPLFVFLRMPPRGLHCLLGAPFRCLRISSETAELRAARAGRPTKRERNETEKEDEEKPSEEKPSEEKEGKRKAETFEDLLLRICLCFRCSRPLTADSAQGWLHAASPPREDCLRRCLSPRLCAADVVVVSDGEECSDSERGRGKDGDGGRLQETQDGEGDAACLSPSPVSLSRRRLHDESSLNGTEEEACASFDAGKSHSDDAKAGEDTPPLPSHGSSASAFSLRVGRPAEKKRQFLLRGKKQLKVAVIGAGMAGISAARELRDAGVKSVVVYEARSRVGGRCCSAEVTLHPRPELASASAQAKASRENPGLDIGDREESAEAAQAAKAPKEVKGEEQWPLRACEPRGVLGECCDASAKRSAIEGICSSRAFSASSLPSSSSSSSSAWSSTSAVSSVPSASASLSASASGALVVPGIDLGASWIHGIDDNPVYAICLAHRLHPFGAAKGVEVVDDSATVGSSKRKQKASQKAVEILTDAEEVLFDEATGARVDAEVDAWAYEAHQSHQREADSSARRDEGNRGWRQWPAGPTGAQTELKTLLHQSLGGVLNAKMREAIRARRKRLAQPREETRNARKPRAAPCTEDQEPAERSRTAEETEREGEEETDVEPGKEGDSVEGRLLMWHLNNLEYSAGADVDDLSLICWDQDDLTAFQGQHVLIWEGYKSAVEALTSDLDIRLRHEVSSISYSDSGVTLRFADGTVSPRFDFCIVTLPLGVLKASVTADEQRATGEDAARFRSEVHAKELLPSQTLPARSLSTVRGDNQVSPLIGEEEEKAPDTQAENGDAERVERSSEGLHWQEEAWRVPHSGVTADAFREEGRNAVRLAEARGADSSTSAFLKSEVCEEASAPVKARAARSDLDVDAEIADALHDVPVDFTFPPDLLPFLIPSQKRRGNGRRICKKQALARLRAADGWAKKSREATPEAISSSGVRTPRPPGGDTPKDAQQGVEGVKGEGEERDAARTEEGGVDASSSASSWFALSKERCESRRGLVAFDPPLPEWKREAVKLLGMGNMNKVALVFESPFWLHEDDDGDGDEGEAEDGDKGEAEETRGPETLESVGNGEDEREERRTEDSQVARPRGEEGDASDRRDGSGGEGDRKREEESGKGGLTDADGETRKNGNCRGVEETEMRESTSDERMDANSKGDEQREREGEIGGNEDEMGGREGDTRSALRRCENPTGAKGGRGGEKGGGRSGRRRREKTKGSEAPERPRQAREWRDETEEREDEPEVNAKERRSLRPRRERREQEKFSDSEGRETKRRRTRKNRVKTVPRQRGWASREKGRYAQLVYLHPKPIILVLVPGTFSFLSEKRPKAELVCEALRVVAEIHEGRIEAPIKAFVSRWGKDPFARGSYSYLPPGTTGRDYDLLSYPVHHRLLFAGEHTIRPYPSTVHGACLSGRREAARILDWTAGLMEQHAFLKWFQHAEEGLWFSVDLSEMEAAPSYFEDCLEGPTAAEREEWFAGSIRCAFCGEAQTFQRPFIGCVSVPVSHAPSASSTSPPCEFLAEEDGKTSRDSLLQRGTEAPLSQAGEAIERINQKQRRLRFVVHEDCCYYTPGVVSDSEGSRWFNVGRAILAASLNACACCGLPGASIPCAAAECSQALHLPCAQRELRWPRYETKSAVRPLFCPSHQRLLSLPCEGLSASPRALKEAAGEAETGDDARARREEEEKKAERRNVGMASSWPSGGSSSPSFGRACTPGRPVFPVSALTATVASQRGRILCEDIKPRERGRARMQSGAREEKNLTSELRCLLRRLERAVECLQGPSETKRGPAGERDRSLTVIDVDDSSVHKIRDRKTIIDLEDA |
| **40** | TgTwinScan_1873 | putative insuline peptidase | MTFDQFWEKISQKLKSLSSFFCRRASAIDLLASFKSHCSYLQGLYDRPADFANLGNHLKEVEGDAEQVGRVLYLALPPDVFLPSVKSYRQSCWNTKGWNRVVVEKPFGRDLKSSDKLSASLMALLREREIFRIDHYLGKEMSLSLTALRFANVAFMPLFHRDYVHSVRITFKEQSGTWRRGGYFDNYGIIRDVMQNHMIQLLTLVAMERPASLKDDDIRDEKVKVLKQMPPVKISETVLGQFTKSVDGQLPGYTDDDTVPKDSKTPTFCTCVLWINNERWSGVPFIFKAGKALESKTTEVRVQLREAPAGASFFHEPNLTPNELVILVQPHEAVYLKIHTKKPGLLSQGLQPTELDLSVMDRFDVERLPDAYERLLLDVIRGDKQNFVRTDELREAWRIFTPLLHEIEEKNIDPLPYPAGSSGPSASYDLIQKYYSYKQSNYKWTPPKRETSSDTVQ |
| **41** | TgTwinScan_0069 | putative tryptophan tRNA ligase | MASRTPFQDAALSIVKGAACIALSLKTARTTQNVKFSKDNRCSITLGPIEVEPTAAEEALLKTLTKQKIEENCPFRVFTIPRDLATRMYQESYLDEFGIPANVKEVRLVVLPEWNINANMYPVLKSTGQIADIQFESVKFNEEKKQLCLSLHVVPGPDEQSPALAEEEKLGPEEIPSRAQVLPPSGVEGDDFGEGDDDQTITPWDVQAAEGGIDYNKLLRKFGCSSITPELISRIEALTGKRAHHLLRRGIFFSHRDLNLLLDSLEKRQARLASGKREDGPGSEGLSGPGFYLYTGRGPSSEALHIGHLVPFMFTKYLQDVFDVPLVIQLTDDEKFLFKDSLTLEETHRLAFENAKDIIACGFDPDKTFIFSDLSYIQHLYPVILEIQKKVTYNQVRGLFGFVESDNVGKSAFPAVQAAPSFPTAFPMIFGGKKDVRCLIPQAIDQDPYFRMTRDVAPRLGLLKPALIHSRFIPALQGFKTKMSGSVETSSIYVSDTPEQIKNKINKYAFSGGQPTEAEQRVKGADLDIDIPFQYLTFILNDDDQLKEIGEKYQKGEMLTGEVKAILIKELQALVLGHQERRAKVTDDMVRQFMDPFRPCFKKYAQ |
| **42** | TgTwinScan_4574 | RON1 | MARSSRAAPSGAFAHGVAKASFTWISGMKTPFGCVFFFCLIALWGFSAALRTSDIGVDALSVSSRAAANGSEGGVAQSEQERASDEQRNDSEAHDSVASSESSDAERKDDPPDNKSTSETPEAKLAGRDASNPPASSKSPTPSPAPTPAVSTSTTPAKSSADTSKDPKNKLSSARKEKRIGKKFKRHDEFLADAVIDKLDSMRSVQVSGALLRRIWKEHQRVGDDMRDLLREYILALERELYTQQAEVFGVEQLKMDDVIHHEHSSEARKREVELEMQALAEQHNFMKKLFGHIQANVEAKNTSALVNRIEELDKPIDIIDAIDKRSGSDVLHCLKVIGKSIRYMQESVKKFSRELKTLERLDAERPSGEAERIRETLGFVMDPLSGPLSSMDYESLSWIPSKGQALLHVDKAYHDRMQHDYDSFLMDNDDADFDHSHHYDPFDYNDPFDFDDEDDDDDDVPRKKRTHGHPEKQLHFKRNQGSWKHDFRLPWEKEEAHPWYKGSSKPSRAHSHRQSVSVYASPTEPVEDHEEDSSMNHEDSAVFPGLFFLELGSKRTVGTAADNTPYAKQKSFVQRAQHSVQDIREHRDVDREDGVEGEQPLSFLETRSSAEEGGGSDEMRSESEGQGSAANGTEGESDKAEEAGKTAGGSAEKDKDASEAEGAEEASSEKTDGQGGNDQPSESESAGGAATHGESSTPEEGEGSEKAEEKGGHDKPEENDSSEKAEEKGGHDKPEENDSSEKAEEKGGHDKPEENDSSEKAEEKGGHDKPEENDSSEKAEEKGGHDKPEENDSSEKAEEKGGHDKSEENDSSEKAEETGSHDKPEENDSSEKAEATSHTRSEDHPRCIEIMDPQECVKTKNCFHDDVYRQCFFNCTAIVEADKCKEHQNCRYETLLPKDACVNQGKSFYIRFLQKQIPTCCSSNRAIVYCSRLASAYEPASPLYSRRLLQVLAYNIVILQTGVRTLTADVVQSYQSVSLTKQAFEGIMRGCEEFRDKPSCDMMEKATQRLKESGTPVIYDCHWLSEGPEVSGEVLSDGTQPGDNEEKEVGKEEAAQTQGGRSICVNRLEAPTAEKLLWGTIIAEKERKLQELQISRNLTPDKVCVRPRELANAMLNPDKPFYALGDQVEASEMPARCLVHGNKSGNHLS |
| **43** | TgTwinScan_0698 | RON2 | MTKRAGLPLGRAFLVLILLSAADSLFFSSFPRSALQLFSSVLFTDAAEPDSDATPGLRPQPSPRTFRPTGYQRIEVKTVDEELPEDLKVYTASTRGSSSRTFEVRNAGGRQEGFTLSVLTAGGPLPHGSWSWSGTPPEVQTTGGSQISFGWVPDTETPSLPERNLLQLKRMLRDEGLIEAVQLRAAEKGCPVAVLHNLRQLPVNFREVLHEEYESRSNPAKMYEVANSYVQQRGSDAARWSVSQSVELSLLEMHATSTTDPRGSSAVPSFLETGPQVRVAMTDAVPSGIRVYATPPAPRPVPVQSNQTEKERSPTSKRLVGMQLGLYLICKLAALFGHPTLFLNPYYTEQQLLEAVAQALGIAPPHRGDFENEGNEAQATANQHNGSADQLLAAIEIFRLGPNPYTIGHVLTLMIAYLDYESFFGASPSKPFHSWVSLAASAGNNTGFAMLDEMCDNHRGPKRRGQKHWYQTGGARKHKNRDMLPLHRQLCDALELVLNGVQQIQIDLMDELGKYKTGVEPLVDPATNSARIHTRTCRGLSPVCDYEATILAPVRALEPHEQQDSLRTKKAFNLVTGYGSGHVGQITGSIAEPFSHSWRTRWGKVVADPTAYGEIFERTLWFDDRELMAKSSGALFRQYDRIAKDSMSFGVFMNVENGLLKKDMRSKLEAYISQRKSFVEKRQQSRFAKLRKKIPENDPYALRAAIFLALNYPAALHDRQHTRVSRNKKTMRILNSKWTPAVLKKLMRKVNHKHMAREAKALLLRSLDPTVLSSIVTAFDFITHTQANLEVNQNAFMYHEVRAREVSRQSAAEKGSHRLHERGLVRETDDMIKRWAEHGIPGDIKRRLARGEKLPEGMSFGGIPIPNLTNWDAQLNSKWLEAYNAYLRHPYGRAALNARDPVALLVKDSRDRLQAEAEGTIFLGRIAKRVHQSKNLLRRAGRALKTFFLSLLRENERSEYAVWFGVKVDMRQVIQTCRQINSVAEVVKNDRLYDFITDGWMELVKDVVAGYTKASVRVPGFDTISAANEQLRKEGVAAATARNQGFLSIHYDYANLPEEERKKEFQRSMCMEQCEALWKLVMAFVMPNLQNPKKLKGYEKDFSGAKEIEKLNSPHHVNAFRFSLSVQIDFFDNMLDKTSKKNLKAMKFGASTWFTYAMKLAGQVNSEMGNPNLGTALYMQAAYYGNYIRKWMEQRRKSRKQAIIGVLTLGMMGLYALLNVADIVQHMEDIGGAPPVSCVTNEILGVTCAPQAIAKATTSAARVATQDFLKVGLFAGMAPYLMLPMAVVSVWNILKSEIKVLLQFEMALKHTFTRLKRWLAAPFKNWWAKRGRLKDALFRRASQTYRKTEQETKQPPRPRNLHNPSSWGDTELDSLGVPPEPFVQDFEIKYTTPVFPMSAPLIKA |
| **44** | TgTwinScan_5206 | RON3 | MTTSPRVAKWALRVLALVFLHHVTVCTVCEAKALHVIRPHRNFAGLDVQQSSVADLQDEGKGISFAQTYRERDIWFGMLYFEYVITASTTMTLAFKSMAKYIYTAPFLTKLFASNRWLKYSTLDFPQKQQIIRKIARRASSKSLFSRALPGDRVADLFADMYLRRVTSRLSRTPGFRKPQYLKMKEDLTQLIKATNAPTEAMEVVLGNNTQNMFTWIDSVRQNPFATVKNVVVHAFENGLKGVSGMVEWELNQGCFAIAQQTRHILPFGSLFPGGILGKIMQKLMRSYIMFFHPVLANFKGLLALFLGVLCKVRLPQLINAVFGAIFRAKRRAGRYIHKFFFKTISLRKDITGKILVDDLVRGSGAVMITLLFQLHGVDIDAISRRGGAVESGVLAGQGVWTLSDGLFVGLKDFGQLFRAYFERYVNLTSAIHTYCAKQTTNFFTETELDEFYCDKNKSYLAIIEKDFIGLATNFLNHMIKCLKMDFKYFEGYLANIMSIFYEMTFATLESDIVSAINANQGRAPHTLELEAILKRRQQANNPPKEAEMPRTMFRKPWMRAAIRKLAHGYFSSAAGFALKTLLQQIKPANAEKLLDKAFTRVKEGSKLIRSSLTVILGRFFNSQALIVRAHDQTVRMRMYEFINQCQNPEDPNHRDVCGNNVTTNIDEVKFVSKSLLALNLGILVSPRDLLLSQVIETDPAKDSIDPTEFFAEWEAWLQAKGYKPHQKGKEYLLFAEDINQKANIIAFVEALRHTLRDRLRFGRFDQEGADNLRLFDVNGRSAIFRHRIKEVRPDPLDAPATFEVLDYNEGILRFSSKPGIPFEGRLEYADDRRELRSIQQDPQAIKELLVSQIMQIKPRLSAEMTATLMMVLHRYTTSFHRHSFATFLQNLSPKMFFDGVLAFADQHQQQAEATLHLDRRLYSFPMTFQTFKDIVFNSLNTMVLRMHSLDTADSLSVFLVGLVHYAYQKIQKNRTGRTLVMQHFLRGLVHVMRSVDAERFREDFPECQFLIEPNGTIDEVVAIKCALYRFVKLRSLELPRAIAPDVEESRMTEARARTYLNGFVEYLDRLRSERTSWASFLNFKGFLETAELYRETVGAYSYEELERQIKTEPITDLDVLEQLRALMDPSLGRIVAPKKDEGTAQVIRKIWGNLGEFSSGTRQRVRSALSQMRSRLVSVMGSTTLPKAMRLFNVMNSSQFFGALEFAEFRAFPDTRTPPEDILKASDLALFGRVLLVLETTPLYLDNPKQMSDIVMQMRSIPFISPECQELFREIEEMVRHMSHEDVRVAVGWLLTFNIGKGLTGSTVALGLLALAGVPFANSQISHQDKIGILRTLDADTLRFIKKHLLMPMVFRHMASLSDGEAMNLLRFVPKATLLVAYPASIRYLPLSEPVNVVDAVDKLIASKAIDAVQQHLGQVRQLPGEASHDAVCRTMINNIKVELGLSTEDAMKFWTRSHMFFDKRLPAACILLIHLKAMREQNIRETTVAALVDMLYYNGTALNDHLAFIAKKGANELERQKQIDRVGSLLTMVYVAVVSGSLQVDASFMGYSGETKNLTFHSRRSSRGTEVPLLTACIQATTHIMGPPKEKPEIFQASLSSYFLGEWFRFVETSRLEEYAAAYRRGGGFQNISRETLKNISSVYNYYDIGDYFISIRQRSLYPQNEIVKTASMALIQDEATIDDYGMAMLTVDMGHRVRKAKIKCYEDALKDYVEYVDKSRSAQEMFKIDCVHNYFIDMGSLGYSAASITPEQKDQIIASLDRVFAGKCEDMYTALIGSQPDIHKLKQQAWAFATLAHNSLNSATRLGSIVNMLHNLITDIAAGTPWNNVVDGIAAERANKIWERLGDVSHRTPIYTAIDRPYLFNPFAFLLDFLLYVKTNDGMHCD |
| **45** | TgTwinScan_5626 | RON4 | MAIKNTLTGSGLLVLLTLACGTTVQSSPPTPAPRMYPNMNGDNNSKVESIIAGSDTTPRSAEAKGIYPSLDELRRSQETGGQKVVTPIPASKGIYPNLDELRQTQEGEESAPQPADVSRALKEQFFQFLQHLSADYPKQVQTVYEFLGWVADKLPENEEEVQMFIDALNTTEAMVGKAARWIFKAIPERERETIYSSFYQMFRDKLPKKFFETAEGMNPDVGQYFSAEEPVAVTPEIPAKRPLTRVPEWTPVTGSCALGDGYTDIDVTKATTDVLFRITLLILQQIRRKKTERGKLEDDQALVALCSAAGAFVDAWQHQQQALILEDPGTPKAHAQLIERLRNAGKYFMKSYDETTGESDHQQWKKNKAEVSKLGKSALMKSCVKYIKASGDVATRPFDSGTAKYPSRSLYGGIANTLETPFADSEAVAKAVHDYAKEHKKPEKLVGLCGALQISGYFKKCFSDAGRLSSVSFFHQHVDGASVLVRTLARERPIGRHALSQAICDPNISAQYFEGAFRLFSSTVSQEWEKENLFAQLASWTGKEMVLAPSLEEQAAVPPAQPAYETVYGDEEDRIYRIHVSGRHSSPQEVLYVGGIPSTVKPQQVHVLGPTVSDESRRVIHPVRHRSRTAPSSEAASAAAESSDEDPLPAENATAFLGVTPQEEESDAYKHTLDFDAVSPRKNKNKENRISAPLKQSDTLIEESTSKTSEL |
| **46** | TgTwinScan_4546 | ROP1 | MEQRLPIILLVLSVFFSSTPSAALSSHNGVPAYPSYAQASLSSNGEPRHRGIRGSFLMPVKPHANADDFASDDNYETLPSFVEAPVRDPRQVPGRGEAALGTEETPGQQPPVALGSAEGEGTSTNESASENSEDDTFHDALQELPEDGLEVPPPNAQELPPPNSQELPPPNSQELPPPNSQELPPPNAQELLLPPPNAQELLPPTEQDLPPPTEQELPPPVGEGQGLQVPGEHGPQGPPDDDQQLLLEPTEEQQEGPQEPLPPPPPPTQGEQPEGQQPQGPVRQNFFRRALGAARSRFGGARRHVSGVFRRVRGGLNRIVGGVRSGFRRAREGVVGGVRRLTSAASQGLRRAGEGLRRGFTRVRGALRGRGRAADGASSVRERFGAASGRVRDAFSAGLTRLRRRGRTSGEEGRPLLGEGREQDDGSQ |
| **47** | TgTwinScan_5042 | ROP10 | MGRPRWPLPSMFFLSLLCVSEKRFSVSGLHLRFRESPQWDSLLPLQDRRAVQPSSRWDSSLTLEAHAPQRIDTSDAGDDDLFLDATQQVYRGDPVLETSSLPPAALPVRPVSSKTEDGFSGSREEESDGGVPTGSDQGEVWRSSSLVDVSSPGDRGGEEQESSAQAKGEADTKDEAEKSEDQSQSSTADPNTPTAQGTDASESGEDSGSDTMGDSEVTDIPVDPLNSGPETASSGETGQRIGAESGAQPSAEASQDSAAASETIPAADATSPTDLPGTLAGATVADDSRQEATTDGLEKSPGEPKEVDVFNPPLATKSDVAPLSVSTIRAQAAPDTEANIGAEVATGNEGAPEVEEATEDVAGTGADMSTGTDAATEDVAGTGADMSIGTDAATEDVAGTGADMIPGAEPGTENEASTGAEGATEADEQVPLKTEAQGSAGSSPTQGHASHSVEETGRFSVIPFEFGSSPRLRRWTTQFLIRGKDLFNLMQKSNIAKQAEKGAKNLRSSMEILHNTTSFFNAFRAQQRSKEAQSKNVQLSVTSVPTDQPEEQNATTQEGSTASPEEAAREAAEAIGTPVSTEDAPN |
| **48** | TgTwinScan_5542 | ROP11 | MVDSCARLACFCIGLFFCLAVWQHQAGHAYSATHHHAVNSDNKSFNDSAPHWTLNQVLRDGTVQSESPSYLQHSSLPSNISPALELAALGQDPVRRRPQPVQVARTGTTTTGRVQLGRQVARVTPEELATMVEPSLKDLLNRSTLPQTPFVIETLRVFATPEGDTSLPYTMERILGAGAASIVIQAVQETVPQEPARRRRSWFSRLRKSHAPADTRPRVALRFPVVDVRKKQQELPEVPEAELLSQLAEEQLRLEIHKVALVSSAAGTEGNFDSEWGFALPFRVGRLSNEGLVLAMDGSGKAILNFVTVSPAMMCDLHTLRSSSRTPAMDEFVVMRVLQLAANLERLQLTHLDITDENVLVGRDGQLFLGGFQHVRAKDGGISCGKIPSVTFTDPRLAHCAVNNPGSYAAVNPAVDAWMAGMMLLRWFCGDVFFNRRSSLAEPRQAVQVLAQLQADFIAANDYMVSFPAFTQWDRCRHHVNERFQFIIQSLLDVNPYKRGAPSAQLTAYFPVTG |
| **49** | TgTwinScan_3842 | ROP12 | MARVLPCVFLVAYLSCFLLAGELTHTTALSVAPKAQLFSTDEGPVSESASKLDAELEEKAIQEAKEAQEDEVDDIPTDRNAPPTNGDPTDQPDVQLNAVDIDGFPTPEVPLPVELDAEKLQEDEFNREEEADNTVPNILVGPAQTPEPVAIPVTSLLAPAPDGIVVKRQVVLDSESGAIQPFLLWGQKSCSTRFRLYYDCVVTWDKDDYCEREALLS |
| **50** | TgTwinScan_4757 | ROP13 | MKRTELCIAALVAVGAFAFTSPNAVAKSFERSLGHLDASSFLSSPLNSDVELEHVAAGNPSAVELLHLVNVGAGVASWVSILSEKLLGLAILNEFFAERLLSAVHTAGRYCGGAHMWAPRES |
| **51** | TgTwinScan_5015 | ROP14 | MQVFLRALGFVYFFAFLIAFNQSQGLIGSDGILPASDYVAMVKDSFADAEAWQRVKAFPSLFLLFPATDFWLHAIPFVGMILSLFMTIQGASNGFLLFLLWILYQTVNSVGQAWFSVGWEAQLLELGFLAIWMAPFWSFSRLPPSWPTPKICVWGNRWLVFRVMLGSGLIKLRTDRAWKDLTALDYHYETQPLPNPLSWYFQNQSHGVHAAQVVITHIVECVISFLVILPFRQCRLFAGIVQIAFQVGILLSGNLAFLNYLTIVSAIMCLDDHFLTCLFPSAILSRLPELVRGCAGYWSLPQIQAWPFPQLFPGEEHGKGKGRQANDRPEAEASLLDKDAGRDETLPFNAATDDSLDSPRSCCCWYRPYFPRIVRSLKIPSRVRKQVKEEIKENWGKMLAEVVASVAMLTCMAFVTNPATSAVWMVIAVFFLAIILSASSTFFTNTLVSNAFTELVLLIVTLWSCIAIYQHGPAVWSVWLTTCLFTVLIAFAYTTLTNAALIFKIHVEVLIFLLIAALSVPVVANLISPDQVMDSDLGNPFSVVNTHGAFGYIGKERYELIIQGTNASSPTQTLYWQNYEFKCKPGDLYRRPCWAAPYHYRLDWLMWLASMGDKETVKDMHPWLSNFLSRLLQNSKSVTGLLAHNPFQNSDPPTALRVLRIQYRFTKGPVFGGGPWWEVVPETRELFVAPSQVPGAAAHLRKQLLILRRREEKQRLEQRRKEEAEIRRKEEEAFKRRRERAMKEEERRKREAEEAEKRRIAEQARQAEDEKRIREQAEAARKAEEEAVRKQIEEEKKRHEEEEKERQAEEERERREQQEEEEKRRAEDEKRRKEKEQATLLARQRSEEALKEAEKKARKDAEEEERKRKEDALQEEKRQKEEKEAEEKRRKQAEARREAARAAAEKESEKARQLQEQEAKRKALEEQERRKARERQRSASSQVAVYTAQPHDGGGVPLRAPLLTRKKQAL |
| **52** | TgTwinScan_1037 | ROP15 | MYNPLKTAKDKHDASLLGLVSSVPDSSILKPEFVGAGAALVAVTRSVKWTQLKQSLKALVDTGVKCKVNCKHNNKKLSKNTDFKDQQEFPQTSARILPTSDREPLIAGPQKVTEEDLQVLLFEIAHDCKMSAPQAAITKPLTILLAGIPGAHISKRPFEAAIPHFILQKEDTVDLLLTVDRSWCGKGLSHLEVDDETHRMQEQNDWLLSGLGL |
| **53** | 27.m00091 | ROP15 | MLKTTPAFFLFLTWMFPRCDGFVLTHKNGTTDAYDSAVLSFLLTTEGVREGAAKSSGTEIPSTLACPSSSPLKTAKDKHDASLLGLVSSVPDSSILKPEFVGAGAALVAVTRSVKWTQLKQSLKALVDTGVKCKVNCKHNNKKLSKNTDFKDQQEFPQTSARILPTSDREPLIAGPQKVTEEDLQVLLFEIAHDCKMSAPQAAITKPLTILLAGIPGAHISKRPFEAAIPHFILQKEDTVDLLLTVDRSWCGKGLSHLVRYHVGYMSFNLEVNGRRLADDDTFEDDRLYRQVVLTTKFTAKSLGVSL |
| **54** | TgTwinScan_6965 | ROP16 | MKVTTKGLAFALALLFCTRCATARYMSFEEAQKASEAAKRQIATLPSPDSTLSNPEEGLEEVQQLKAAAAQLLVAVPDYEAMRAVLQEAVLSEQRVATRKRKRKQPPGAVESAVDEVFPPNERVMMINANGVPIALYNRGHLGSGHFGAVIKASLDDGTLYAAKVPYSQIVPNADATSAELEAEISSARAELVKTIRQELDVRDKLVAKGLTLTETAEQYGLPLCQMTLTLPENKATVVRRGSRLVVVSKEVMLLPLIDGSPSNSLVQSQPPFLFQRAVAREAIIALAKLHELGFAHGDVKLNNMMIDVHGFGHMLDMGSVRPVDSCHVHETVYQPISKATHG |
| **55** | AAZ73239 (NCBI) | ROP16 | MKVTTKGLAFALALLFCTRCATARYMSFEEAQKASEAAKRQIATLPSPDSTLSNPGSKHRNRGGSPAAGQPSQSTLQPEQAAAEVGLGAGGSTQGQGRTGGSAGAREERRSPSPQSAYPATSSASLRGYQTQLSPSHLPPRSSGPGGWFPTESIFTPWSSPPQPLTQRKPSLSGVVVTEFQEPQEQYGAASSLASSPKRYVSGASSSALSGKAVPTPASLGQENPLFPVQSATLDSGIQSPAQERRGSPQRQIAMSTENPADSGASQLASSVSSYVAVQTPHVKRSERIRRVRLSEEGLEEVQQLKAAAAQLLVAVPDYEAMRAVLQEAVLSEQRVATRKRKRKQPPGAVESAVDEVFPPNERVMMINANGVPIALYNRGHLGSGHFGAVIKASLDDGTLYAAKVPYSQIVPNADATSAELEAEISSARAELVKTIRQELDVRDKLVAKGLTLTETAEQYGLPLCQMTLTLPENKATVVRRGSRLVVVSKEVMLLPLIDGSPSNSLVQSQPPFLFQRAVAREAIIALAKLHELGFAHGDVKLNNMMIDVHGFGHMLDMGSVRPVDSCVSEEDKYYLRLWAPELAKSQHTSQQTCLKRGALDVWALGLAIFEFVCFNRLPYSLSNLPSSLWSRVEHLSRLRLSDFSAKDCNESDPAVMGIVAQFLNPNPEERPELPKFVSSYTFFRQAPGVTSHLTRIPTTELSSHRM |
| **56** | 55.m08219 | ROP17 | MELVLCFVIITISGVIRESSALLLRSPTSNDVFGELVASAERAQPLATRLTKRISRLNFNDREDDFWEDHGDASWNNSYTLVNGRTTLGSENRRRPASHSLIERPYYRDGRLSPVLGVQERRGRSVHSYHEEPVSFFDQRAFDEYTFRRRSQLHRQRARAGLRSRIKQNVRRLWTSARGAVRGWGRRVRRKIGDLFVGHLMPQLRRLRFWDQGLPPVVPPLIGNEPGQASVALVAERMEARLREKTLTEKNPTEAQQAVGTYLINSAENTWFISIPGGRYILLKKRGFLGGGGFGLVYHVEHPTTGQPFALKIFVQRVMNNEVGDKISDLIEDEFGVMKYFPPEWTPARMYSELRFMVPLLKLRVLGKPEFQDARNHLRIFSVCALFPKAQGDLEEAAALLADMDRTNAYNMRMSSTIQMVKLLARFHAFGLVHGDVKLQNFLVDKSGLLLLSDFTQILRTNERRYPPVVTVLYMSPEIATCLITRLRNAIPYTAEIDSWMLGISLYRLWCGDFPFGITLDATALQVAGIVIRSSASSLDFASCHDIPEQFREMIVGFLRKTPGVRLSPQQALEQFSLLNWKGPSPASDTASESEPVSTEEAALLQKE |
| **57** | 20.m03896 | ROP18 | MFSVQRPPLTRTVVRMGLATLLPKTACLAVLNVALVFLLFQVQDGTGITLDPSKLDSKPTSLDSQQHVADKRWPATVGHYKYLAGATESTRDVSLLEERAQHRVNAQETNQRRTIFQRLLNLLRRRERDGEVSGSAADSSSRPRLSVRQRLAQLWRKAKSFFTRGIPRYFSQGRNRLRSLRAQRRRSELFFEKADSGCVIGKRILAHMQEQIGQPQALGNSERLDRILTVAAWPPDVPERFVSVTTGETRTLVRGAPLGSGGFATVYEATDVETNEELAVKVFMSEKEPTDETMRDLQRESFCYRNFSLAKTAKDAQERCRFMVPSDVVMLEGQPASTEVVIGLTTRWVPNYFLLMMRAETDMSKVISWVFGDASVNNSELGLVVRMYLSSQAIRLVANVQAQGIVHTDIKPANFLLLKDGRLFLGDFGTYRINNSVGPAIGTPGYEPPERPFQTTDITYTFTTDAWQLGITLYCIWCKERPTPADGIWDYLHFADCPSTPELVQDLIRNLLNREPQKRMLPLQALETAAFNEMDSVVKRAAQNFEQQEHLHTE |
| **58** | TgTwinScan_1315 | ROP2 | MENCASVRSSSCLIWLAAAFFVSALGHVQQGAGVVRPRHWQNSEAAVSVRPPGGASPRPFHSPIEPVAFIDGEHDEDKHEGSWLEQEAAEDVTPLPDSHTEAQTQSPSAFRRLTRRLRFWRRGVTGGSDAGEEAPQTPRPSLRTRILQYLRRVGTFFRRDIPAAALRFFRRFRRVRQPVFPPDEFPEDVDTNPIYFRGTDPGDVVIEELFNRIPQANVRTTSEYMQSAADSLVSTSLWNTGQPFRVESELGERPRTLVRGTVLGQEDPYAYLEATDQETGESFEVHVPYFTERPPSNAIKQMKEEVLRLRLLRGIKNQKQAKVHLRFIFPFDLVKDPQKKKMIRVRLDERDMWVLSRFFLYPRMQSNLQTFGEVLLSHSSTHKSLVHHARLQLTLQVIRLLASLHHYGLVHTYLRPVDIVLDQRGGVFLTGFEHLVRDGASAVSPIGRGFAPPETTAERMLPFGQHHPTLMTFAFDTWTLGLAIYWIWCADLPNTDDAALGGSEWIFRSCKNIPQPVRALLEGFLRY |
| **59** | CAA96467 (NCBI) | ROP4 | MGHPTSFGQPSCLVWLAAAFLVLGLCLVQQGAGRQRPHQWKSSEAALSVSPAGDIVDKYSRDSTEGENTVSEGEAEGSRGGSWLEQEGVELRSPSQDSQTGTSTASPTGFRRLLRRLRFWRRGSTRGSDDAAEVSRRTRVPLHTRLLQHLRRVARIIRHGVSAAAGRLFGRVRQVEAERPQPVFTEGDPPDLETNSLYYRDKVPGQGIIQEILRQKPGIAHHPESFSVVAADERVSRTLWAEGGVVRVASELGQPGRVLVRGRRIGLFRPGMQFEATDQATGEPMTALVGHTVLEATARDVDSMRNEGLAVGLFQKVKNPYLANRYLRFLAPFDLVTIPGKPLVQKAKSRNEVGWVKNLLFLLPPTHVDMETFVDEIGRFPQEDRPLADAARLYLTVQAVRLVAHLQDEGVVHGKIMPDSFCLKREGGLYLRDFGSLVRAGAKVVVPAEYDEYTPPEGRAAARSRFGSGATTMTYAFDAWTLGSVIFLIWCSRAPDTKSGYEYSVEFFFSRCRRVPENVKLLVYKLINPSVEARLLALQAIETPEYREMEEQLSAASRLYSGDGTLTGGDDDMPPLET |
| **60** | TgTwinScan_0488 | ROP7 | MGHPTSFGQPSCLVWLAAAFLVLGLCLVQQGAGIQRPHQWKSSEAALSVSPAGDIVDKYSHDSTEGENTVSEGEAEGSRGGSWLEQEGVELRPPLLDSQTGTSTASPTGFRRFLRRLRFWRRGSTRGSDDAAEVSRRTRVPLHTRLLQHLRRVGRFFRHGIPAAAGRFFRRVWPERPQPVFTEGDPPDLETNSLYYRDKVPGEVIIREVLGKVAGFGPTSGHGVFAAYENAFSEMLWAEGGAVTVISELGRPGRQLVRGNLINIVDGGLLFQATDQATGEPMTVLVGSTWNKPSGKDLDKLRHQALAIGLFQKVKNPYLANRYLRFLAPFDLVTIPGKPLVQKAKSHNEVGWVINLLLLLPAIEIDMGRFVEELYELPTEDRPLADAARLYLTVQAVRLVAHLQDEGVVHGKILPDSFCLKREGGLYLRDFGSLVRAGTKVVAENAQGFSPPEVRGSRGGLLFGPRKTQMTHGMDAWGLGATIFFIWCFKAPTTGPEEEYSIEFLFSLCRRAPENVKLLVYKLINPSVEARLLALQATETPEYREMEEQLSAASRLYSGDGTLTGGDDDMPPPET |
| **61** | TgTwinScan_2103 | ROP5 | MATKLARLATWLVLVGCLLWRAGAVQLSPPNSRTNDLASGTPHVARGDTEAQSGTGDDSDFPQGVVEEVADMSGGRVPRVPASSTTTSASEGIFRRLVRRLRRGRGTADGAGVADETHQGPRPPLRKRLAQHFRRLRGFFGRLTPRWLSGLGRRAQRWWRGRQRPLLDPSFHGLEAGDSFMRDLLKHEKELIGYCREEALKEPAAMVEAVMATTLTRRFLNFDRRRRLLPLEAMETPEFLQLQNEISSSLSTGQPTAAPSVA |
| **62** | TgTwinScan_6684 | ROP6 | MRSSVGQSRLPLKFFLAPFSVKNSVFALFFVFALVCVSGLSWEDAEDGAAWDSVSNDSDDSFAKGSDFGEVKLGSAGQRQLLSQLQNELGGEFEDADVSMLQRDHGIHGEEAGLFRKAVPGLDDPAEDDEADGESASDEAEADSDVLADDEEGTSLIENASEEDTDNSEADSQQEDDSVGEDSFLQQEGEDSEEERAVEDPYAAAEPSYLEEDNTVDDSAAEDYAPASFVQIGSGERKIRAHMHLDSRQVAPERFAHAFNQDHVRLLDQTAVEDELLDEAAPGGGASAVVSPIDENPAEMESTISEGEAGSAVAAPEQGIQPEAEFATASEEPRPLEPVDPEMAAQQPQLPQEAMPTENADLLGNQPRMRNALEPSAKVLEPETLEGSPALVPPAETEEGTAAQIAEEMSKQDQGMQEARPQEVLTAVANSGSQIITAASSVALAGLLVAGQLLFSVGMY |
| **63** | TgTwinScan_1314 | ROP8 | MRHGPSIGSSSCLIWLAAAFFVSALGHVQQGAGVVRPRHWQNSEAAVSVRSPGGASPRPFHSPIEPVAFIDGEHDEDKHEGSWLEQEAAEDVTPLPDSHTEAQTQSPSAFRRLTRRLRFWRRGVTGGSDAGEEAPQTPRPSLRTRILQYLRRVGTFFRRDIPAAALRFFRRFRRVRQPVFPPDEFPEDVDTNPIYFRGTDPGDVVIEELFNRIPQANVRTTSEYMQSAADSLVSTSLWNTGQPFRVESELGERPRTLVRGTVLGQEDPYAYLEATDQETGESFEVHVPYFTERPPSNAIKQMKEEVLRLRLLRGIKNQKQAKVHLRFIFPFDLVKDPQKKKMIRVRLDERDMWVLSRFFLYPRMQSNLQTFGEVLLSHSSTHKSLVHHARLQLTLQVIRLLASLHHYGLVHTYLRPVDIVLDQRGGVFLTGFEHLVRDGARVVSSVSRGFEPPELEARRATISYHRDRRTLMTFSFDAWALGLVIYWIWCADLPITKDAALGGSEWIFRSCKNIPQPVRALLEGFLRYPKEDRLLPLQAMETPEYEQLRTELSAALPLYQTDGEPTREGGAPPSGTSQPDEAGAAEAVTAI |
| **64** | TgTwinScan_6339 | ROP9 | MPLVRLFFTIAAPLLFSPSPCPFLPLKTHCLAIQLGKPQGSPPASQKEAIRDTGVSHQKGEPSDSSSEPKPQGTVAETPGAASAAAAEVGRPSRSSAGPGKKKRGPSLFTEETMGPSKRHPCLKTLAAAVERFQAQHLTGRDAAKAFVDAIQECGIQVVASDYDRTAISVHSDKGENRGDRIAAGPLVEATLKASNANFEAQGVFGYYPPFYSEPEDYAPLGLSAPMPTDKSFHIQQVSKASGVPEDKILLLDDDRANCVNFCRSGGAAIHVSGHEGFDFGAVRVVVKPSLIMQ |
| **65** | TgTwinScan_5949 | SAG1 | MFPKAVRRAVTAGVFAAPTLMSFLRCGAMASDPPLVANQVVTCPDKKSTAAVILTPTENHFTLKCPKTALTEPPTLAYSPNRQICPAGTTSSCTSKAVTLSSLIPEAEDSWWTGDSASLDTAGIKLTVPIEKFPVTTQTFVVGCIKGDDAQSCMVTVTVQARASSVVNNVARCSYGANSTLGPVKLSAEGPTTMTLVCGKDGVKVPQDNNQYCSGTTLTGCNEKSFKDILPKLSENPWQGNASSDNGATLTINKEAFPAESKSVIIGCTGGSPEKHHCTVQLEFAGAAGSAKSSAGTASHVSIFAMVTGLIGSIAACVA |
| **66** | TgTwinScan_7361 | SAG2 | MSFSKTTSLASLALTGLFVVFKFALASTTETPAPIECTAGATKTVEAPSSGSVVFQCGDKLTISPSGEGDVFYGKECTDSRKLTTVLPGAVLKAKVEQPPKGPATYTLSYDGTPEKPQVLCYKCVAEAGAPAGRNNDGGSSAPTPKDCKLIVRVPGADGRVTSGFDPVSLTGKVLAPGLAGLLITFV |
| **67** | TgTwinScan_4067 | SAG2 | MATGASRQDPFVSDKRRIAGPFSWAFSANLVPFFLFALLAVYLRSELPAAAMGKSNVCTYKSIPVILRIKKPGEAVTFKCGEPQPHVLPAKVDDEYKLYCQDSLCKATAPLSDVTITTTAGQERWDTEYKVTAGRTLPERPYTMYFVCTSMESDFEERGNLEVSRSDSYTIYQPKMCKVQVSVWGATRPQSFDKKYECGDNVSQITHTIKERDSSVTFRCGPGRFLSPGILDVYVEPPTYSNLVSLGAMLPFADLHEHASSTRDDIPAYTLAVRDLQKTRERKLGYHCVPDSLDTQICSVVINVINTDYDPLTGVVGSEAPSGAASSLSLVLVLMAFLPSGCS |
| **68** | TgTwinScan_2096 | SAG3 | MQLWRRRAAGPASLGRQSLPLGCFFAAFGLCVLSAILGTGERGLFVAAGNSRRKITYFGTLTQKAPNWYRCSSTRAKEEVVGHVTLNKEHPDMTIECVDDGLGGEFLPLEGARSSYPRVCHIDAKDQDDCERNRGFLTDYIPGAKQYWYKIEKVEQNGEQSVLYKFTVPWILLPPAKQRYKVGCRYPNHEYCFVEVTVEPTPPMVEGKRVTCGYSESGPVNLEVDLSKNANFIEIRCGEQHHPQPSTYTLQYCSGDSVDPQKCSPQSLTNIFYDYSSSWWKGKLNGPDGATLTIPPGGFPEEDKSFLVGCSLTVDGPPFCNVKVRVAGNPRKWERGGGGHPGSGGLQPGTDGETQAGTGSSAGASSRMASVALAFLLGLLVHVAA |
| **69** | TgTwinScan_0323 | SAG5a | MMERQTSFIKRSLTAVGLVAAVLFNACPSEVTNVLATGQSIVLSCTRSENLTTCACKEVSAANRQVGSSTGTATLSELSNGLTIECPKTTYEFVPKEPTHVCIAKESTSAILQACKATPTKNPIKDLLIQTTQTKVPEWTTTNPGGHSLILPPETFPIVDKSFFAGCLKTSSRRQRNTDVECLVKVDVKARTSAVRDGVLICAYGDKSNTSVPEVTLNAENNSLTIQCGEEGEMQPDPKSLTAYHCTDTNIENCKTVVNLTEVMPSFAKSWWTEDDKNGKAPKLVIPEGGFPAQEETIVLGCNVRSKVSSKGQKEQANTTATLPTCRVKVTLTAQPAASHAPTPSCLGLIAVVFTPFVSFGAY |
| **70** | TgTwinScan_5950 | SRS2 | MATRASLARQKTGAACLAVLTASPSVVAAKPWKRWVGVSVILASAFAVGVSAGPPYRYEPEKFTCRPKKGILSQWVSLLYQVQHNITFACEEATPVPTTLISEEHGLMVCAESMTPEECEANPAPLSAFLPGATKEWVTGDSVLTGLKISVPESQYPANAKSFRVGCRHNTKTGNTCMLTIHVEPRDPAVERQEARCSYTENSTLPKIFVTKDSNTMTLACGPHGAPMPESYTENYCSTPDTCDEKPFTSVIPGYLSKWFFGDPKSPLGAMLRIPPEQIPSSPQIIYLGCTGPTDGEGPKYNCTVPVQLGGEDPSEGSRPGGGSGGGKRGG |
| **71** | TgTwinScan_5948 | SUS1, SRS1 | MVRTSLIEHRTGRRVVAAPDRGSLQVCIVFNIALSATIAAMMTSPLSVASMTSPLLTWDGNKVTCHPEKGDVDDWIGLVPQQNEVTFACEGKGVTPVPKNLVNSKSREVCAQGMSANACEKDPRPLSEILKGAQNDWLNGEDLSRGITFRVPKNNFPGLPRIFRIGCRTRSNICMISLHIYAKSAYTSGQVTECAYESNVSLPPVRITPATNHATIVCGPYGGISPLDYKKEFCTGEPHTSCGRQKYADVLPRYTDAWWNGDPSTAEGATLTVPNGHFPKETQTLKFMCLERLDTQPRRCVVTVEIAAGVSGSKPSGGESIPPQAPGTEGSRKEAPNTNNSGGTPTGGGSDDDTTAPGTGSSGTDSRGDLGDGRDQDAKGGMSRGNSESGSNGGATQPSRLLGLPSAVIGTLAAAMFGAV |
| **72** | TgTwinScan_0894 | TgPI-1 | MGKNPLLFLAGVLVCSFSVAVFASPETKVCSCPRNLELNCGGDHVTYANHCIRECHGVGLLHDGPCADQGCACTRELRLNCGVDGVTYSNHCVRKCERVKLLHEGPCRGGPGRTLFSQQDDESENDALEEEEQRLQSCPCPLNLMLNCGVDGVTYDNHCLRRCRRVELKHEGPCEADEQKQLPVQHGDNEIDVPERRIEDLKDCACPLILTPNCGTDRKTYPNNCVRECAGVKLLHEGPCEGDTEEIWDDQ |
| **73** | TgTwinScan_1180 | Toxofilin | MAQYKSRPLAAVLLLITVGSLLTASESVQLSEGMKRLSMRGRSPSPKTGRFESGDEGTSTMSPSVAARQQELGLLRPEERLIAGQAKAAALQTVHQLGAVVLTPEQAKAALLDEILRATQNLDLKKYENLNTEQQKAYEQVQKDLSLLSPETKALLIENHRKEKSLLEQAKRLFRKRHYHVTRQAALAGQILNEQRDASGALQSGAVKAAIRKANEQYNVAEEDKNFNEEQHAAQLKKVGAMP |
