# Supplementary material for: Differential Regulation of Effector- and Central-Memory Responses to Toxoplasma gondii Infection by IL-12 Revealed by Tracking of Tgd057-Specific CD8+ T Cells
Source: PLoS Pathog. 2010 Mar 19;6(3):e1000815. doi: 10.1371/journal.ppat.1000815 (PMC2841619; doi:10.1371/journal.ppat.1000815)
Supplement: Table S2 — The 48 and 192 highest scoring H-2Kb-restricted octameric epitopes as assayed in screens 01 and 02, respectively. The epitopes were derived from putative secreted proteins of Toxoplasma gondii. Predictions were performed using a consensus epitope prediction algorithm. The program, including documentation for its execution and sample data, is freely available at http://jura.wi.mit.edu/bioc/grotenbreg. The epitope identified to give a CD8+ T cells response in B6 mice is highlighted in red. (0.62 MB DOC) [file ppat.1000815.s004.doc]

**Table II.** The 48 and 192 highest scoring H-2K^b^–restricted octameric epitopes as assayed in screens 01 and 02, respectively. The epitopes were derived from putative secreted proteins of *Toxoplasma gondii*. Predictions were performed using a consensus epitope prediction algorithm. The program, including documentation for its execution and sample data, is freely available at <http://jura.wi.mit.edu/bioc/grotenbreg>. The epitope identified to give a CD8^+^ T cells response in B6 mice is highlighted in red.

| Screen 01 | | | | | | | | | | | |
| --- | --- | --- | --- | --- | --- | --- | --- | --- | --- | --- | --- |
| **Peptide** | **Orf** | **Position** | **arb-**  **score** | **smm-score** | **uda-**  **score** | **park-**  **score** | **arb-**  **rank** | **smm-**  **rank** | **uda-**  **rank** | **park-**  **rank** | **median**  **rank** |
| RFFLYPRM | 4 | 353 | -0.344 | -1.815 | -0.995 | -6.087 | 3 | 1 | 13 | 2 | 2.5 |
| ASLQHYGL | 4 | 398 | -0.397 | -1.399 | -1.392 | -2.398 | 2 | 11 | 2 | 120 | 6.5 |
| ASLHHYGL | 3 | 402 | -0.402 | -1.398 | -1.263 | -2.398 | 1 | 12 | 5 | 141 | 8.5 |
| RHAIFSAL | 2 | 3 | -0.254 | -1.346 | 0.039 | -4.796 | 11 | 16 | 137 | 7 | 13.5 |
| AAFFVSAL | 4 | 18 | -0.271 | -1.149 | -1.22 | -3.186 | 7 | 31 | 7 | 40 | 19 |
| LTLQVIRL | 3 | 393 | -0.241 | -1.188 | -1.594 | -2.493 | 13 | 30 | 1 | 92 | 21.5 |
| SPTGFRRL | 5 | 106 | -0.226 | -1.24 | -0.872 | -4.701 | 19 | 24 | 26 | 13 | 21.5 |
| AAFLVLGL | 5 | 18 | -0.227 | -1.069 | -1.309 | -3.091 | 18 | 38 | 4 | 48 | 28 |
| TVLGFAAL | 2 | 183 | -0.168 | -1.503 | -0.64 | -4.701 | 52 | 5 | 41 | 16 | 28.5 |
| SPSAFRRL | 4 | 106 | -0.22 | -0.913 | -0.651 | -4.701 | 20 | 58 | 38 | 10 | 29 |
| LTLQLIRL | 4 | 389 | -0.216 | -1.115 | -1.003 | -2.493 | 24 | 36 | 12 | 96 | 30 |
| FIFPFDLV | 3 | 327 | -0.233 | -1.292 | -0.402 | -3.178 | 17 | 19 | 58 | 44 | 31.5 |
| VIRLLASL | 3 | 397 | -0.214 | -1.311 | -0.611 | -0.971 | 25 | 17 | 42 | 315 | 33.5 |
| SALCVLGL | 2 | 8 | -0.183 | -1.203 | -1.164 | -2.398 | 40 | 28 | 9 | 143 | 34 |
| RVPLHTRL | 5 | 139 | -0.197 | -0.992 | -1.229 | -3.592 | 37 | 44 | 6 | 31 | 34 |
| LTVQAVRL | 5 | 394 | -0.207 | -1.048 | -0.962 | -2.398 | 28 | 41 | 19 | 137 | 34.5 |
| SYFAADRL | 2 | 153 | -0.288 | -1.243 | -0.309 | -3.091 | 6 | 23 | 77 | 51 | 37 |
| SLVHHARL | 3 | 384 | -0.205 | -0.982 | -0.932 | -2.493 | 30 | 46 | 21 | 90 | 38 |
| LIRLAASL | 4 | 393 | -0.209 | -1.055 | -0.645 | -1.066 | 27 | 40 | 40 | 306 | 40 |
| IPQPVRAL | 3 | 513 | -0.174 | -0.777 | -1.147 | -3.497 | 47 | 87 | 10 | 35 | 41 |
| GVFAAPTL | 6 | 30 | -0.205 | -0.713 | -0.782 | -2.996 | 29 | 115 | 28 | 58 | 43.5 |
| FAFDTWTL | 3 | 473 | -0.153 | -1.057 | 0.064 | -3.871 | 57 | 39 | 146 | 23 | 48 |
| SLQHYGLV | 4 | 399 | -0.149 | -0.607 | -0.698 | -4.19 | 62 | 149 | 37 | 21 | 49.5 |
| AAFLGMGL | 2 | 194 | -0.2 | -0.946 | -0.277 | -3.091 | 36 | 52 | 83 | 50 | 51 |
| LGLVAAAL | 2 | 13 | -0.196 | -1.206 | -0.172 | -2.675 | 38 | 27 | 93 | 64 | 51 |
| FGQHHPTL | 3 | 463 | -0.17 | -0.92 | -0.282 | -3.584 | 50 | 56 | 82 | 33 | 53 |
| FFRRFRRV | 4 | 164 | -0.202 | -0.888 | -0.543 | -0.876 | 34 | 60 | 48 | 346 | 54 |
| VVRVASEL | 5 | 244 | -0.18 | -0.863 | -0.983 | -0.789 | 42 | 69 | 15 | 359 | 55.5 |
| IFAMVTGL | 6 | 320 | -0.268 | -1.136 | -0.284 | -2.493 | 10 | 33 | 81 | 87 | 57 |
| VPLHTRLL | 5 | 140 | -0.146 | -0.686 | -0.747 | -2.996 | 63 | 123 | 31 | 57 | 60 |
| VWLAAAFL | 5 | 14 | -0.219 | -0.876 | -0.403 | -2.398 | 23 | 64 | 57 | 138 | 60.5 |
| CSGEFGKL | 5 | 168 | -0.129 | -0.952 | -0.226 | -4.97 | 75 | 50 | 84 | 6 | 62.5 |
| STLGPVKL | 6 | 196 | -0.095 | -0.926 | -0.351 | -2.58 | 111 | 54 | 67 | 75 | 71 |
| AAAAFLGM | 2 | 192 | -0.13 | -0.853 | 0.32 | -4.701 | 74 | 71 | 224 | 14 | 72.5 |
| VKLLVYKL | 5 | 518 | -0.271 | -0.872 | -0.29 | -1.281 | 8 | 65 | 80 | 246 | 72.5 |
| KAVTLSSL | 6 | 113 | -0.115 | -0.842 | -0.293 | -2.58 | 88 | 75 | 78 | 74 | 76.5 |
| YGLVHTYL | 3 | 407 | -0.125 | -0.952 | -0.114 | -2.58 | 81 | 51 | 108 | 80 | 80.5 |
| FLTGFEHL | 3 | 428 | -0.16 | -0.717 | 0.007 | -4.701 | 54 | 111 | 129 | 11 | 82.5 |
| AVRLVAHL | 5 | 398 | -0.169 | -0.716 | -1.337 | -0.884 | 51 | 114 | 3 | 339 | 82.5 |
| VQLEFAGA | 6 | 297 | 0.273 | -1.42 | -0.146 | -2.58 | 545 | 10 | 100 | 66 | 83 |
| FPFDTWTL | 4 | 469 | -0.126 | -0.775 | 0.446 | -3.871 | 79 | 89 | 269 | 24 | 84 |
| ASLDTAGI | 6 | 134 | -0.161 | -0.794 | -0.195 | -2.164 | 53 | 83 | 88 | 166 | 85.5 |
| RASLFGLL | 1 | 38 | -0.127 | -0.741 | -0.166 | -4.701 | 78 | 103 | 95 | 17 | 86.5 |
| VIDDVQQL | 1 | 164 | -0.18 | -0.984 | 0.015 | -1.24 | 41 | 45 | 132 | 259 | 88.5 |
| LALPAVGM | 2 | 143 | -0.103 | -0.78 | -0.45 | -2.398 | 102 | 86 | 55 | 116 | 94 |
| IEWIYRRC | 4 | 499 | 0.324 | -0.84 | -0.082 | -3.091 | 621 | 76 | 114 | 53 | 95 |
| FIISSGFL | 6 | 8 | -0.143 | -0.76 | 0.282 | -2.485 | 66 | 94 | 209 | 104 | 99 |
| AASVFVCL | 1 | 11 | -0.034 | -0.409 | -0.76 | -4.701 | 171 | 250 | 29 | 8 | 100 |

| Screen 02 | | | | | | | | | | |  |
| --- | --- | --- | --- | --- | --- | --- | --- | --- | --- | --- | --- |
| **peptide** | **Orf** | **Position** | **arb-**  **score** | **smm-score** | **uda-**  **score** | **park-**  **score** | **arb-**  **rank** | **smm-**  **rank** | **uda-**  **rank** | **park-**  **rank** | **median**  **rank** |
| IHYDYANL | 24 | 1049 | -0.37 | -2.762 | -1.95 | -7.462 | 21 | 1 | 6 | 1 | 3.5 |
| IAFAYTTL | 3 | 489 | -0.403 | -2.237 | -1.855 | -6.182 | 8 | 2 | 7 | 16 | 7.5 |
| IGPLYLQL | 69 | 178 | -0.282 | -1.846 | -1.663 | -6.857 | 107 | 13 | 15 | 4 | 14 |
| SVLAFRRL | 5 | 59 | -0.374 | -1.864 | -1.824 | -4.701 | 19 | 12 | 9 | 113 | 15.5 |
| VTPGYSGL | 57 | 176 | -0.304 | -2.075 | -1.474 | -6.493 | 68 | 7 | 30 | 12 | 21 |
| SSQFFGAL | 31 | 1198 | -0.415 | -1.876 | -0.867 | -5.8 | 5 | 11 | 212 | 33 | 22 |
| VVLDYTRL | 4 | 402 | -0.326 | -2.157 | -2.165 | -5.576 | 40 | 5 | 3 | 46 | 22.5 |
| AIVEYARL | 36 | 743 | -0.34 | -2.115 | -1.514 | -5.853 | 33 | 6 | 24 | 28 | 26 |
| VALQYPGL | 7 | 28 | -0.32 | -2.189 | -2.193 | -5.299 | 51 | 3 | 2 | 66 | 27 |
| SSFLSSPL | 16 | 39 | -0.468 | -1.693 | -1.496 | -3.186 | 1 | 31 | 27 | 698 | 29 |
| VSVSFMQL | 1 | 320 | -0.412 | -2.169 | -1.286 | -4.788 | 7 | 4 | 54 | 98 | 30.5 |
| SSVRAVNL | 1 | 297 | -0.382 | -1.815 | -1.324 | -2.762 | 16 | 14 | 50 | 992 | 33 |
| SSMDYESL | 29 | 390 | -0.191 | -1.769 | -1.751 | -5.671 | 421 | 23 | 11 | 44 | 33.5 |
| FVYFFAFL | 3 | 10 | -0.318 | -1.914 | -1.04 | -6.31 | 55 | 10 | 122 | 15 | 35 |
| AGFSFALL | 50 | 3 | -0.325 | -1.734 | -0.808 | -5.671 | 42 | 28 | 253 | 45 | 43.5 |
| ASIRYLPL | 31 | 1385 | -0.205 | -1.619 | -2.416 | -5.576 | 344 | 41 | 1 | 48 | 44.5 |
| INTDYDPL | 61 | 305 | -0.219 | -1.531 | -1.828 | -5.758 | 287 | 69 | 8 | 38 | 53.5 |
| LAQHFRRL | 9 | 129 | -0.303 | -1.387 | -1.359 | -5.8 | 69 | 130 | 43 | 35 | 56 |
| KAYGYSCL | 41 | 324 | -0.071 | -1.526 | -1.381 | -7.003 | 1604 | 73 | 40 | 3 | 56.5 |
| VVVMVSHL | 57 | 10 | -0.375 | -1.501 | -1.456 | -2.398 | 18 | 81 | 33 | 2032 | 57 |
| IAFFLLPL | 60 | 4 | -0.322 | -1.48 | -1.277 | -3.091 | 47 | 90 | 56 | 808 | 73 |
| VSWIHARL | 36 | 787 | -0.294 | -1.533 | -1.72 | -2.398 | 84 | 68 | 13 | 1753 | 76 |
| SILVAPKL | 1 | 451 | -0.332 | -1.587 | -1.12 | -2.762 | 36 | 51 | 102 | 994 | 76.5 |
| ANVAFMPL | 51 | 151 | -0.297 | -1.534 | -1.14 | -4.883 | 77 | 67 | 96 | 79 | 78 |
| SVPVVANL | 3 | 519 | -0.301 | -1.477 | -1.749 | -3.774 | 72 | 91 | 12 | 321 | 81.5 |
| VAVSFMQL | 34 | 293 | -0.305 | -1.793 | -0.957 | -4.788 | 67 | 21 | 165 | 99 | 83 |
| LSTGFASL | 36 | 218 | -0.296 | -1.695 | -1.079 | -4.796 | 81 | 30 | 112 | 93 | 87 |
| ISFASLQL | 60 | 31 | -0.414 | -1.465 | -1.19 | -3.273 | 6 | 97 | 79 | 657 | 88 |
| AFPAFPRL | 57 | 214 | -0.302 | -1.423 | -0.918 | -5.8 | 71 | 114 | 180 | 34 | 92.5 |
| ARYCYRGL | 70 | 154 | -0.131 | -1.36 | -1.983 | -5.799 | 895 | 149 | 5 | 36 | 92.5 |
| RWLKYSTL | 31 | 116 | -0.264 | -1.36 | -1.48 | -5.671 | 145 | 148 | 29 | 43 | 94 |
| SSFPRSAL | 24 | 28 | -0.327 | -1.585 | -0.985 | -0.883 | 39 | 52 | 147 | 4792 | 99.5 |
| VSLSRRRL | 52 | 179 | -0.273 | -1.468 | -1.13 | 0 | 120 | 96 | 99 | 10098 | 109.5 |
| ISQKLKSL | 51 | 10 | -0.315 | -1.402 | -1.139 | -3.679 | 58 | 123 | 97 | 356 | 110 |
| VSFFHQHV | 33 | 471 | -0.291 | -1.377 | -1.283 | -0.693 | 87 | 140 | 55 | 5558 | 113.5 |
| VQAIFSRL | 71 | 685 | -0.282 | -1.597 | -0.884 | -4.701 | 108 | 45 | 197 | 122 | 115 |
| SSPLYATV | 36 | 74 | -0.231 | -1.65 | -1.554 | -4.285 | 242 | 39 | 21 | 194 | 116.5 |
| MPPEFQPL | 59 | 382 | -0.242 | -1.669 | -0.814 | -5.887 | 205 | 34 | 248 | 26 | 119.5 |
| HILPFGSL | 31 | 264 | -0.264 | -1.617 | -0.882 | -4.788 | 144 | 42 | 199 | 96 | 120 |
| RSFGFVPV | 72 | 295 | -0.314 | -1.75 | -0.91 | -3.091 | 60 | 25 | 187 | 792 | 123.5 |
| KTFIFSDL | 46 | 367 | -0.264 | -1.462 | -0.834 | -5.394 | 148 | 99 | 229 | 59 | 123.5 |
| GITEFPGL | 50 | 292 | -0.277 | -1.347 | -0.801 | -4.97 | 116 | 158 | 258 | 78 | 137 |
| INQKQRRL | 52 | 1553 | -0.282 | -1.321 | -1.067 | -3.861 | 106 | 170 | 115 | 299 | 142.5 |
| DSAVFPGL | 29 | 540 | -0.269 | -1.378 | 0.172 | -4.606 | 132 | 138 | 2211 | 149 | 143.5 |
| GSPFFAFL | 40 | 23 | -0.286 | -1.296 | -0.706 | -5.8 | 99 | 194 | 338 | 32 | 146.5 |
| MAFVMPNL | 24 | 1082 | -0.371 | -1.949 | -0.776 | -3.178 | 20 | 8 | 279 | 712 | 149.5 |
| WLPVYVPL | 1 | 2 | -0.223 | -1.022 | -1.437 | -6.398 | 264 | 532 | 35 | 14 | 149.5 |
| ALYRFVKL | 31 | 1027 | -0.156 | -1.216 | -1.316 | -6.674 | 648 | 250 | 51 | 9 | 150.5 |
| AVLLFFNM | 36 | 16 | -0.199 | -1.361 | -0.967 | -4.883 | 374 | 147 | 160 | 83 | 153.5 |
| MTLAFKSM | 31 | 91 | -0.256 | -1.794 | -0.449 | -4.606 | 165 | 20 | 658 | 154 | 159.5 |
| SSLPPAAL | 30 | 99 | -0.242 | -1.474 | -1.058 | -2.493 | 204 | 94 | 117 | 1430 | 160.5 |
| SVYNYYDI | 31 | 1644 | -0.252 | -1.336 | -0.742 | -5.799 | 174 | 166 | 305 | 37 | 170 |
| RSVKWTQL | 25 | 45 | -0.42 | -1.571 | -0.753 | -2.857 | 2 | 56 | 293 | 945 | 174.5 |
| VSNAFTEL | 3 | 448 | -0.227 | -1.44 | -0.854 | -4.701 | 253 | 107 | 219 | 131 | 175 |
| TCFLYSLL | 60 | 50 | -0.21 | -1.091 | -1.468 | -6.087 | 326 | 412 | 31 | 18 | 178.5 |
| FSVQRPPL | 63 | 2 | -0.314 | -1.381 | -0.834 | 0 | 59 | 136 | 228 | 9796 | 182 |
| VSGVFRRV | 56 | 295 | -0.271 | -1.223 | -1.122 | -2.303 | 127 | 243 | 101 | 2139 | 185 |
| SAPKHCGL | 11 | 272 | -0.272 | -1.211 | -1.181 | -3.679 | 126 | 258 | 84 | 387 | 192 |
| YAFLTANL | 34 | 211 | -0.288 | -1.417 | -0.521 | -3.966 | 91 | 118 | 559 | 270 | 194 |
| VSQRASRV | 2 | 71 | -0.364 | -1.27 | -0.922 | -1.376 | 22 | 212 | 178 | 3136 | 195 |
| KSLLALNL | 31 | 674 | -0.24 | -1.315 | -1.111 | -2.485 | 214 | 177 | 105 | 1537 | 195.5 |
| RAFSASSL | 52 | 380 | -0.32 | -1.358 | -0.821 | -3.186 | 52 | 151 | 246 | 691 | 198.5 |
| IAFQVGIL | 3 | 243 | -0.229 | -1.35 | -1.603 | -3.091 | 250 | 155 | 18 | 791 | 202.5 |
| ASPQWKHL | 71 | 103 | -0.262 | -1.215 | -1.356 | -3.497 | 155 | 251 | 44 | 471 | 203 |
| FVPVVPGL | 72 | 299 | -0.28 | -1.18 | -1.528 | -3.402 | 111 | 296 | 23 | 567 | 203.5 |
| RIFSVCAL | 68 | 379 | -0.416 | -1.657 | -0.67 | -3.273 | 4 | 37 | 372 | 647 | 204.5 |
| ITLDPSKL | 63 | 48 | -0.242 | -1.358 | -0.877 | -2.857 | 206 | 152 | 203 | 954 | 204.5 |
| VHYAYQKI | 31 | 964 | -0.168 | -1.497 | -0.712 | -5.886 | 568 | 84 | 331 | 27 | 207.5 |
| RNYGFYYV | 44 | 341 | -0.245 | -1.539 | -0.192 | -4.189 | 194 | 66 | 1113 | 223 | 208.5 |
| MPMVFRHM | 31 | 1352 | -0.224 | -1.421 | -0.617 | -4.606 | 261 | 116 | 434 | 165 | 213 |
| IMFFHPVL | 31 | 289 | -0.249 | -1.22 | -1.406 | -3.091 | 182 | 248 | 38 | 758 | 215 |
| SSLLSLGL | 39 | 8 | -0.301 | -1.297 | -0.826 | -2.398 | 74 | 193 | 240 | 1939 | 216.5 |
| TVILYLAL | 39 | 16 | -0.016 | -1.109 | -1.505 | -5.299 | 2526 | 384 | 26 | 67 | 225.5 |
| AIPVTSLL | 8 | 150 | -0.279 | -0.915 | -0.78 | -4.467 | 112 | 770 | 276 | 178 | 227 |
| KITYFGTL | 37 | 54 | -0.235 | -1.252 | -0.488 | -4.788 | 231 | 225 | 598 | 101 | 228 |
| ITLDATAL | 68 | 518 | -0.221 | -1.445 | -0.915 | -2.675 | 275 | 105 | 182 | 1031 | 228.5 |
| VVQSYQSV | 29 | 971 | -0.225 | -1.264 | -1.35 | -4.095 | 257 | 217 | 45 | 241 | 229 |
| AAVVFSHV | 71 | 175 | -0.288 | -1.236 | -0.835 | -2.493 | 92 | 241 | 226 | 1464 | 233.5 |
| IYSSFYQM | 33 | 194 | -0.38 | -1.104 | -0.216 | -4.883 | 17 | 390 | 1066 | 80 | 235 |
| ASYHYYLS | 35 | 720 | 0.148 | -1.5 | -0.678 | -4.7 | 5475 | 82 | 363 | 146 | 254.5 |
| VAYLSCFL | 8 | 11 | -0.233 | -1.256 | -0.437 | -3.912 | 236 | 224 | 674 | 280 | 258 |
| ISPFPMEL | 44 | 143 | -0.217 | -1.258 | -0.87 | -3.497 | 295 | 222 | 207 | 502 | 258.5 |
| LSQWVSLL | 19 | 72 | -0.394 | -1.023 | -1.023 | -3.592 | 14 | 528 | 131 | 395 | 263 |
| ISIRQRSL | 31 | 1656 | -0.182 | -1.571 | -1.428 | -2.58 | 470 | 57 | 36 | 1207 | 263.5 |
| ASLALTGL | 14 | 10 | -0.326 | -1.386 | -0.645 | -2.493 | 41 | 131 | 398 | 1417 | 264.5 |
| MSLSLTAL | 51 | 141 | -0.316 | -1.813 | -0.589 | -2.398 | 57 | 18 | 472 | 1816 | 264.5 |
| VGVTFIHI | 72 | 317 | -0.238 | -1.529 | 0.019 | -3.861 | 222 | 71 | 1664 | 309 | 265.5 |
| MALTVSLL | 1 | 561 | -0.199 | -1.397 | -0.972 | -2.58 | 378 | 126 | 156 | 1306 | 267 |
| LPYSLSNL | 64 | 618 | -0.206 | -1.317 | -0.567 | -4.284 | 338 | 173 | 498 | 198 | 268 |
| MMGLYALL | 24 | 1211 | -0.207 | -1.261 | -0.732 | -5.394 | 333 | 220 | 317 | 55 | 268.5 |
| HSGGFTPL | 72 | 202 | -0.246 | -1.139 | -0.209 | -4.701 | 190 | 352 | 1080 | 127 | 271 |
| FFFKTISL | 31 | 340 | -0.358 | -1.194 | -0.509 | -3.966 | 24 | 272 | 569 | 271 | 271.5 |
| QAWPFPQL | 3 | 304 | -0.181 | -1.065 | -1.258 | -4.788 | 476 | 448 | 60 | 95 | 271.5 |
| MVVRASFL | 41 | 14 | -0.363 | -1.503 | -0.592 | -2.58 | 23 | 79 | 468 | 1280 | 273.5 |
| EVTPYGNL | 72 | 328 | -0.177 | -1.038 | -1.637 | -5.481 | 506 | 500 | 16 | 52 | 276 |
| WVPNYFLL | 63 | 348 | -0.178 | -1.026 | -1.253 | -6.398 | 493 | 521 | 61 | 13 | 277 |
| IHYDYANL | 24 | 1049 | -0.37 | -2.762 | -1.95 | -7.462 | 21 | 1 | 6 | 1 | 3.5 |
| IAFAYTTL | 3 | 489 | -0.403 | -2.237 | -1.855 | -6.182 | 8 | 2 | 7 | 16 | 7.5 |
| IGPLYLQL | 69 | 178 | -0.282 | -1.846 | -1.663 | -6.857 | 107 | 13 | 15 | 4 | 14 |
| SVLAFRRL | 5 | 59 | -0.374 | -1.864 | -1.824 | -4.701 | 19 | 12 | 9 | 113 | 15.5 |
| VTPGYSGL | 57 | 176 | -0.304 | -2.075 | -1.474 | -6.493 | 68 | 7 | 30 | 12 | 21 |
| SSQFFGAL | 31 | 1198 | -0.415 | -1.876 | -0.867 | -5.8 | 5 | 11 | 212 | 33 | 22 |
| VVLDYTRL | 4 | 402 | -0.326 | -2.157 | -2.165 | -5.576 | 40 | 5 | 3 | 46 | 22.5 |
| AIVEYARL | 36 | 743 | -0.34 | -2.115 | -1.514 | -5.853 | 33 | 6 | 24 | 28 | 26 |
| VALQYPGL | 7 | 28 | -0.32 | -2.189 | -2.193 | -5.299 | 51 | 3 | 2 | 66 | 27 |
| SSFLSSPL | 16 | 39 | -0.468 | -1.693 | -1.496 | -3.186 | 1 | 31 | 27 | 698 | 29 |
| VSVSFMQL | 1 | 320 | -0.412 | -2.169 | -1.286 | -4.788 | 7 | 4 | 54 | 98 | 30.5 |
| SSVRAVNL | 1 | 297 | -0.382 | -1.815 | -1.324 | -2.762 | 16 | 14 | 50 | 992 | 33 |
| SSMDYESL | 29 | 390 | -0.191 | -1.769 | -1.751 | -5.671 | 421 | 23 | 11 | 44 | 33.5 |
| FVYFFAFL | 3 | 10 | -0.318 | -1.914 | -1.04 | -6.31 | 55 | 10 | 122 | 15 | 35 |
| AGFSFALL | 50 | 3 | -0.325 | -1.734 | -0.808 | -5.671 | 42 | 28 | 253 | 45 | 43.5 |
| ASIRYLPL | 31 | 1385 | -0.205 | -1.619 | -2.416 | -5.576 | 344 | 41 | 1 | 48 | 44.5 |
| INTDYDPL | 61 | 305 | -0.219 | -1.531 | -1.828 | -5.758 | 287 | 69 | 8 | 38 | 53.5 |
| LAQHFRRL | 9 | 129 | -0.303 | -1.387 | -1.359 | -5.8 | 69 | 130 | 43 | 35 | 56 |
| KAYGYSCL | 41 | 324 | -0.071 | -1.526 | -1.381 | -7.003 | 1604 | 73 | 40 | 3 | 56.5 |
| VVVMVSHL | 57 | 10 | -0.375 | -1.501 | -1.456 | -2.398 | 18 | 81 | 33 | 2032 | 57 |
| IAFFLLPL | 60 | 4 | -0.322 | -1.48 | -1.277 | -3.091 | 47 | 90 | 56 | 808 | 73 |
| VSWIHARL | 36 | 787 | -0.294 | -1.533 | -1.72 | -2.398 | 84 | 68 | 13 | 1753 | 76 |
| SILVAPKL | 1 | 451 | -0.332 | -1.587 | -1.12 | -2.762 | 36 | 51 | 102 | 994 | 76.5 |
| ANVAFMPL | 51 | 151 | -0.297 | -1.534 | -1.14 | -4.883 | 77 | 67 | 96 | 79 | 78 |
| SVPVVANL | 3 | 519 | -0.301 | -1.477 | -1.749 | -3.774 | 72 | 91 | 12 | 321 | 81.5 |
| VAVSFMQL | 34 | 293 | -0.305 | -1.793 | -0.957 | -4.788 | 67 | 21 | 165 | 99 | 83 |
| LSTGFASL | 36 | 218 | -0.296 | -1.695 | -1.079 | -4.796 | 81 | 30 | 112 | 93 | 87 |
| ISFASLQL | 60 | 31 | -0.414 | -1.465 | -1.19 | -3.273 | 6 | 97 | 79 | 657 | 88 |
| AFPAFPRL | 57 | 214 | -0.302 | -1.423 | -0.918 | -5.8 | 71 | 114 | 180 | 34 | 92.5 |
| ARYCYRGL | 70 | 154 | -0.131 | -1.36 | -1.983 | -5.799 | 895 | 149 | 5 | 36 | 92.5 |
| RWLKYSTL | 31 | 116 | -0.264 | -1.36 | -1.48 | -5.671 | 145 | 148 | 29 | 43 | 94 |
| SSFPRSAL | 24 | 28 | -0.327 | -1.585 | -0.985 | -0.883 | 39 | 52 | 147 | 4792 | 99.5 |
| VSLSRRRL | 52 | 179 | -0.273 | -1.468 | -1.13 | 0 | 120 | 96 | 99 | 10098 | 109.5 |
| ISQKLKSL | 51 | 10 | -0.315 | -1.402 | -1.139 | -3.679 | 58 | 123 | 97 | 356 | 110 |
| VSFFHQHV | 33 | 471 | -0.291 | -1.377 | -1.283 | -0.693 | 87 | 140 | 55 | 5558 | 113.5 |
| VQAIFSRL | 71 | 685 | -0.282 | -1.597 | -0.884 | -4.701 | 108 | 45 | 197 | 122 | 115 |
| SSPLYATV | 36 | 74 | -0.231 | -1.65 | -1.554 | -4.285 | 242 | 39 | 21 | 194 | 116.5 |
| MPPEFQPL | 59 | 382 | -0.242 | -1.669 | -0.814 | -5.887 | 205 | 34 | 248 | 26 | 119.5 |
| HILPFGSL | 31 | 264 | -0.264 | -1.617 | -0.882 | -4.788 | 144 | 42 | 199 | 96 | 120 |
| RSFGFVPV | 72 | 295 | -0.314 | -1.75 | -0.91 | -3.091 | 60 | 25 | 187 | 792 | 123.5 |
| KTFIFSDL | 46 | 367 | -0.264 | -1.462 | -0.834 | -5.394 | 148 | 99 | 229 | 59 | 123.5 |
| GITEFPGL | 50 | 292 | -0.277 | -1.347 | -0.801 | -4.97 | 116 | 158 | 258 | 78 | 137 |
| INQKQRRL | 52 | 1553 | -0.282 | -1.321 | -1.067 | -3.861 | 106 | 170 | 115 | 299 | 142.5 |
| DSAVFPGL | 29 | 540 | -0.269 | -1.378 | 0.172 | -4.606 | 132 | 138 | 2211 | 149 | 143.5 |
| GSPFFAFL | 40 | 23 | -0.286 | -1.296 | -0.706 | -5.8 | 99 | 194 | 338 | 32 | 146.5 |
| MAFVMPNL | 24 | 1082 | -0.371 | -1.949 | -0.776 | -3.178 | 20 | 8 | 279 | 712 | 149.5 |
| WLPVYVPL | 1 | 2 | -0.223 | -1.022 | -1.437 | -6.398 | 264 | 532 | 35 | 14 | 149.5 |
| ALYRFVKL | 31 | 1027 | -0.156 | -1.216 | -1.316 | -6.674 | 648 | 250 | 51 | 9 | 150.5 |
| AVLLFFNM | 36 | 16 | -0.199 | -1.361 | -0.967 | -4.883 | 374 | 147 | 160 | 83 | 153.5 |
| MTLAFKSM | 31 | 91 | -0.256 | -1.794 | -0.449 | -4.606 | 165 | 20 | 658 | 154 | 159.5 |
| SSLPPAAL | 30 | 99 | -0.242 | -1.474 | -1.058 | -2.493 | 204 | 94 | 117 | 1430 | 160.5 |
| SVYNYYDI | 31 | 1644 | -0.252 | -1.336 | -0.742 | -5.799 | 174 | 166 | 305 | 37 | 170 |
| RSVKWTQL | 25 | 45 | -0.42 | -1.571 | -0.753 | -2.857 | 2 | 56 | 293 | 945 | 174.5 |
| VSNAFTEL | 3 | 448 | -0.227 | -1.44 | -0.854 | -4.701 | 253 | 107 | 219 | 131 | 175 |
| TCFLYSLL | 60 | 50 | -0.21 | -1.091 | -1.468 | -6.087 | 326 | 412 | 31 | 18 | 178.5 |
| FSVQRPPL | 63 | 2 | -0.314 | -1.381 | -0.834 | 0 | 59 | 136 | 228 | 9796 | 182 |
| VSGVFRRV | 56 | 295 | -0.271 | -1.223 | -1.122 | -2.303 | 127 | 243 | 101 | 2139 | 185 |
| SAPKHCGL | 11 | 272 | -0.272 | -1.211 | -1.181 | -3.679 | 126 | 258 | 84 | 387 | 192 |
| YAFLTANL | 34 | 211 | -0.288 | -1.417 | -0.521 | -3.966 | 91 | 118 | 559 | 270 | 194 |
| VSQRASRV | 2 | 71 | -0.364 | -1.27 | -0.922 | -1.376 | 22 | 212 | 178 | 3136 | 195 |
| KSLLALNL | 31 | 674 | -0.24 | -1.315 | -1.111 | -2.485 | 214 | 177 | 105 | 1537 | 195.5 |
| RAFSASSL | 52 | 380 | -0.32 | -1.358 | -0.821 | -3.186 | 52 | 151 | 246 | 691 | 198.5 |
| IAFQVGIL | 3 | 243 | -0.229 | -1.35 | -1.603 | -3.091 | 250 | 155 | 18 | 791 | 202.5 |
| ASPQWKHL | 71 | 103 | -0.262 | -1.215 | -1.356 | -3.497 | 155 | 251 | 44 | 471 | 203 |
| FVPVVPGL | 72 | 299 | -0.28 | -1.18 | -1.528 | -3.402 | 111 | 296 | 23 | 567 | 203.5 |
| RIFSVCAL | 68 | 379 | -0.416 | -1.657 | -0.67 | -3.273 | 4 | 37 | 372 | 647 | 204.5 |
| ITLDPSKL | 63 | 48 | -0.242 | -1.358 | -0.877 | -2.857 | 206 | 152 | 203 | 954 | 204.5 |
| VHYAYQKI | 31 | 964 | -0.168 | -1.497 | -0.712 | -5.886 | 568 | 84 | 331 | 27 | 207.5 |
| RNYGFYYV | 44 | 341 | -0.245 | -1.539 | -0.192 | -4.189 | 194 | 66 | 1113 | 223 | 208.5 |
| MPMVFRHM | 31 | 1352 | -0.224 | -1.421 | -0.617 | -4.606 | 261 | 116 | 434 | 165 | 213 |
| IMFFHPVL | 31 | 289 | -0.249 | -1.22 | -1.406 | -3.091 | 182 | 248 | 38 | 758 | 215 |
| SSLLSLGL | 39 | 8 | -0.301 | -1.297 | -0.826 | -2.398 | 74 | 193 | 240 | 1939 | 216.5 |
| TVILYLAL | 39 | 16 | -0.016 | -1.109 | -1.505 | -5.299 | 2526 | 384 | 26 | 67 | 225.5 |
| AIPVTSLL | 8 | 150 | -0.279 | -0.915 | -0.78 | -4.467 | 112 | 770 | 276 | 178 | 227 |
| KITYFGTL | 37 | 54 | -0.235 | -1.252 | -0.488 | -4.788 | 231 | 225 | 598 | 101 | 228 |
| ITLDATAL | 68 | 518 | -0.221 | -1.445 | -0.915 | -2.675 | 275 | 105 | 182 | 1031 | 228.5 |
| VVQSYQSV | 29 | 971 | -0.225 | -1.264 | -1.35 | -4.095 | 257 | 217 | 45 | 241 | 229 |
| AAVVFSHV | 71 | 175 | -0.288 | -1.236 | -0.835 | -2.493 | 92 | 241 | 226 | 1464 | 233.5 |
| IYSSFYQM | 33 | 194 | -0.38 | -1.104 | -0.216 | -4.883 | 17 | 390 | 1066 | 80 | 235 |
| ASYHYYLS | 35 | 720 | 0.148 | -1.5 | -0.678 | -4.7 | 5475 | 82 | 363 | 146 | 254.5 |
| VAYLSCFL | 8 | 11 | -0.233 | -1.256 | -0.437 | -3.912 | 236 | 224 | 674 | 280 | 258 |
| ISPFPMEL | 44 | 143 | -0.217 | -1.258 | -0.87 | -3.497 | 295 | 222 | 207 | 502 | 258.5 |
| LSQWVSLL | 19 | 72 | -0.394 | -1.023 | -1.023 | -3.592 | 14 | 528 | 131 | 395 | 263 |
| ISIRQRSL | 31 | 1656 | -0.182 | -1.571 | -1.428 | -2.58 | 470 | 57 | 36 | 1207 | 263.5 |
| ASLALTGL | 14 | 10 | -0.326 | -1.386 | -0.645 | -2.493 | 41 | 131 | 398 | 1417 | 264.5 |
| MSLSLTAL | 51 | 141 | -0.316 | -1.813 | -0.589 | -2.398 | 57 | 18 | 472 | 1816 | 264.5 |
| VGVTFIHI | 72 | 317 | -0.238 | -1.529 | 0.019 | -3.861 | 222 | 71 | 1664 | 309 | 265.5 |
| MALTVSLL | 1 | 561 | -0.199 | -1.397 | -0.972 | -2.58 | 378 | 126 | 156 | 1306 | 267 |
| LPYSLSNL | 64 | 618 | -0.206 | -1.317 | -0.567 | -4.284 | 338 | 173 | 498 | 198 | 268 |
| MMGLYALL | 24 | 1211 | -0.207 | -1.261 | -0.732 | -5.394 | 333 | 220 | 317 | 55 | 268.5 |
| HSGGFTPL | 72 | 202 | -0.246 | -1.139 | -0.209 | -4.701 | 190 | 352 | 1080 | 127 | 271 |
| FFFKTISL | 31 | 340 | -0.358 | -1.194 | -0.509 | -3.966 | 24 | 272 | 569 | 271 | 271.5 |
| QAWPFPQL | 3 | 304 | -0.181 | -1.065 | -1.258 | -4.788 | 476 | 448 | 60 | 95 | 271.5 |
| MVVRASFL | 41 | 14 | -0.363 | -1.503 | -0.592 | -2.58 | 23 | 79 | 468 | 1280 | 273.5 |
| EVTPYGNL | 72 | 328 | -0.177 | -1.038 | -1.637 | -5.481 | 506 | 500 | 16 | 52 | 276 |
| WVPNYFLL | 63 | 348 | -0.178 | -1.026 | -1.253 | -6.398 | 493 | 521 | 61 | 13 | 277 |
