# Supplementary material for: Differential Regulation of Effector- and Central-Memory Responses to Toxoplasma gondii Infection by IL-12 Revealed by Tracking of Tgd057-Specific CD8+ T Cells
Source: PLoS Pathog. 2010 Mar 19;6(3):e1000815. doi: 10.1371/journal.ppat.1000815 (PMC2841619; doi:10.1371/journal.ppat.1000815)
Supplement: Table S3 — The 48 and 192 highest scoring H-2Db-restricted nonameric epitopes as assayed in screens 01 and 02, respectively. The epitopes were derived from putative secreted proteins of Toxoplasma gondii. Predictions were performed using a consensus epitope prediction algorithm. The program, including documentation for its execution and sample data, is freely available at http://jura.wi.mit.edu/bioc/grotenbreg. (0.63 MB DOC) [file ppat.1000815.s005.doc]

**Table III.** The 48 and 192 highest scoring H-2D^b^–restricted nonameric epitopes as assayed in screens 01 and 02, respectively. The epitopes were derived from putative secreted proteins of *Toxoplasma gondii*. Predictions were performed using a consensus epitope prediction algorithm. The program, including documentation for its execution and sample data, is freely available at <http://jura.wi.mit.edu/bioc/grotenbreg>.

| Screen 01 | | | | | | | | | | | | | | | |
| --- | --- | --- | --- | --- | --- | --- | --- | --- | --- | --- | --- | --- | --- | --- | --- |
| **peptide** | **Orf** | **Position** | **arb-**  **score** | **smm-score** | **uda-**  **score** | **park-**  **score** | **arb-**  **rank** | **smm-**  **rank** | | **uda-**  **rank** | | **park-**  **rank** | | | **median**  **rank** |
| ASSDNGATL | 6 | 259 | -0.325 | -0.095 | -1.149 | -3.456 | 12 | 70 | | 13 | | 6 | | | 12.5 |
| RVLSNANHL | 4 | 220 | -0.05 | -0.527 | -1.462 | -3.778 | 95 | 25 | | 5 | | 3 | | | 15 |
| RMQSNLHIL | 4 | 359 | -0.256 | -0.434 | -1.565 | -3.494 | 28 | 28 | | 2 | | 5 | | | 16.5 |
| VASVVQDEM | 1 | 154 | -0.521 | -0.848 | -0.734 | -1.535 | 1 | 5 | | 39 | | 107 | | | 22 |
| TALVGHTVL | 5 | 285 | 0.145 | -0.662 | -1.502 | -2.012 | 280 | 13 | | 4 | | 32 | | | 22.5 |
| RNVHNPFSL | 5 | 177 | -0.182 | -0.081 | -1.553 | -3.778 | 45 | 72 | | 3 | | 4 | | | 24.5 |
| ASHVSIFAM | 6 | 315 | -0.413 | -0.257 | -1.652 | -1.535 | 4 | 46 | | 1 | | 103 | | | 25 |
| AAPTLMSFL | 6 | 33 | -0.368 | -0.072 | -1.295 | -1.933 | 8 | 76 | | 9 | | 44 | | | 26.5 |
| ASVRSSSCL | 4 | 5 | -0.315 | 0.026 | -1.081 | -1.933 | 15 | 93 | | 15 | | 41 | | | 28 |
| RALNKGETV | 1 | 118 | -0.235 | -0.576 | -0.841 | 0.409 | 30 | 21 | | 31 | | 1020 | | | 30.5 |
| AALGGSEWI | 3 | 498 | -0.033 | -0.687 | -0.97 | -1.933 | 103 | 11 | | 24 | | 40 | | | 32 |
| FSLKVSPDL | 5 | 183 | -0.209 | -0.581 | -0.632 | -2.012 | 35 | 20 | | 46 | | 30 | | | 32.5 |
| AGIKLTVPI | 6 | 139 | -0.158 | 0.707 | -0.944 | -2.012 | 51 | 262 | | 26 | | 29 | | | 40 |
| KSLVHHARL | 4 | 379 | -0.209 | -0.109 | -0.619 | -1.971 | 36 | 66 | | 48 | | 36 | | | 42 |
| GWVKNLLFL | 5 | 353 | -0.151 | -0.402 | -0.363 | -3.936 | 55 | 29 | | 95 | | 1 | | | 42 |
| FQVRNILLD | 2 | 410 | -0.09 | -0.871 | 1.475 | -2.518 | 76 | 4 | | 740 | | 13 | | | 44.5 |
| SALCVLGLV | 4 | 8 | -0.279 | -0.698 | -0.471 | -0.711 | 23 | 10 | | 66 | | 338 | | | 44.5 |
| SNLQTFGEV | 3 | 366 | -0.207 | -0.328 | -0.608 | -0.676 | 39 | 36 | | 51 | | 345 | | | 45 |
| EMMGNTYRV | 1 | 62 | -0.349 | -0.055 | 0.86 | -2.114 | 11 | 79 | | 471 | | 20 | | | 49.5 |
| KAVTLSSLI | 6 | 113 | -0.426 | -0.251 | -0.361 | -1.892 | 3 | 49 | | 96 | | 60 | | | 54.5 |
| ASLQHYGLV | 4 | 398 | -0.174 | -0.132 | -0.635 | -0.711 | 47 | 64 | | 44 | | 339 | | | 55.5 |
| VGNVNVEEV | 1 | 94 | -0.036 | 0.507 | -1.34 | -2.234 | 100 | 194 | | 7 | | 18 | | | 59 |
| QAVRLVAHL | 5 | 397 | -0.267 | -0.107 | -0.522 | -1.892 | 26 | 68 | | 59 | | 59 | | | 59 |
| AGVFAAPTL | 6 | 29 | 0.143 | -0.182 | -0.483 | -1.933 | 277 | 55 | | 64 | | 46 | | | 59.5 |
| GANSTLGPV | 6 | 193 | -0.134 | -0.623 | -0.424 | -0.193 | 58 | 15 | | 75 | | 546 | | | 66.5 |
| RGIKNQRQA | 4 | 309 | 0.238 | 0.156 | -0.958 | -2.67 | 411 | 110 | | 25 | | 11 | | | 67.5 |
| AAAAAFLGM | 2 | 191 | -0.211 | -0.602 | -0.202 | -1.535 | 34 | 17 | | 127 | | 104 | | | 69 |
| ASLHHYGLV | 3 | 402 | -0.134 | -0.04 | -0.588 | -0.632 | 59 | 82 | | 55 | | 367 | | | 70.5 |
| HAIFSALCV | 2 | 4 | -0.505 | 0.165 | -0.771 | -0.591 | 2 | 112 | | 36 | | 405 | | | 74 |
| RGIKNQKQA | 3 | 313 | 0.243 | 0.191 | -0.842 | -2.67 | 421 | 118 | | 30 | | 10 | | | 74 |
| TALTEPPTL | 6 | 85 | -0.03 | -0.255 | -0.083 | -1.933 | 106 | 48 | | 142 | | 42 | | | 77 |
| AALAAAAAF | 2 | 188 | 0.014 | -0.647 | -1.221 | 0.067 | 141 | 14 | | 11 | | 837 | | | 77.5 |
| RVPENVKLL | 5 | 513 | 0.03 | 0.307 | -1.147 | -3.857 | 153 | 141 | | 14 | | 2 | | | 77.5 |
| AAAFFVSAL | 3 | 17 | -0.29 | 0.143 | -0.605 | -1.456 | 19 | 105 | | 52 | | 130 | | | 78.5 |
| WTLGSVIFL | 5 | 479 | -0.099 | 0.952 | -0.463 | -1.778 | 71 | 381 | | 68 | | 86 | | | 78.5 |
| NQYCSGTTL | 6 | 227 | -0.273 | -0.122 | -0.375 | -1.342 | 24 | 65 | | 93 | | 206 | | | 79 |
| AYLRFIFPI | 4 | 319 | -0.068 | 0.541 | -0.75 | -1.819 | 87 | 208 | | 37 | | 73 | | | 80 |
| AATASDDEL | 2 | 26 | -0.388 | 0.277 | -0.865 | -1.456 | 7 | 133 | | 29 | | 136 | | | 81 |
| QVIRLLASL | 3 | 396 | -0.196 | 1.063 | -0.371 | -1.857 | 41 | 440 | | 94 | | 68 | | | 81 |
| RMQSNLQTF | 3 | 363 | -0.051 | -0.083 | -1.23 | -1.494 | 93 | 71 | | 10 | | 117 | | | 82 |
| MSVSLHHFI | 6 | 1 | -0.031 | 0.224 | -0.503 | -1.892 | 104 | 125 | | 61 | | 61 | | | 82.5 |
| GQPSCLVWL | 5 | 8 | -0.035 | 0.507 | -0.643 | -1.857 | 101 | 193 | | 43 | | 70 | | | 85.5 |
| SSVVNNVAR | 6 | 181 | -0.097 | -0.176 | 0.668 | -1.711 | 73 | 58 | | 387 | | 100 | | | 86.5 |
| QLIRLAASL | 4 | 392 | -0.183 | 1.627 | -0.412 | -1.778 | 44 | 835 | | 77 | | 98 | | | 87.5 |
| GAMASDPPL | 6 | 44 | -0.268 | 0.468 | -1.427 | -1.415 | 25 | 183 | | 6 | | 151 | | | 88 |
| TPTENHFTL | 6 | 72 | -0.015 | 0.911 | -0.48 | -3.421 | 115 | 358 | | 65 | | 8 | | | 90 |
| GVVRVASEL | 5 | 243 | -0.068 | -0.061 | 0.106 | -1.778 | 88 | 78 | | 193 | | 93 | | | 90.5 |
| SRIRNSDFF | 2 | 36 | 0.026 | 0.199 | -0.564 | -1.819 | 150 | 120 | | 56 | | 71 | | | 95.5 |
| Screen 02 | | | | | | | | | | | | | | | |
| **peptide** | **Orf** | **Position** | **arb-**  **score** | **smm-score** | **uda-**  **score** | **park-**  **score** | **arb-**  **rank** | | **smm-**  **rank** | | **uda-**  **rank** | | **park-**  **rank** | **median**  **rank** | |
| VMIVNVLAL | 6 | 11 | -0.571 | -1.571 | -2.474 | -4.012 | 14 | | 16 | | 1 | | 1 | 7.5 | |
| KAILNFVTV | 32 | 301 | -0.666 | -1.673 | -1.992 | -2.67 | 1 | | 11 | | 13 | | 159 | 12 | |
| IALYNRGHL | 22 | 134 | -0.315 | -1.522 | -1.967 | -3.933 | 227 | | 18 | | 15 | | 8 | 16.5 | |
| YALLNVADI | 24 | 1215 | -0.425 | -0.994 | -2.402 | -3.892 | 68 | | 88 | | 2 | | 14 | 41 | |
| LAFLNYLTI | 3 | 255 | -0.414 | -1.316 | -1.565 | -3.415 | 78 | | 36 | | 46 | | 69 | 57.5 | |
| AQLSTFLEL | 43 | 43 | -0.427 | -1.493 | -1.326 | -1.898 | 65 | | 20 | | 89 | | 633 | 77 | |
| AAVVFSHVL | 71 | 175 | -0.4 | -0.907 | -1.491 | -2.012 | 89 | | 104 | | 53 | | 354 | 96.5 | |
| FALFFVFAL | 65 | 26 | -0.441 | -0.807 | -1.766 | -1.933 | 58 | | 139 | | 25 | | 553 | 98.5 | |
| NKLMNALPM | 71 | 370 | -0.344 | -0.763 | -1.641 | -3.819 | 172 | | 162 | | 38 | | 24 | 100 | |
| SGVVNQGPV | 2 | 27 | -0.446 | -0.706 | -1.802 | -2.711 | 55 | | 197 | | 19 | | 150 | 102.5 | |
| LAVLNVALV | 63 | 28 | -0.37 | -0.979 | -1.354 | -2.591 | 123 | | 90 | | 76 | | 176 | 106.5 | |
| IQLLTLVAM | 51 | 199 | -0.52 | -2.05 | -1.061 | -1.898 | 21 | | 3 | | 205 | | 656 | 113 | |
| AAITKPLTI | 67 | 202 | -0.518 | -1.193 | -1.134 | -0.933 | 22 | | 52 | | 176 | | 3662 | 114 | |
| FSVKNSVFA | 65 | 19 | -0.017 | -0.964 | -1.678 | -2.711 | 1798 | | 93 | | 31 | | 149 | 121 | |
| VGLTNRSTL | 58 | 368 | -0.209 | -0.635 | -2.229 | -3.933 | 560 | | 247 | | 4 | | 7 | 127 | |
| ASWNNSYTL | 68 | 73 | -0.354 | -0.766 | -1.22 | -3.456 | 143 | | 160 | | 129 | | 46 | 136 | |
| DAGANALFI | 72 | 222 | -0.417 | -0.698 | -0.973 | -3.415 | 75 | | 199 | | 279 | | 60 | 137 | |
| AVLHNLRQL | 24 | 193 | -0.236 | -0.747 | -1.288 | -3.898 | 465 | | 172 | | 104 | | 11 | 138 | |
| LAILNEFFA | 16 | 89 | 0.011 | -0.834 | -1.528 | -2.591 | 2110 | | 120 | | 50 | | 179 | 149.5 | |
| RAPENVKLL | 23 | 511 | -0.194 | -0.544 | -1.735 | -3.971 | 635 | | 304 | | 29 | | 4 | 166.5 | |
| KTVVNLTEV | 62 | 255 | -0.431 | -0.725 | -0.753 | -2.635 | 63 | | 186 | | 510 | | 163 | 174.5 | |
| DQPGNHQAL | 18 | 30 | -0.344 | -0.435 | -1.118 | -3.778 | 169 | | 377 | | 183 | | 37 | 176 | |
| VAAGNPSAV | 16 | 55 | -0.448 | -0.474 | -1.28 | -2.155 | 52 | | 351 | | 105 | | 254 | 179.5 | |
| ASSDNGATL | 12 | 242 | -0.325 | -0.095 | -1.149 | -3.456 | 201 | | 866 | | 168 | | 47 | 184.5 | |
| ASVAMLTCM | 3 | 404 | -0.589 | -2.513 | -0.512 | -2.012 | 10 | | 1 | | 870 | | 368 | 189 | |
| TSKKNLKAM | 24 | 1138 | -0.38 | -0.603 | -0.648 | -3.091 | 107 | | 267 | | 647 | | 115 | 191 | |
| SSVYNYYDI | 31 | 1643 | -0.283 | -1.01 | -0.889 | -3.933 | 308 | | 83 | | 358 | | 6 | 195.5 | |
| QALGNSERL | 63 | 216 | -0.28 | -0.821 | -0.985 | -3.892 | 313 | | 128 | | 263 | | 13 | 195.5 | |
| GTVTNPLAI | 59 | 86 | -0.358 | -0.613 | -0.753 | -3.778 | 138 | | 261 | | 509 | | 28 | 199.5 | |
| FAVKHCLLV | 2 | 2 | -0.335 | -0.801 | -1.052 | -0.711 | 186 | | 142 | | 214 | | 4506 | 200 | |
| NSAENTWFI | 68 | 265 | -0.338 | -0.458 | -1.046 | -3.535 | 182 | | 360 | | 219 | | 40 | 200.5 | |
| IALAKLHEL | 22 | 285 | -0.315 | -0.738 | -1.467 | -1.012 | 226 | | 178 | | 59 | | 3554 | 202 | |
| AMLTCMAFV | 3 | 407 | -0.617 | -0.666 | -1.127 | -0.632 | 5 | | 227 | | 181 | | 4960 | 204 | |
| NAENNSLTI | 62 | 218 | -0.273 | -0.794 | -0.981 | -2.757 | 332 | | 144 | | 270 | | 141 | 207 | |
| SQIRTRTEV | 35 | 290 | -0.332 | -1.446 | -1.003 | -0.518 | 190 | | 26 | | 248 | | 6016 | 219 | |
| FMVPSDVVM | 63 | 322 | -0.452 | -0.877 | -0.913 | -1.933 | 50 | | 108 | | 335 | | 501 | 221.5 | |
| SAQDNYSFL | 36 | 812 | -0.261 | -0.492 | -1.258 | -3.456 | 371 | | 338 | | 115 | | 48 | 226.5 | |
| AAVQTGRDL | 36 | 478 | -0.401 | -1.62 | -0.18 | -2.012 | 88 | | 15 | | 1650 | | 367 | 227.5 | |
| RAAVTGHPV | 6 | 65 | -0.38 | -1.118 | -0.886 | -0.193 | 106 | | 67 | | 359 | | 7333 | 232.5 | |
| QAVQVLAQL | 32 | 445 | -0.349 | -0.853 | -0.102 | -2.05 | 161 | | 115 | | 1892 | | 305 | 233 | |
| SKNANFIEI | 37 | 227 | -0.259 | 0.485 | -1.33 | -3.421 | 382 | | 2509 | | 87 | | 55 | 234.5 | |
| TTLTNAALI | 3 | 494 | -0.361 | -0.077 | -0.897 | -3.819 | 134 | | 902 | | 350 | | 26 | 242 | |
| MAPYLMLPM | 24 | 1277 | -0.396 | -0.424 | -1.562 | -1.892 | 92 | | 393 | | 47 | | 688 | 242.5 | |
| AAAATLIAL | 53 | 233 | -0.635 | -1.417 | -0.788 | -1.535 | 3 | | 28 | | 459 | | 1406 | 243.5 | |
| AQLKKVGAM | 26 | 234 | -0.555 | -1.271 | -0.796 | -0.898 | 18 | | 41 | | 449 | | 3701 | 245 | |
| SNLVSLGAM | 61 | 241 | -0.436 | 0.29 | -1.378 | -1.977 | 59 | | 1813 | | 71 | | 421 | 246 | |
| LAPQMPTPV | 71 | 422 | -0.259 | -1.81 | -1.235 | -0.67 | 381 | | 6 | | 122 | | 4687 | 251.5 | |
| NVIVNDGAL | 71 | 288 | -0.316 | -0.582 | -0.852 | -3.898 | 225 | | 281 | | 395 | | 10 | 253 | |
| AAPTQLTPL | 71 | 512 | -0.352 | 0.377 | -1.812 | -2.012 | 147 | | 2109 | | 18 | | 361 | 254 | |
| ATARNQGFL | 24 | 1039 | -0.512 | -0.235 | -0.818 | -3.342 | 27 | | 616 | | 423 | | 86 | 254.5 | |
| LAVVVAAVL | 7 | 14 | -0.464 | -1.075 | -0.636 | -1.971 | 42 | | 73 | | 664 | | 438 | 255.5 | |
| AAVLLLITV | 26 | 10 | -0.561 | -0.749 | -0.903 | -0.711 | 16 | | 171 | | 342 | | 4540 | 256.5 | |
| RSVQVSGAL | 29 | 203 | -0.434 | -1.069 | -0.29 | -1.971 | 60 | | 76 | | 1369 | | 449 | 262.5 | |
| AALQTVHQL | 26 | 89 | -0.288 | -1.803 | -1.013 | -2.012 | 287 | | 7 | | 239 | | 363 | 263 | |
| GSVVNIPDI | 58 | 98 | -0.139 | -0.443 | -1.154 | -3.971 | 900 | | 371 | | 165 | | 3 | 268 | |
| FSVVNTHGA | 3 | 541 | 0.031 | -0.398 | -1.263 | -2.711 | 2335 | | 409 | | 112 | | 147 | 278 | |
| SGIAMQQVL | 15 | 183 | -0.32 | -1.064 | -0.902 | -1.933 | 213 | | 78 | | 345 | | 534 | 279 | |
| VMITLLFQL | 31 | 367 | -0.319 | -0.06 | -1.077 | -2.012 | 215 | | 926 | | 198 | | 350 | 282.5 | |
| MQIPNCEKI | 34 | 401 | -0.254 | -0.749 | -0.503 | -3.778 | 398 | | 170 | | 890 | | 35 | 284 | |
| YGNLNNNVM | 72 | 332 | -0.222 | 0.061 | -1.49 | -3.415 | 516 | | 1179 | | 54 | | 57 | 286.5 | |
| TLPENKATV | 64 | 470 | -0.252 | 0.501 | -1.337 | -2.597 | 404 | | 2585 | | 84 | | 172 | 288 | |
| SAVLLFFNM | 36 | 15 | -0.494 | -0.68 | -0.871 | -2.012 | 33 | | 217 | | 376 | | 369 | 293 | |
| VQIDFFDNM | 24 | 1126 | -0.317 | -0.79 | -0.867 | -1.898 | 220 | | 146 | | 379 | | 555 | 299.5 | |
| VVGKNFEEM | 50 | 355 | -0.184 | -0.278 | -1.56 | -3.5 | 680 | | 554 | | 48 | | 41 | 301 | |
| VMMINANGV | 22 | 124 | -0.262 | 0.1 | -1.16 | -2.155 | 368 | | 1260 | | 157 | | 248 | 308 | |
| NSVHVQGVM | 43 | 57 | -0.378 | -1.213 | -0.003 | -1.933 | 111 | | 48 | | 2206 | | 507 | 309 | |
| VAVVSLLRL | 6 | 84 | -0.287 | -0.24 | -0.905 | -2.091 | 291 | | 601 | | 340 | | 287 | 315.5 | |
| KAMRLFNVM | 31 | 1188 | -0.603 | -0.37 | -1.085 | -1.494 | 8 | | 440 | | 195 | | 1574 | 317.5 | |
| AAVKSCEIL | 41 | 453 | -0.3 | -0.328 | -1.057 | -2.012 | 260 | | 489 | | 210 | | 376 | 318 | |
| FGLCVLSAI | 37 | 28 | -0.286 | -0.573 | -0.606 | -2.012 | 297 | | 288 | | 714 | | 352 | 324.5 | |
| AAVVAAESL | 40 | 9 | -0.06 | -1.068 | -0.98 | -2.012 | 1434 | | 77 | | 272 | | 377 | 324.5 | |
| AAIAPAAPV | 71 | 475 | -0.287 | -0.445 | -1.606 | -0.632 | 292 | | 368 | | 43 | | 4967 | 330 | |
| FALLAVGLL | 50 | 7 | 0.065 | -1.053 | -1.243 | -1.933 | 2739 | | 81 | | 119 | | 549 | 334 | |
| TMPGLQEPM | 71 | 554 | -0.514 | -0.197 | -1.138 | -1.933 | 25 | | 675 | | 172 | | 505 | 338.5 | |
| RVSRNKKTM | 24 | 723 | -0.228 | -0.524 | -0.883 | -3.301 | 488 | | 316 | | 366 | | 110 | 341 | |
| AAPVAPQQL | 71 | 480 | 0.094 | -0.521 | -1.165 | -2.012 | 3148 | | 319 | | 156 | | 373 | 346 | |
| IAVVFTPFV | 62 | 350 | -0.311 | -0.498 | -0.883 | -0.711 | 239 | | 332 | | 365 | | 4514 | 348.5 | |
| FMPAGSEAL | 49 | 101 | -0.196 | -0.706 | -1.768 | -1.933 | 619 | | 196 | | 24 | | 510 | 353 | |
| RTLVMQHFL | 31 | 978 | -0.311 | -1.372 | -0.784 | -1.857 | 240 | | 32 | | 467 | | 874 | 353.5 | |
| AALFGHPTL | 24 | 333 | 0.027 | -0.743 | -1.649 | -1.933 | 2293 | | 176 | | 36 | | 532 | 354 | |
| VALAFLLGL | 37 | 371 | -0.263 | -0.178 | -1.156 | -2.012 | 362 | | 712 | | 162 | | 356 | 359 | |
| VSVMGSTTL | 31 | 1178 | 0.149 | -0.71 | -1.054 | -1.933 | 4012 | | 194 | | 211 | | 513 | 362 | |
| LMPMVFRHM | 31 | 1351 | -0.289 | -1.069 | -0.4 | -1.971 | 285 | | 75 | | 1119 | | 443 | 364 | |
| LAFENAKDI | 46 | 350 | -0.263 | -0.454 | -0.775 | -3.494 | 366 | | 363 | | 476 | | 44 | 364.5 | |
| RLLQNSKSV | 3 | 630 | -0.214 | 0.125 | -1.206 | -2.556 | 543 | | 1334 | | 138 | | 188 | 365.5 | |
| KAVPTPASL | 64 | 212 | -0.487 | -1.412 | -0.187 | -1.892 | 36 | | 29 | | 1628 | | 701 | 368.5 | |
| LALVFLHHV | 31 | 15 | -0.22 | -0.664 | -1.216 | -0.749 | 521 | | 229 | | 130 | | 4357 | 375 | |
| AAVVAVVSL | 6 | 81 | 0.091 | -0.801 | -0.863 | -2.012 | 3098 | | 141 | | 387 | | 370 | 378.5 | |
| IAAMMTSPL | 48 | 38 | -0.261 | -1.447 | -0.837 | -1.456 | 373 | | 25 | | 406 | | 1797 | 389.5 | |
| WALRVLALV | 31 | 10 | -0.428 | -0.53 | -0.779 | -0.67 | 64 | | 312 | | 469 | | 4635 | 390.5 | |
| YALRAAIFL | 24 | 700 | -0.044 | -1.217 | -2.154 | -1.892 | 1572 | | 47 | | 5 | | 734 | 390.5 | |
| GALSALGAL | 71 | 647 | 0.058 | -0.812 | -0.933 | -1.971 | 2668 | | 136 | | 318 | | 469 | 393.5 | |
| LAIFSLGVL | 36 | 121 | -0.265 | -0.131 | -1.215 | -1.971 | 358 | | 793 | | 131 | | 433 | 395.5 | |
| KDLLNRSTL | 32 | 137 | -0.276 | -0.345 | -0.125 | -3.778 | 325 | | 471 | | 1826 | | 39 | 398 | |
| DALNTTEAM | 33 | 166 | -0.367 | -1.723 | -0.145 | -1.892 | 126 | | 8 | | 1762 | | 679 | 402.5 | |
| SGLLVLLTL | 33 | 10 | -0.241 | -0.338 | -0.873 | -2.012 | 446 | | 483 | | 373 | | 371 | 409.5 | |
| VMIVNVLAL | 6 | 11 | -0.571 | -1.571 | -2.474 | -4.012 | 14 | | 16 | | 1 | | 1 | 7.5 | |
| KAILNFVTV | 32 | 301 | -0.666 | -1.673 | -1.992 | -2.67 | 1 | | 11 | | 13 | | 159 | 12 | |
| IALYNRGHL | 22 | 134 | -0.315 | -1.522 | -1.967 | -3.933 | 227 | | 18 | | 15 | | 8 | 16.5 | |
| YALLNVADI | 24 | 1215 | -0.425 | -0.994 | -2.402 | -3.892 | 68 | | 88 | | 2 | | 14 | 41 | |
| LAFLNYLTI | 3 | 255 | -0.414 | -1.316 | -1.565 | -3.415 | 78 | | 36 | | 46 | | 69 | 57.5 | |
| AQLSTFLEL | 43 | 43 | -0.427 | -1.493 | -1.326 | -1.898 | 65 | | 20 | | 89 | | 633 | 77 | |
| AAVVFSHVL | 71 | 175 | -0.4 | -0.907 | -1.491 | -2.012 | 89 | | 104 | | 53 | | 354 | 96.5 | |
| FALFFVFAL | 65 | 26 | -0.441 | -0.807 | -1.766 | -1.933 | 58 | | 139 | | 25 | | 553 | 98.5 | |
| NKLMNALPM | 71 | 370 | -0.344 | -0.763 | -1.641 | -3.819 | 172 | | 162 | | 38 | | 24 | 100 | |
| SGVVNQGPV | 2 | 27 | -0.446 | -0.706 | -1.802 | -2.711 | 55 | | 197 | | 19 | | 150 | 102.5 | |
| LAVLNVALV | 63 | 28 | -0.37 | -0.979 | -1.354 | -2.591 | 123 | | 90 | | 76 | | 176 | 106.5 | |
| IQLLTLVAM | 51 | 199 | -0.52 | -2.05 | -1.061 | -1.898 | 21 | | 3 | | 205 | | 656 | 113 | |
| AAITKPLTI | 67 | 202 | -0.518 | -1.193 | -1.134 | -0.933 | 22 | | 52 | | 176 | | 3662 | 114 | |
| FSVKNSVFA | 65 | 19 | -0.017 | -0.964 | -1.678 | -2.711 | 1798 | | 93 | | 31 | | 149 | 121 | |
| VGLTNRSTL | 58 | 368 | -0.209 | -0.635 | -2.229 | -3.933 | 560 | | 247 | | 4 | | 7 | 127 | |
| ASWNNSYTL | 68 | 73 | -0.354 | -0.766 | -1.22 | -3.456 | 143 | | 160 | | 129 | | 46 | 136 | |
| DAGANALFI | 72 | 222 | -0.417 | -0.698 | -0.973 | -3.415 | 75 | | 199 | | 279 | | 60 | 137 | |
| AVLHNLRQL | 24 | 193 | -0.236 | -0.747 | -1.288 | -3.898 | 465 | | 172 | | 104 | | 11 | 138 | |
| LAILNEFFA | 16 | 89 | 0.011 | -0.834 | -1.528 | -2.591 | 2110 | | 120 | | 50 | | 179 | 149.5 | |
| RAPENVKLL | 23 | 511 | -0.194 | -0.544 | -1.735 | -3.971 | 635 | | 304 | | 29 | | 4 | 166.5 | |
| KTVVNLTEV | 62 | 255 | -0.431 | -0.725 | -0.753 | -2.635 | 63 | | 186 | | 510 | | 163 | 174.5 | |
| DQPGNHQAL | 18 | 30 | -0.344 | -0.435 | -1.118 | -3.778 | 169 | | 377 | | 183 | | 37 | 176 | |
| VAAGNPSAV | 16 | 55 | -0.448 | -0.474 | -1.28 | -2.155 | 52 | | 351 | | 105 | | 254 | 179.5 | |
| ASSDNGATL | 12 | 242 | -0.325 | -0.095 | -1.149 | -3.456 | 201 | | 866 | | 168 | | 47 | 184.5 | |
| ASVAMLTCM | 3 | 404 | -0.589 | -2.513 | -0.512 | -2.012 | 10 | | 1 | | 870 | | 368 | 189 | |
| TSKKNLKAM | 24 | 1138 | -0.38 | -0.603 | -0.648 | -3.091 | 107 | | 267 | | 647 | | 115 | 191 | |
| SSVYNYYDI | 31 | 1643 | -0.283 | -1.01 | -0.889 | -3.933 | 308 | | 83 | | 358 | | 6 | 195.5 | |
| QALGNSERL | 63 | 216 | -0.28 | -0.821 | -0.985 | -3.892 | 313 | | 128 | | 263 | | 13 | 195.5 | |
| GTVTNPLAI | 59 | 86 | -0.358 | -0.613 | -0.753 | -3.778 | 138 | | 261 | | 509 | | 28 | 199.5 | |
| FAVKHCLLV | 2 | 2 | -0.335 | -0.801 | -1.052 | -0.711 | 186 | | 142 | | 214 | | 4506 | 200 | |
| NSAENTWFI | 68 | 265 | -0.338 | -0.458 | -1.046 | -3.535 | 182 | | 360 | | 219 | | 40 | 200.5 | |
| IALAKLHEL | 22 | 285 | -0.315 | -0.738 | -1.467 | -1.012 | 226 | | 178 | | 59 | | 3554 | 202 | |
| AMLTCMAFV | 3 | 407 | -0.617 | -0.666 | -1.127 | -0.632 | 5 | | 227 | | 181 | | 4960 | 204 | |
| NAENNSLTI | 62 | 218 | -0.273 | -0.794 | -0.981 | -2.757 | 332 | | 144 | | 270 | | 141 | 207 | |
| SQIRTRTEV | 35 | 290 | -0.332 | -1.446 | -1.003 | -0.518 | 190 | | 26 | | 248 | | 6016 | 219 | |
| FMVPSDVVM | 63 | 322 | -0.452 | -0.877 | -0.913 | -1.933 | 50 | | 108 | | 335 | | 501 | 221.5 | |
| SAQDNYSFL | 36 | 812 | -0.261 | -0.492 | -1.258 | -3.456 | 371 | | 338 | | 115 | | 48 | 226.5 | |
| AAVQTGRDL | 36 | 478 | -0.401 | -1.62 | -0.18 | -2.012 | 88 | | 15 | | 1650 | | 367 | 227.5 | |
| RAAVTGHPV | 6 | 65 | -0.38 | -1.118 | -0.886 | -0.193 | 106 | | 67 | | 359 | | 7333 | 232.5 | |
| QAVQVLAQL | 32 | 445 | -0.349 | -0.853 | -0.102 | -2.05 | 161 | | 115 | | 1892 | | 305 | 233 | |
| SKNANFIEI | 37 | 227 | -0.259 | 0.485 | -1.33 | -3.421 | 382 | | 2509 | | 87 | | 55 | 234.5 | |
| TTLTNAALI | 3 | 494 | -0.361 | -0.077 | -0.897 | -3.819 | 134 | | 902 | | 350 | | 26 | 242 | |
| MAPYLMLPM | 24 | 1277 | -0.396 | -0.424 | -1.562 | -1.892 | 92 | | 393 | | 47 | | 688 | 242.5 | |
| AAAATLIAL | 53 | 233 | -0.635 | -1.417 | -0.788 | -1.535 | 3 | | 28 | | 459 | | 1406 | 243.5 | |
| AQLKKVGAM | 26 | 234 | -0.555 | -1.271 | -0.796 | -0.898 | 18 | | 41 | | 449 | | 3701 | 245 | |
| SNLVSLGAM | 61 | 241 | -0.436 | 0.29 | -1.378 | -1.977 | 59 | | 1813 | | 71 | | 421 | 246 | |
| LAPQMPTPV | 71 | 422 | -0.259 | -1.81 | -1.235 | -0.67 | 381 | | 6 | | 122 | | 4687 | 251.5 | |
| NVIVNDGAL | 71 | 288 | -0.316 | -0.582 | -0.852 | -3.898 | 225 | | 281 | | 395 | | 10 | 253 | |
| AAPTQLTPL | 71 | 512 | -0.352 | 0.377 | -1.812 | -2.012 | 147 | | 2109 | | 18 | | 361 | 254 | |
| ATARNQGFL | 24 | 1039 | -0.512 | -0.235 | -0.818 | -3.342 | 27 | | 616 | | 423 | | 86 | 254.5 | |
| LAVVVAAVL | 7 | 14 | -0.464 | -1.075 | -0.636 | -1.971 | 42 | | 73 | | 664 | | 438 | 255.5 | |
| AAVLLLITV | 26 | 10 | -0.561 | -0.749 | -0.903 | -0.711 | 16 | | 171 | | 342 | | 4540 | 256.5 | |
| RSVQVSGAL | 29 | 203 | -0.434 | -1.069 | -0.29 | -1.971 | 60 | | 76 | | 1369 | | 449 | 262.5 | |
| AALQTVHQL | 26 | 89 | -0.288 | -1.803 | -1.013 | -2.012 | 287 | | 7 | | 239 | | 363 | 263 | |
| GSVVNIPDI | 58 | 98 | -0.139 | -0.443 | -1.154 | -3.971 | 900 | | 371 | | 165 | | 3 | 268 | |
| FSVVNTHGA | 3 | 541 | 0.031 | -0.398 | -1.263 | -2.711 | 2335 | | 409 | | 112 | | 147 | 278 | |
| SGIAMQQVL | 15 | 183 | -0.32 | -1.064 | -0.902 | -1.933 | 213 | | 78 | | 345 | | 534 | 279 | |
| VMITLLFQL | 31 | 367 | -0.319 | -0.06 | -1.077 | -2.012 | 215 | | 926 | | 198 | | 350 | 282.5 | |
| MQIPNCEKI | 34 | 401 | -0.254 | -0.749 | -0.503 | -3.778 | 398 | | 170 | | 890 | | 35 | 284 | |
| YGNLNNNVM | 72 | 332 | -0.222 | 0.061 | -1.49 | -3.415 | 516 | | 1179 | | 54 | | 57 | 286.5 | |
| TLPENKATV | 64 | 470 | -0.252 | 0.501 | -1.337 | -2.597 | 404 | | 2585 | | 84 | | 172 | 288 | |
| SAVLLFFNM | 36 | 15 | -0.494 | -0.68 | -0.871 | -2.012 | 33 | | 217 | | 376 | | 369 | 293 | |
| VQIDFFDNM | 24 | 1126 | -0.317 | -0.79 | -0.867 | -1.898 | 220 | | 146 | | 379 | | 555 | 299.5 | |
| VVGKNFEEM | 50 | 355 | -0.184 | -0.278 | -1.56 | -3.5 | 680 | | 554 | | 48 | | 41 | 301 | |
| VMMINANGV | 22 | 124 | -0.262 | 0.1 | -1.16 | -2.155 | 368 | | 1260 | | 157 | | 248 | 308 | |
| NSVHVQGVM | 43 | 57 | -0.378 | -1.213 | -0.003 | -1.933 | 111 | | 48 | | 2206 | | 507 | 309 | |
| VAVVSLLRL | 6 | 84 | -0.287 | -0.24 | -0.905 | -2.091 | 291 | | 601 | | 340 | | 287 | 315.5 | |
| KAMRLFNVM | 31 | 1188 | -0.603 | -0.37 | -1.085 | -1.494 | 8 | | 440 | | 195 | | 1574 | 317.5 | |
| AAVKSCEIL | 41 | 453 | -0.3 | -0.328 | -1.057 | -2.012 | 260 | | 489 | | 210 | | 376 | 318 | |
| FGLCVLSAI | 37 | 28 | -0.286 | -0.573 | -0.606 | -2.012 | 297 | | 288 | | 714 | | 352 | 324.5 | |
| AAVVAAESL | 40 | 9 | -0.06 | -1.068 | -0.98 | -2.012 | 1434 | | 77 | | 272 | | 377 | 324.5 | |
| AAIAPAAPV | 71 | 475 | -0.287 | -0.445 | -1.606 | -0.632 | 292 | | 368 | | 43 | | 4967 | 330 | |
| FALLAVGLL | 50 | 7 | 0.065 | -1.053 | -1.243 | -1.933 | 2739 | | 81 | | 119 | | 549 | 334 | |
| TMPGLQEPM | 71 | 554 | -0.514 | -0.197 | -1.138 | -1.933 | 25 | | 675 | | 172 | | 505 | 338.5 | |
| RVSRNKKTM | 24 | 723 | -0.228 | -0.524 | -0.883 | -3.301 | 488 | | 316 | | 366 | | 110 | 341 | |
| AAPVAPQQL | 71 | 480 | 0.094 | -0.521 | -1.165 | -2.012 | 3148 | | 319 | | 156 | | 373 | 346 | |
| IAVVFTPFV | 62 | 350 | -0.311 | -0.498 | -0.883 | -0.711 | 239 | | 332 | | 365 | | 4514 | 348.5 | |
| FMPAGSEAL | 49 | 101 | -0.196 | -0.706 | -1.768 | -1.933 | 619 | | 196 | | 24 | | 510 | 353 | |
| RTLVMQHFL | 31 | 978 | -0.311 | -1.372 | -0.784 | -1.857 | 240 | | 32 | | 467 | | 874 | 353.5 | |
| AALFGHPTL | 24 | 333 | 0.027 | -0.743 | -1.649 | -1.933 | 2293 | | 176 | | 36 | | 532 | 354 | |
| VALAFLLGL | 37 | 371 | -0.263 | -0.178 | -1.156 | -2.012 | 362 | | 712 | | 162 | | 356 | 359 | |
| VSVMGSTTL | 31 | 1178 | 0.149 | -0.71 | -1.054 | -1.933 | 4012 | | 194 | | 211 | | 513 | 362 | |
| LMPMVFRHM | 31 | 1351 | -0.289 | -1.069 | -0.4 | -1.971 | 285 | | 75 | | 1119 | | 443 | 364 | |
| LAFENAKDI | 46 | 350 | -0.263 | -0.454 | -0.775 | -3.494 | 366 | | 363 | | 476 | | 44 | 364.5 | |
| RLLQNSKSV | 3 | 630 | -0.214 | 0.125 | -1.206 | -2.556 | 543 | | 1334 | | 138 | | 188 | 365.5 | |
| KAVPTPASL | 64 | 212 | -0.487 | -1.412 | -0.187 | -1.892 | 36 | | 29 | | 1628 | | 701 | 368.5 | |
| LALVFLHHV | 31 | 15 | -0.22 | -0.664 | -1.216 | -0.749 | 521 | | 229 | | 130 | | 4357 | 375 | |
| AAVVAVVSL | 6 | 81 | 0.091 | -0.801 | -0.863 | -2.012 | 3098 | | 141 | | 387 | | 370 | 378.5 | |
| IAAMMTSPL | 48 | 38 | -0.261 | -1.447 | -0.837 | -1.456 | 373 | | 25 | | 406 | | 1797 | 389.5 | |
| WALRVLALV | 31 | 10 | -0.428 | -0.53 | -0.779 | -0.67 | 64 | | 312 | | 469 | | 4635 | 390.5 | |
| YALRAAIFL | 24 | 700 | -0.044 | -1.217 | -2.154 | -1.892 | 1572 | | 47 | | 5 | | 734 | 390.5 | |
| GALSALGAL | 71 | 647 | 0.058 | -0.812 | -0.933 | -1.971 | 2668 | | 136 | | 318 | | 469 | 393.5 | |
| LAIFSLGVL | 36 | 121 | -0.265 | -0.131 | -1.215 | -1.971 | 358 | | 793 | | 131 | | 433 | 395.5 | |
| KDLLNRSTL | 32 | 137 | -0.276 | -0.345 | -0.125 | -3.778 | 325 | | 471 | | 1826 | | 39 | 398 | |
| DALNTTEAM | 33 | 166 | -0.367 | -1.723 | -0.145 | -1.892 | 126 | | 8 | | 1762 | | 679 | 402.5 | |
| SGLLVLLTL | 33 | 10 | -0.241 | -0.338 | -0.873 | -2.012 | 446 | | 483 | | 373 | | 371 | 409.5 | |
